# Fungal endophytes from arid areas of Andalusia: high potential sources for antifungal and antitumoral agents

**DOI:** 10.1038/s41598-018-28192-5

**Published:** 2018-06-27

**Authors:** Victor González-Menéndez, Gloria Crespo, Nuria de Pedro, Caridad Diaz, Jesús Martín, Rachel Serrano, Thomas A. Mackenzie, Carlos Justicia, M. Reyes González-Tejero, M. Casares, Francisca Vicente, Fernando Reyes, José R. Tormo, Olga Genilloud

**Affiliations:** 1 Fundación MEDINA, Avda. del conocimiento 34, 18016 Granada, Spain; 2 Departamento de Botánica, Facultad de Farmacia, Universidad de Granada, C/ Prof. Clavera, s/n, 18011 Granada, Spain

## Abstract

Native plant communities from arid areas present distinctive characteristics to survive in extreme conditions. The large number of poorly studied endemic plants represents a unique potential source for the discovery of novel fungal symbionts as well as host-specific endophytes not yet described. The addition of adsorptive polymeric resins in fungal fermentations has been seen to promote the production of new secondary metabolites and is a tool used consistently to generate new compounds with potential biological activities. A total of 349 fungal strains isolated from 63 selected plant species from arid ecosystems located in the southeast of the Iberian Peninsula, were characterized morphologically as well as based on their ITS/28S ribosomal gene sequences. The fungal community isolated was distributed among 19 orders including Basidiomycetes and Ascomycetes, being *Pleosporales* the most abundant order. In total, 107 different genera were identified being *Neocamarosporium* the genus most frequently isolated from these plants, followed by *Preussia* and *Alternaria*. Strains were grown in four different media in presence and absence of selected resins to promote chemical diversity generation of new secondary metabolites. Fermentation extracts were evaluated, looking for new antifungal activities against plant and human fungal pathogens, as well as, cytotoxic activities against the human liver cancer cell line HepG2. From the 349 isolates tested, 126 (36%) exhibited significant bioactivities including 58 strains with exclusive antifungal properties and 33 strains with exclusive activity against the HepG2 hepatocellular carcinoma cell line. After LCMS analysis, 68 known bioactive secondary metabolites could be identified as produced by 96 strains, and 12 likely unknown compounds were found in a subset of 14 fungal endophytes. The chemical profiles of the differential expression of induced activities were compared. As proof of concept, ten active secondary metabolites only produced in the presence of resins were purified and identified. The structures of three of these compounds were new and herein are elucidated.

## Introduction

Several bioprospecting reports have published the ability of endophytic fungi to produce a broad range of bioactive secondary metabolites. Many of them are used as sources of anticancer lead compounds (i.e. taxol, vincristine, vinblastine, camptothecin and podophyllotoxin)^1^ or as sources of antifungal lead molecules (i.e., cryptocandin A^2^, enfumafungin^3^, CR377^4^, ambuic acid^5^, jesterone^6^, moriniafungin^7^, parnafungins^8^ or phaeofungin^9^).

Fungal endophytes represent one of the most prolific sources of novel natural products but, low production yields and the lack of expression of cryptic gene clusters in laboratory conditions are frequently key limiting factors to exploit their high potential. Several strategies have been developed to address these issues, including mutagenesis, genetic transformation, agar co-cultivation, mixed-culture fermentations and the use of additives, such as epigenetic modifiers or adsorptive polymeric resins^10^. We recently reported the promotion of new chemical entities by the addition of Diaion^®^ and Amberlite^®^ resins to arrays of fungal fermentation media. Results concluded that this approach can induce the production of new secondary metabolites and affect consistently the production yields, at least for specific groups of fungal strains^11^.

The Iberian southeast is one of the most arid regions in Europe. It is characterized by a warm and dry Mediterranean climate and it is rich in gypsum and saline soils. These unique ecological conditions have allowed the development of a wide range of endemic plant species where it is frequent to find specimens of the *Aizoaceae*, *Chenopodiaceae* or *Plumbaginaceae* families^12^ which are barely represented outside of these environments^13^.

The diversification of Ascomycota, the highest speciated fungal phylum, is reflected in its multiple symbiotic strategies with vascular plants, and symbiotic lifestyles (mutualistic, commensalistic, parasitic and pathogenic) in response to host genotype and environmental factors^14^. Fungal endophytes are mostly known to infect plants without causing symptoms. Latent pathogens seem to represent a relatively small proportion of the endophytic community, including latent saprophytes and mutualistic species^15^. A partial characterization of Ascomycotina from arid zones of Almeria and Baetic mountains (Andalusia) indicates that fungi associated to plants of these areas remain to be discovered^16^. Limited information is still available on the biodiversity of fungal symbionts and host-specific endophytes in these areas, including the role of these microorganisms in the ecological fitness and survival of arid plants.

Previous studies on fungal endophytes from plants of arid areas^17^ have not described their potential to generate chemical diversity, they only provided scattered examples of these populations within specific medicinal plant hosts^18,19^ and mentioned their bioactive potential as a whole. With this in mind, we decided to perform a broader survey in Andalusia, one of the richest areas in arid plant endemisms of Europe, isolating and characterizing both, morphologically and chemically these endophytes and their potential to generate new bioactive secondary metabolites by selecting the best combinations of media and resins to promote their chemical diversity generation based on previous results^11^.

## Results

### Biodiversity of fungal isolates

From the individual plant species collected (63) a total of 349 fungal strains were isolated. 310 were obtained from surface-disinfected leaf or stem pieces, 32 were directly isolated from fungal reproductive structures on stems, and 7 were isolated directly from cleistothecia and/or conidiophores, which developed on plant material after incubation in moist chambers. Given the major interest in low occurring fungal species colonizing these substrates, 286 strains (45.0%) from dominant species such as *Cladosporium*, *Penicillium* and *Aspergillus* were not considered in further studies as they are generally considered as ubiquitous epiphytes.

The remaining isolates were distributed among 19 orders including Basidiomycetes and Ascomycetes (Supplementary Information Fig. S2). More than half of the isolates (208; 59.6%) were included within the *Pleosporales* order, followed by *Dothideales* (22; 6.0%) and *Xylariales* (19; 5.5%). Within the *Pleosporales* order, our isolates were distributed among 42 genera belonging to 14 families, being the *Pleosporaceae*, *Didymellaceae* and *Sporormiaceae* the most abundant with 76, 43 and 19 isolates respectively. In total, 107 different genera were identified being the 10 genera with the highest number of isolates: *Neocamarosporium* (37), *Preussia* (19), *Alternaria* (18), *Ascochyta* (17), *Phoma* (14), *Comoclathris* (13), *Neomicrosphaeropsis* (10), *Aureobasidium* (7), *Pleospora* (7) and *Fusarium* (7) (see full list in Supplementary Information, Table S1).

### Phylogenetic analyses

Phylogenetic analysis of the isolates belonging to the above-mentioned genera within the *Pleosporales* order were based on ITS/28S rDNA excluding *Preussia* which was the subject of a specific review study recently published by our group^20^. The different runs of the Bayesian and ML analyses yielded the same topology. The consensus phylogenetic tree of the 130 isolated strains with 95 GenBank™ sequences of representative strains (see Table S2 in Supplementary information) included the endophytic strains isolated recently from other plants of the Arizona desert^17,21^. The resulting tree also showed a very similar topology to the phylogenetic trees obtained recently in other *Pleosporales* characterization studies^22–24^.

**Figure 1:**
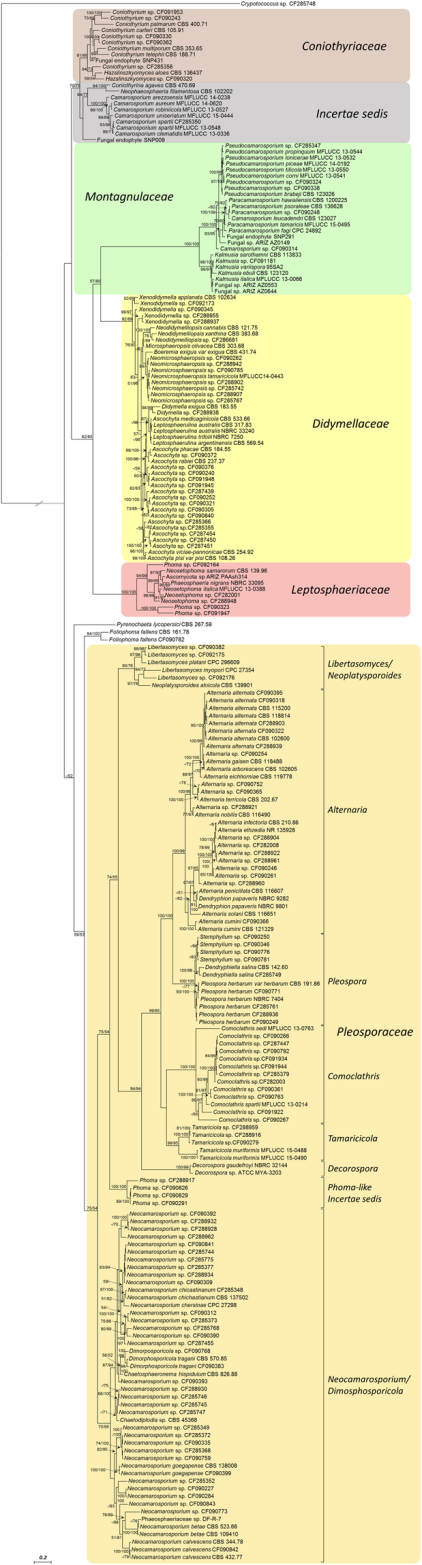
Consensus tree from Bayesian-phylogeny inferences based on ITS/28S sequences of almost all isolated genera within *Pleosporales* order and related genera. Clade probability values/maximum likelihood bootstrap values are indicated respectively on the branches. Values < 50 are designated by “−”. *Cryptococcus* sp. CF-285748 was used as an outgroup.

The ITS/28S rDNA tree revealed six main clades (Fig. 1): The *Coniothyriaceae* clade clustered *Coniothyrium* and *Hazslinszkyomyces* species and included six of our isolates supported statistically (posterior probability values = 91%/maximum likelihood bootstrap = 95%); The *Incertae sedis* clade grouped *Camarosporium* species including the *Camarosporium* isolate CF-285350 with high statistical support (pp = 100%/bs = 100%); The highly supported *Montagnulacea* clade grouped three different genera, *Pseudocamarosporium* (pp = 100%/bs = 98%), *Paracamarosporium* (pp = 100%/bs = 100%) and *Kalmusia* (pp = 99%/bs = 97%) including six of our isolates; The *Didymellaceae* clade, including species of *Xenodidymella*, *Neodidymelliopsis*, *Neomicrosphaeropsis*, *Didymella*, *Leptosphaerulina* and *Ascochyta* grouped 28 of our isolates with moderate statistical support (pp = 92%/bs = 89%); The highly supported *Leptosphaeriaceae* clade (pp = 100%/bs = 100%) included the *Neosetophoma* species and three *Phoma*-like isolates (CF-092164, CF-090312 and CF-091947); And the *Pleosporaceae* clade that was the largest one and clustered eight subclades including 82 of our isolates: *Libertasomyces/Neoplatysporoide*s, *Alternaria*, *Pleospora*, *Comoclathris*, *Tamaricicola*, *Decorospora*, *Phoma*-like and *Neocamarosporium*/*Dimorphosporicola*, all supported statistically (pp = 95–100%/bs > 70%) and where *Neocamarosporium* was the most representative genera with 35 isolates.

### Activity distribution of fungal isolates

**Figure 2:**
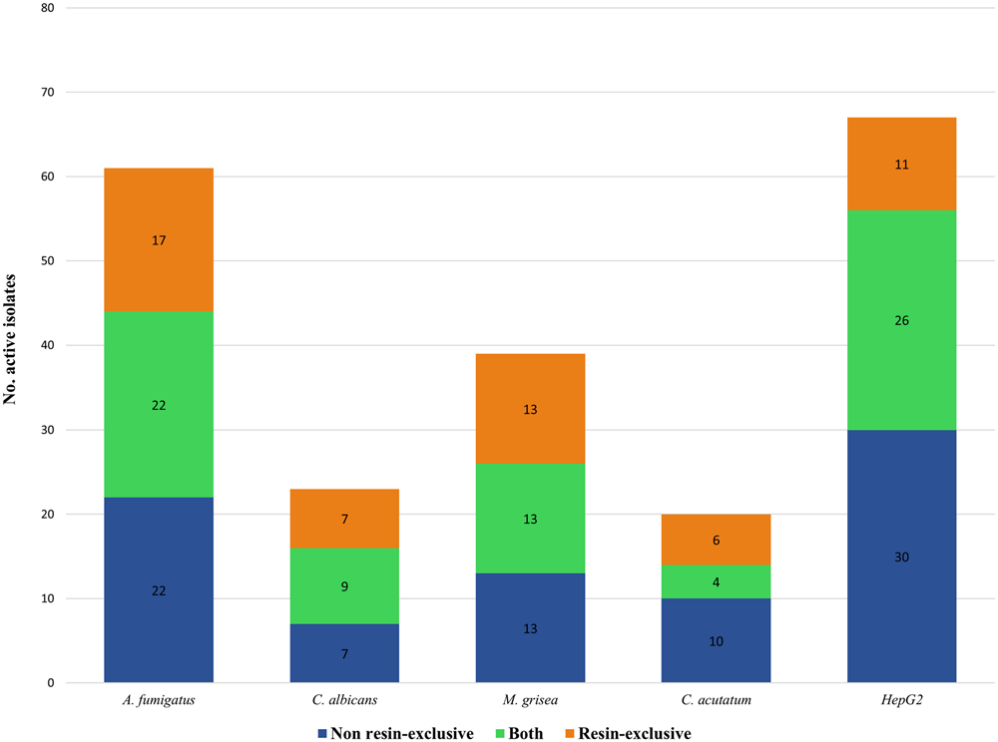
Activity distribution of isolates according to the fermentation conditions on each of the assays: (i) hits induced by adsorptive resins (orange), (ii) hits only produced without resins (blue) or (iii) hits produced in both conditions (green).

The fungal endophytes population was explored for their ability to generate antifungal and antitumoral activities. For this purpose, all 349 fungal isolates were grown in four media with and without the addition of polymeric resins. The corresponding 2792 crude extracts were tested against two fungal plant pathogens (*Magnaporthe grisea* and *Colletotrichum acutatum*), and two human fungal pathogens (*Aspergillus fumigatus* and *Candida albicans)*. Activities from these isolates were then classified into three groups according to their fermentation conditions: (i) hits induced by the use of polymeric resins, (ii) hits only produced without the resins and (iii) hits generated in both conditions (Fig. 2); we also grouped the strains according to their activity spectra in cytotoxic/non-cytotoxic or in broad/specific antifungal spectra (see Table S3 in Supplementary Information).

From the 349 isolates tested, 126 exhibited significant activity using the classical ‘One Strain Many Conditions’ (OSMAC) approach^25^. Among them, 35 isolates showed both cytotoxic and antifungal activities in multiple assays, and as many as three strains were found to be active in all assays tested (*Albifimbria verrucaria* CF-285778, and *Trichothecium roseum* CF-285757 and CF-277739). These strains produced several toxins in each fermentation condition tested. Toxins were identified as verrucarin, roridin, iludin, and trichothecene when analyzed by mass spectrometry. Regarding the generation of antifungal activities against plant pathogens, 39 isolates showed inhibition zones larger than 6 mm of diameter against the rice phytopathogen *Magnaporthe grisea* in agar-based assays. For the bioassay of the fruit pathogen *Colletotrichum acutatum* a total of 20 isolates produced inhibition halos with more than 6 mm of diameter. When evaluated against human fungal pathogens, 61 fungal isolates showed more than 70% of growth inhibition against the opportunistic fungus *Aspergillus fumigatus* ATCC 46645 and 23 fungal isolates presented inhibition of the dimorphic fungus *Candida albicans* MY1055. The detailed distribution of activities induced by the addition of polymeric resins is described in Fig. 2.

In order to evaluate the production of antitumoral/cytotoxic compounds, all fungal isolates were also tested against the human hepatocellular carcinoma cell line HepG2 ATCC HB 8065. Among them, a total of 67 isolates showed HepG2 cell proliferation inhibitions higher than 70%, only 11 presented activity when grown in the presence of resins (six of them without antifungal activity), 30 when cultivated without the addition of resins (14 non-antifungal) and 26 isolates when grown in both conditions (23 with no antifungal activity). Regarding the non-cytotoxic strains, 16 presented complex antifungal spectra: *Stagonospora* sp. CF-281556 and *Camarosporium* sp. CF-090324 showed a broad spectrum against all fungal pathogens tested; *Fusarium equiseti* CF-285462, *Neocamarosporium* spp. CF090393 and CF-285768, *Phoma* sp. CF-285355 and *Pleospora* sp. CF-090792 showed a partial antifungal spectrum against human and plant pathogens; *Phaeosphaeria* sp. CF-288952 was active only against plant pathogens; *Comoclathris* sp. CF-090267, *Cryptococcus* sp. CF-285752, *Hormonema carpetanum* CF-090352, *Phaeotheca triangularis* CF-285358, *Phoma macrostoma* CF-287454, *Preussia australis* CF-288933 and *Xylaria* sp. CF-285461 were active only against the two human pathogens tested; Whereas, the remaining 75 strains showed specific activity against single fungal pathogens or the HepG2 cancer cell line (among them, 22 strains produced specific activity only when grown in the presence of resins, 31 only when grown without resins and 22 in both conditions; See Table S3 in Supplementary Information).

### Chemical dereplication of active extracts

**Figure 3:**
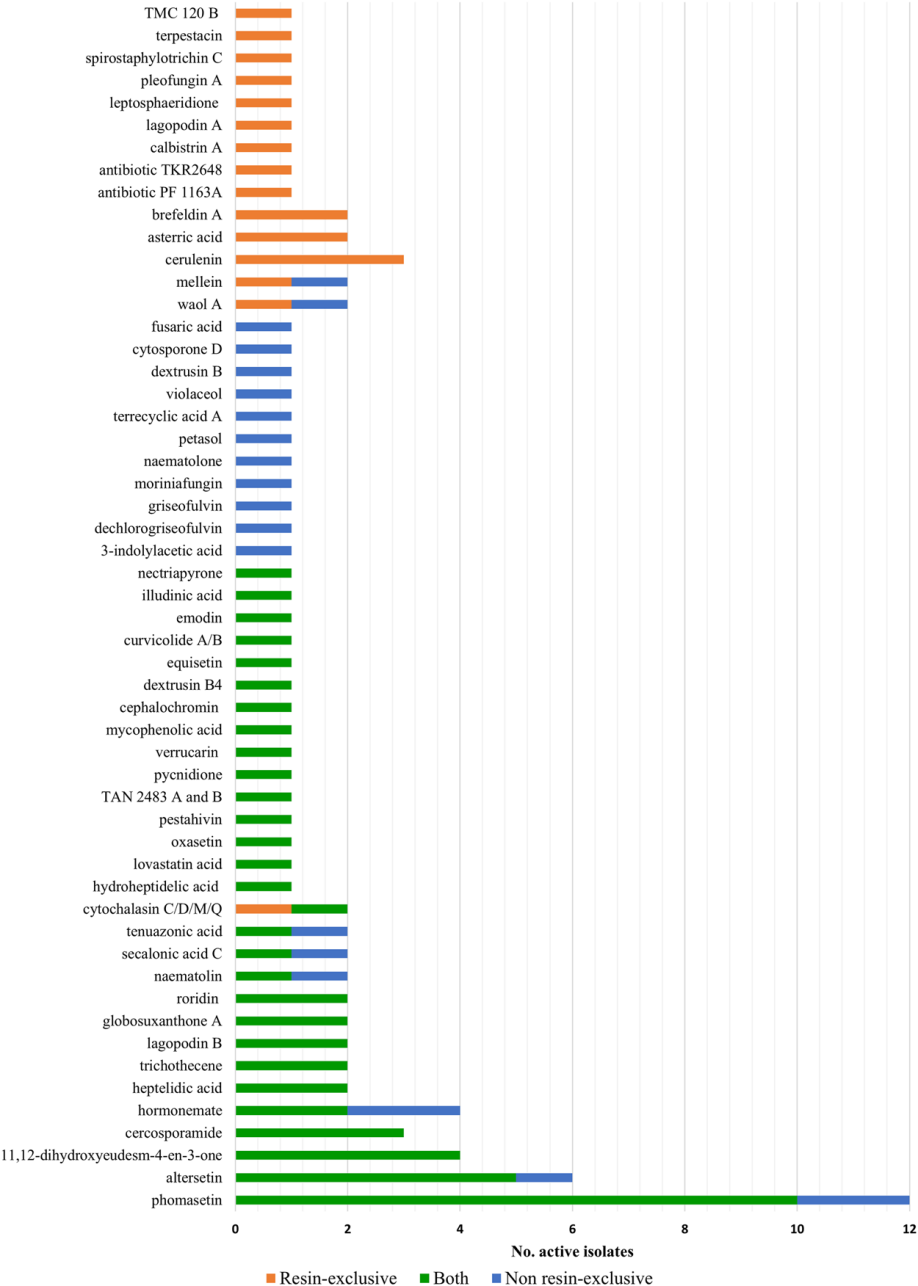
Distribution of dereplicated known fungal compounds in active isolates according to the fermentation condition of the producers. (i) Compounds detected in the presence of adsorptive resins (orange), (ii) compounds only produced without resins (blue) and (iii) compounds produced in both conditions (green).

Regarding the distribution of known bioactive compounds among the active strains, active extracts from 126 strains were dereplicated by LC-MS against MEDINA’s internal databases of known natural products by fingerprint matching of their HPLC retention time, ultraviolet and mass spectroscopy data. Fifty-four known bioactive molecules were identified from 75 strains both in resin- exclusive and non-exclusive production conditions which are represented in Fig. 3.

The extracts from the 51 active strains that did not present any matches in our databases were further analyzed by HR-MS (high resolution mass spectroscopy) for the tentative identification of additional molecules by matching their predicted molecular formula with the updated Dictionary of Natural Products databases for fungal natural products (DNP)^26^. Ascochlorin, coriolide, cerebroside B, solanopyrone B, brefeldin A, naematolin, ACTG toxin C, phaeosphenone, ilicicolin H, phomotone, phenopyrrozin, taurocholic acid, chrysoxanthone and sequalestatin H1were identified as produced by only 21 isolates (see Supplementary Table S3). We did not find any known molecules that could explain the activity observed in the remaining 30 active strains. In 16 cases we found predicted molecular formulas that had only been described previously in plant extracts and for the remaining 14 strains we found molecules whose molecular formulas did not show any coincidence in the DNP, with high chances of corresponding to bioactive molecules not yet described (Table 1).

**Table 1:** Characterization of tentative new secondary metabolites by HPLC-ESI-TOF-MS.

Strain	RT (min)	[M + H]^+^ Exp.	Proposed Ion	Main Secondary Experimental Ions	Production Media	Proposed Formula	Compound
CF-285754	3.91	497.11	C_22_H_25_O_11_S^+^	498.1138; 183.1372; 295.2259	LSFM + XAD16	C_22_H_24_O_11_S	A
CF-285755	3.92	497.1104	C_22_H_25_O_11_S^+^	498.1138; 271.0587; 183.1217	LSFM + XAD16	C_22_H_24_O_11_S	
CF-285753	3.91	497.1102	C_22_H_25_O_11_S^+^	498.1135; 499.1105; 519.0918	LSFM + XAD16	C_22_H_24_O_11_S	
CF-288957	5.04	656.3848	C_30_H_55_O_14_^+^	657.3879; 658.39	YES & HP20	C_30_H_54_O_14_	B
CF-090351	5.01	656.3852	C_30_H_55_O_14_^+^	657.3885; 658.3911	MMK2 & XAD16	C_30_H_54_O_14_	
CF-091924	5.33	933.5659	C_46_H_74_N_7_O_12_^+^	916.5391; 458.7725; 934.5688	YES & HP20	C_46_H_73_N_7_O_12_	C
CF-091924	5.49	947.5815	C_47_H_76_N_7_O_12_^+^	930.5549; 948.5846; 931.5579	YES & HP20	C_47_H_75_N_7_O_12_	D
CF-091924	6.11	914.5596	C_47_H_76_N_7_O_11_^+^	457.7832; 931.5863; 932.5891	YES & HP20	C_47_H_75_N_7_O_11_	E
CF-090782	5.08	328.2475	C_39_H_28_NO_8_S^+^	293.2104; 329.2508; 275.1997	MMK2 + XAD16	C_39_H_27_NO_8_S	F
CF-288938	4.65	328.2471	C_39_H_28_NO_8_S^+^	275.1994; 311.2206; 279.2308	MMK2	C_39_H_27_NO_8_S	
CF-287465	6.27	780.5469	C_40_H_75_O_13_^+^	781.5503; 745.5095; 785.5019	LSFM & XAD16	C_40_H_74_O_13_	G
CF-285758	4.85	415.1392	C_22_H_23_O_8_^+^	416.1421; 359.1124; 301.0704	YES + HP20	C_22_H_22_O_8_	H
CF-285760	2.61	521.2349	C_18_H_33_N_8_O_10_^+^	522.2374; 538.2608; 523.2389	MMK2	C_18_H_32_N_8_O_10_	I
CF-285773	2.54	521.2335	C_18_H_33_N_8_O_10_^+^	522.2363; 539.2626; 523.239	XPMK + HP20	C_18_H_32_N_8_O_10_	
CF-285765	5.54	551.1503	C_36_H_23_O_6_^+^	568.1767; 552.1532; 559.1794	YES	C_36_H_22_O_6_	J
CF-090361	3.76	343.1926	C_18_H_31_O_4_S^+^	235.2048; 344.1957; 943.5241	LSFM& MMK2	C_18_H_30_O_4_S	K
CF-090361	7.99	532.439	C_30_H_59_O_4_S^+^	515.4127; 532.44226; 516.416	LSFM & MMK2	C_30_H_58_O_4_S	L

Regarding the distribution of the known bioactive compounds among the fungal isolates, 35 compounds were detected in single strains whereas 19 were found in more than one strain. Among all these compounds, 12 were only detected in fermentations performed in presence of resins (Fig. 3). In general, phomasetin (C_25_H_35_NO_4_) was the most frequent compound detected, being produced by 12 different strains, including four *Comoclatrhis* (CF-090792, CF-091944, CF-282003, CF-287447), two *Neomicrosphaeropsis* (CF-285741, CF-285767), one *Neo*camarosporium CF-090228, one *Pleospora* sp. CF-091933, one *Pleiochaeta* sp. CF-285364, one unidentified *Pleosporales* CF-282344 and one *Leptosphaeria hispanica* CF-090357. This tetramic acid, isolated from *Phoma* sp., is closely related to equisetin, but with opposite stereochemistry^27^. The related compound, altersetin^28^, was found to be produced by the four *Comoclathris* strains and the *Pleospora* sp. On the other hand, cercosporamide, a selective antifungal inhibitor of Pkc1 kinase^29^, was produced by three different *Phoma-*like strains (CF-285355, CF-287454, CF-285365).

### Differential expression of antifungal activities

The addition of adsorptive polymeric resins during fungal fermentations produced an increase in antifungal activities ranging from 20% (against *C*. *acutatum*) to 36% (against *A*. *fumigatus*). In order to characterize, in detail, the chemical diversity of the active isolates when antifungal activities were induced by the addition of resins (Fig. 3), secondary metabolite (SMs) profiles from fermentations generated with and without the resins were compared, and known bioactive molecules were identified by matching LC/MS databases.

In the presence of resins, 37 isolates presented antifungal activity (see Table 3 in Supplementary Information) and five of them resulted to be active against more than one fungal pathogen. Five isolates showed chemical profiles containing peaks not observed in the strains cultivated without resin, and 14 had components not described as known antifungals in the DNP database. For the remaining 12 isolates, increased production titers were observed in the presence of resins, and five produced compounds not described as known antifungals in DNP database.

**Figure 4:**
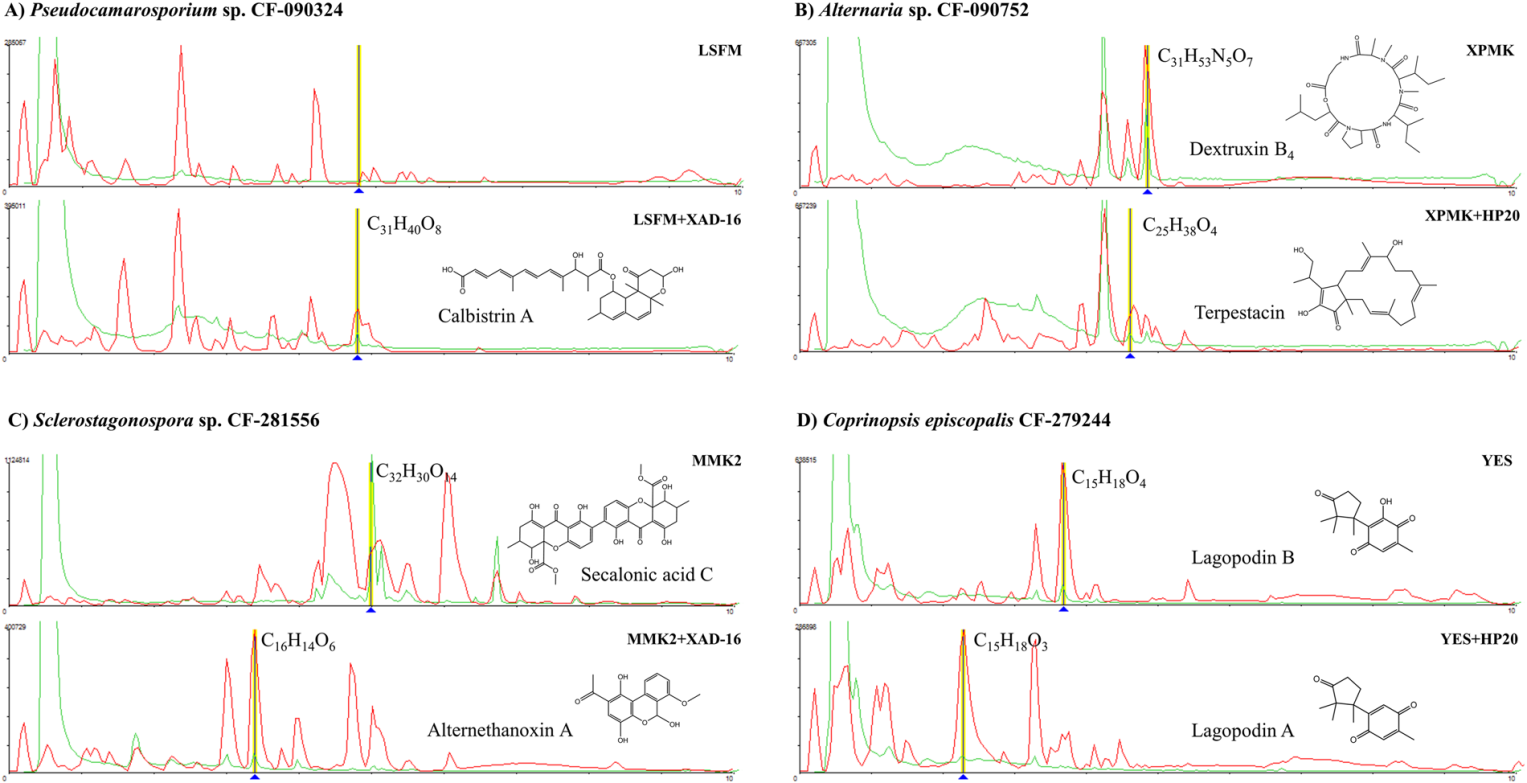
(**A**–**D**) Examples of the differential expression of antifungal agents induced by the presence of resins during the fermentation. Comparison of SMs profiles and identification of relevant components performed by LCMS.

Several examples of new activities obtained by the addition of resins during fermentation were due to known compounds identified by LCMS: (a) The broad-spectrum antifungal activity of the extracts of *Psudocamarosporium* sp. CF-090324 when grown in LSFM medium with XAD-16 resin could be explained by the presence of calbistrin A, a compound only produced in this condition (Fig. 4A); (b) Dextrusin B_4_, detected in the XPMK fermentation of *Alternaria* sp. CF-090752 explained the activity against A. *fumigatus* and HepG2. On the contrary, cultivation of the strain with HP-20 resin showed the induction of the antifungal agent terpestacin, extending the antifungal activity spectrum to *M*. *grisea* (Fig. 4B); (c) The production of mycotoxin secalonic acid C, identified in the extract of *Sclerostagonospora* sp. CF-281856, from a MMK2 fermentation explained the activity against *A*. *fumigatus*, *C*. *albicans* and *C*. *acutatum*. When this strain was cultivated in MMK2 with XAD-16, we observed an extended activity spectrum against *M*. *grisea*, due to the fact that the production of secalonic acid C was inhibited under these conditions and replaced by the production of a compound with molecular formula C_16_H_14_O_16_. This compound was dereplicated by the DNP database as the phytotoxic alternethanoxin A (UV matching) (Fig. 4C); Finally, (d) the sesquiterpenoid quinone lagopodin B was detected in the extract of the basidiomycete *Coprinopsis episcopalis* CF-279244 culture grown in YES medium. However, the addition of the polymeric HP-20 resin induced the production of lagopodin A, and only trace amounts of lagopodin B. This extract only showed activity against *M*. *grisea* when produced in the presence of resin (Fig. 4D).

### Differential expression of antitumoral activities

**Figure 5:**
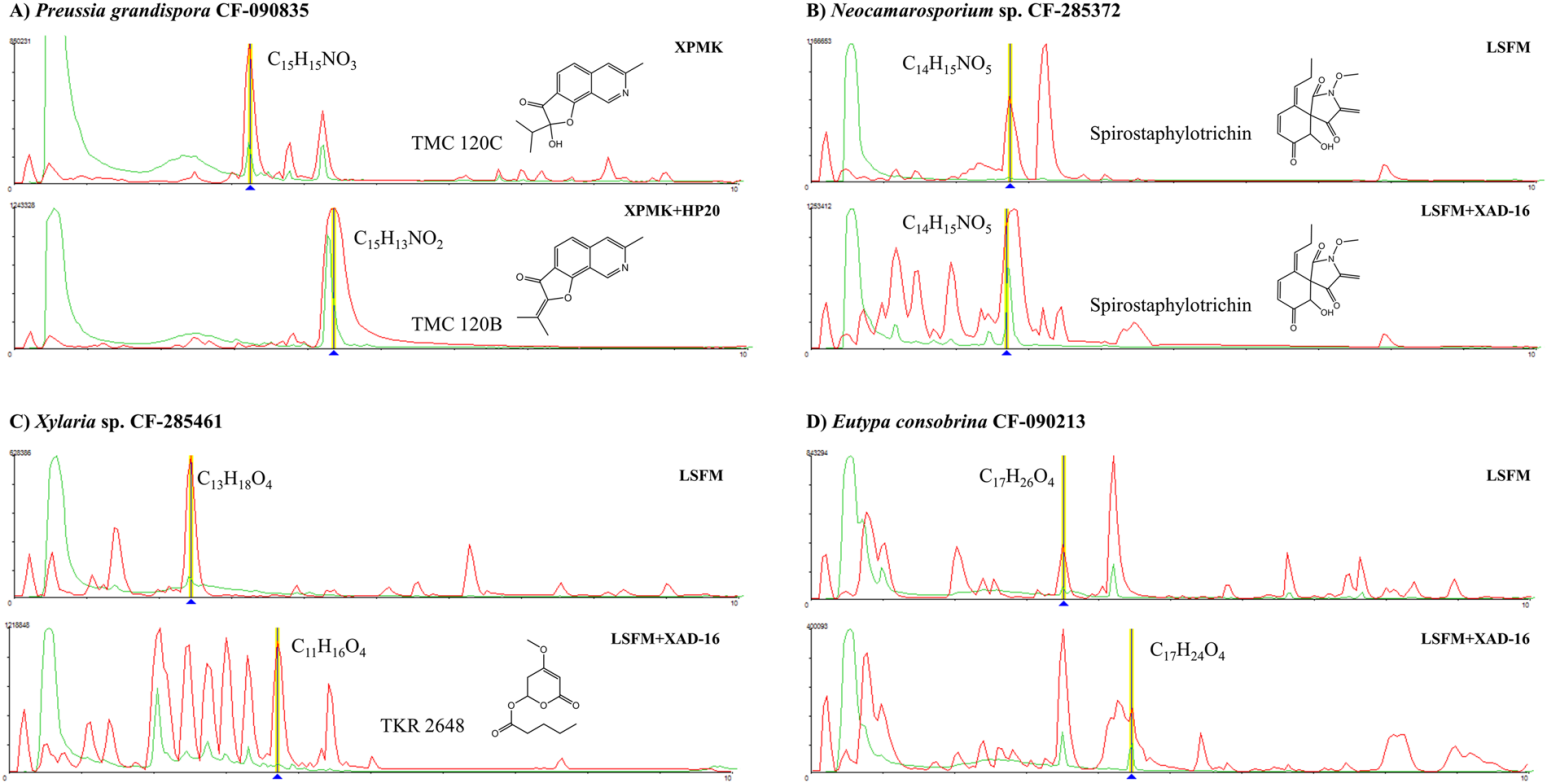
(**A**–**D**) Examples of the differential expression of cytotoxic agents induced by the presence of resins during fermentation. Comparison of SMs profiles and identification of relevant components performed by LCMS.

Similarly, eleven isolates presented inhibitory activities against the HepG2 cell line only when fermented in the presence of resins. In all cases, the presence of differential compounds in the secondary metabolite profiles was relevant when comparing fermentation conditions with and without resins. Some examples include: (a) *Preussia grandispora* CF-090835 that produced TMC-120C when grown in XPMK medium, whereas in presence of HP-20 resin, the fungus produced the related active compound TMC-120B with no trace of TMC-120C detected (Fig. 5A); (b) The semipreparative fractionation of the crude extract of *Neocamarosporium* sp. CF-285372 in LSFM with XAD-16 showed the accumulation of one mycotoxin of the spirostaphylotrichin family (C, D, G or H)^30^ (Fig. 5B), only present in small quantities in the inactive LSFM condition; (c) Five induced metabolites were detected in the HepG2 active extract from the strain *Xylaria* sp. CF-285461 when grown with XAD-16 resin. Bio-assay guided semipreparative HPLC fractionation confirmed the production of five secondary metabolites not present in the condition without resin: the antibiotic TKR 2648^31^, the α-pyrones 6-(1-hydroxypentyl)-4-methoxy-pyran-2-one and 6-pentyl-4-methoxy-pyran-2-one^32^ and the mellein derivates (R)-(-)-5-carboxymellein^33^ and (3 R)-5-cormylmellein^34^ (Fig. 5C); (d) *Eutypa consobrina* CF-090213, that showed different LC/MS profiles when grown in the presence of resin, was selected for further analyses and a follow-up chemical fractionation (Fig. 5D).

### New bioactive compounds induced in the presence of resins

**Figure 6:**
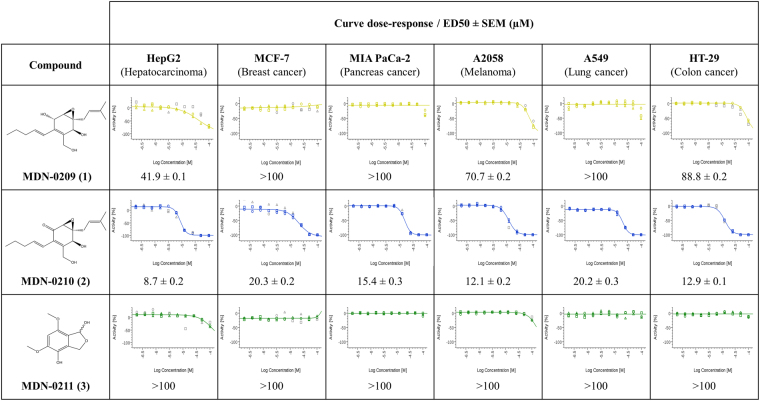
Dose-response curves and ED50 (µM) for new compounds **1–3** against a diverse panel of human cancer cells lines.

For some of the fungal isolates, the addition of polymeric resins induced antifungal or antitumoral activities that correlated with the production of unknown chemical diversity^11^. To prove this concept, we selected the strain that produced the most potent activity against both human pathogens and that potentially showed new chemical diversity for scale-up isolation and identification of potential new bioactive compounds. As a result, a 2-liter scale-up fermentation of the *Xylariales* strain *Eutypa consobrina* CF-090213 was prepared in LSFM medium containing XAD-16 resin for the purification of its active components. After bioassay guided fractionation of the CF-090213 extract, three active compounds (**1–3**, Fig. 6) were identified from this fungal isolate, a compound with molecular formula C_17_H_26_O_4_ (**1**; **MDN-0209**), produced both with and without the resin addition, and C_17_H_24_O_4_ (**2**; **MDN-0210**) and C_10_H_12_O_5_ (**3**; **MDN-0211**), induced only in presence of the XAD-16 resin. All three compounds were purified in amounts of 27, 2.4 and 1.4 mg, respectively. NMR analyses confirmed that these compounds were new secondary metabolites not yet described in nature (see Supplementary Information).

Further characterization of the antitumoral properties of these three compounds was carried out, testing them for cell proliferation inhibition against a panel of six human cancer cell lines, including hepatocarcinoma (HepG2), breast adenocarcinoma (MCF-7), pancreas cancer (Miapaca-2), melanoma (A-2058), lung cancer (A-549) and colon cancer (HT-29). Dose-response curves were performed at concentrations ranging from 0.01 to 100 µM while cells were treated for 72 h (see supplementary information) and ED_50_ (µM) was determined (Fig. 6). The most potent compound, **2**, presented a ED_50_ for HepG2 of 8.7 µM, and similar inhibition values for A-2058, HT-29 and Miapaca-2 cell lines, while for MCF-7 and A549 this compound was half as effective. Compound **1**, structurally related to **2**, showed a similar activity pattern, but less potent. On the contrary, compound **3** was inactive with an effective ED_50_ above 100 µM for the HepG2 cell line. None of the three purified compounds displayed any antifungal activities.

## Discussion

Few studies have reported the characterization of fungal endophytes in halophytic plants and generally, a low number of host species have been studied^35,36^. This report represents an exhaustive effort to extend the study to a large number of plant specimens from these biodiverse natural reserves, in order to isolate the culturable fungal endophyte community in halophytic and xerophytic plants. The plants included in this study are a representative example of the high diversity of species with restricted distribution due to the fact that they have adapted to these extreme environments, such as *Centaurea dracunculifolia*, *Euzomodendron bourgeanum*, *Helianthemum almeriense*, *Limonium majus* or *Moricandia foetida*. The wide community of plants studied has permitted to show that plants collected in arid zones of Andalusia are rich sources of fungal biodiversity with as many as 349 fungal isolates distributed among 19 taxonomic orders.

*Pleosporales* are the most frequent taxa identified among endophytes^37^. In line with this observation, in the present work *Pleosporales* was the most isolated order in the collected plants. Being the most abundant *Ascochyta*, *Phoma*, and the two recently described genera *Neomicrosphaeropsis*^23^ and *Neocamarosporium*^38^. *Neocamarosporium* was the most frequently isolated genus, mostly from halophytes including the important plant endemisms *Salsola papillosa* or *Sal*s*ola genistoides*. To date, various species of this genus have been described from saline soil or halophytic plants^24,39,40^. Their presence in hypersaline environments and the ability of our isolates to grow in a medium containing more than 3% of NaCl suggest that this genus is naturally halotolerant coinciding with a recent study^39^ (see Supplementary Information Fig. S2). The second most frequent genus was *Preussia*, confirming previous studies from plants collected in the Arizona desert^17^. Recently, our group has characterized and discussed the biodiversity and chemotaxonomy of these *Preussia* isolates^20^. *Alternaria* was the third genus present in the plants collected, in agreement with previous studies^41^. This genus features cosmopolitan fungi that include saprobic, endophytic and pathogenic species associated with a wide variety of substrates^42^. In addition, we have found several taxa from different plant species to those initially described, e.g. The genera *Neomicrosphaeropsis* and *Tamaricicola*, both reported as saprobic or weak pathogens of *Tamarix* species^23^, were also isolated from *Frankenia*, *Arthrocnemum*, *Lycium* and *Limonium* (see Supplementary Information Table S1).

The *Pleosporales* phylogenetic analyses showed several clades containing isolates that could represent undescribed species, such as, *Tamaricicola* sp. CF-288959 isolated from the endemic *Limonium majus*, and *Phoma* sp. CF-288917, *Neocamarosporium* sp. CF-288932, *Xenodydimella* sp. CF-090345 or *Camarosporium* sp. CF-090314. Although the phylogenetic analysis in this study was focused mainly on *Pleosporales*, we also found potentially undescribed species among the isolates of *Dothideales*, namely belonging to *Aureobasidium*, *Kabatiella* or *Selenophoma* genera. A new family of hormonemate derivatives with cytotoxic activity has been recently purified from the culture of *Dothiora* sp. CF-285353^43^, confirming the ability of this fungal community to produce new bioactive metabolites. Further studies to describe their morphologies and, in some cases, phylogenetic analyses based on housekeeping genes, should be carried out to confirm the taxonomic position of the new members of these orders.

The antifungal hit rates observed for our isolates were within the range of values reported for other studies with endophytic strains from Mediterranean areas^41,44^. Regarding the cytotoxic hit rates (19.2%), no previous data was available related with other antitumoral screenings performed with endophytes from arid areas. Our strains showed higher hit rates than endophytes reported from other environments^18,19,45^. Additionally, our isolates were able to produce a diversity of bioactive compounds from several chemical classes such as sesquiterpenoids (e.g. avocettin, trichothecene, lagopodins), polyketides (e.g. calbistrin A, phomasetin, altersetin), peptides (e.g. cyclo(phenyalanyl-prolyl)) and peptide polyketides (e.g. pleofungin A, naematolin).

It has been reported that the positive effects of resin additions to fermentation media are due to the adsorption of non-stable products or to the removal of a product involved in secondary metabolite pathway feedback repression^46^. The addition of polymeric resins to culture media during fungal fermentations induced and/or increased the production of bioactive molecules that were detected in all the assays tested in our screening (Fig. 2). A good example of resin induced antifungal activity was the generation of calbistrin A by the *Pseudocamarosporium* sp. CF-090324. This antifungal compound was previously isolated from various *Penicillium* and *Aspergillus* species^47,48^. Recently, the deletion of a putative polyketide synthase (PKS) in *Aspergillus aculeatus* and *Penicillium decumbens* has linked its biosynthesis to this gene cluster^49^. A homologous PKS is herein hypothesized to be cryptic in standard fermentation conditions, being this gene cluster expression activated in the presence of the resin. In addition, another five different compounds were isolated from *Xylaria* sp. CF-285461 in presence of XAD-16 resin: TKR2648, an inhibitor of metastasis of EL-4 and B16 tumor cells^31^, two α-pyrones, and two mellein derivatives. Two different PKS biosynthetic pathways have been described for the biosynthesis of mellein and α-pyrones in fungi^50,51^. In our case the addition of the resin could be indirectly modulating gene expression in this fungus in more than one pathway.

Other interesting cases were those in which the resin captured the bioactive compound and protected it against post-biosynthetic degradation or biotransformation as in the case of *Coprinopsis episcopalis* CF-279244 and lagopodin A. Lagopodin A is an unstable compound in aqueous solution is transformed into lagopodin B in neutral or slightly alkaline solutions^52^. The addition of resin during the fermentation of *C*. *episcopalis* avoided the transformation from form A to B. Other examples are the isoquinoline alkaloids TMC-120A, B and C, described as bioactive compounds produced by *Aspergillus ustus*. Previous time-course studies with this strain showed that TMC-120B is produced in early stages of fermentation and that compounds A and C are produced sequentially, suggesting that TMC-120A and C are derived from TMC-120B by biological or chemical transformation^53^. In our producer isolate, *Preussia grandispora* CF-090385, the resin captured the bioactive TMC-120B preventing its posterior transformation. On the other hand, Spirostaphylotrichins and related compounds have been reported as metabolites produced by different *Pleosporales*^54–56^. These compounds belong to a chemical class of bioactive metabolites which includes curvupallides and phaeosphaerides, which share the same precursor and therefore the same biosynthetic pathway^57^. This type of gene clusters may be widespread among *Pleosporales*. In our case the use of resin captured spirostaphylotrichin C during the fermentation of *Neocamarosporium* sp. CF-285372, being this the first report in this genus.

Herein we hypothesized and confirmed that the addition of adsorptive resins could promote displacement of secondary metabolites in solution during fungal fermentations with the potential induction of new secondary metabolites. When applied in an extensive prospection of endophytic fungi from arid areas, resins promoted the production of three new compounds **1–3** isolated from *Eutypa consobrina* CF-090213. *Eutypa* spp are frequently found as phytopathogens of grape crops^58^. To date, 18 secondary metabolites have been reported from *Eutypa* species (DNP), including siccayne, also known to have antimicrobial activity^59^. A number of related epoxycyclohexenone-based natural products similar to **1** and **2** have been reported^60^. Some of these products are known to be phytotoxic^61,62^, hence compounds **1–2** could be related to the phytopathogenic activity of *Eutypa* strains. More studies are necessary to understand the role of epoxycyclohexenones as virulence factors associated to the symptoms of *Eutypa* dieback.

This is the first study involving an extensive number of halophytic and xerophytic plant specimens surveyed from arid zones of southern Europe to culture and characterize their fungal symbiont community. We observed the dominant presence of members of *Pleosporales* in this fungal community along host plant species and sites, as well as host-specific fungal symbionts. This contributes to the understanding of ecological affiliations of fungal symbionts at regional and continental scales, where more studies are necessary to unravel the roles of these microorganisms in this plant community. Furthermore, the combination of the OSMAC approach, including the addition of adsorptive resins, with chemical diversity analyses, allowed us to highlight the high potential of these fungi as sources of bioactive secondary metabolites with biotechnological applications. As proof of concept, three new secondary metabolites were isolated and structurally elucidated from *Eutypa consobrina* when grown in the presence of resins. Further efforts on the isolation and structure elucidation of additional likely unknown bioactive secondary metabolites produced by 14 isolates of this fungal community are under way.

## Methods

### Plant collection

Representative arid areas of the Iberian southeast were surveyed, namely the provinces of Almeria and Granada, including Tabernas desert, Sierra de la Alhamilla, Cabo de Gata, Torre Garcia beach, Los Vados and the salt marshes of El Margen (Cullar). In these regions, the climatic conditions favor the accumulation of gypsum and other more soluble salts that benefit the development of endemic halophile or salt tolerant vegetation. A total of 63 characteristic plant species were collected from these arid areas (see Supplementary Information Table S4).

### Isolation Cultures and Characterization

Fungal endophytes were isolated using standard indirect isolation techniques^16^. Stems and leaves removed from each plant were cut into pieces of approximately 5 mm. These pieces were surface-disinfected by sequential washing with 95% ethanol (30 s), 25% commercial bleach (1 min) and 95% ethanol (30 s). Ten pieces of each plant sample were aseptically transferred to a Petri dish with corn meal agar (CMA) supplemented with streptomycin sulfate and oxytetracycline (50 mg/mL). Epiphyte fungi were also directly isolated from cleistothecia or conidiophores formed on plants by incubation in moist chambers. Isolates were cultured in YM agar (malt extract 10 g, yeast extract 2 g, agar 20 g, 1000 mL distilled H_2_O), to study their macroscopic and microscopic characteristics. Strains, designated with unique IDs (e.g., CF-285353), were preserved as frozen conidia and mycelia in 10% glycerol at −80 °C and are maintained in Fundación MEDINA’s fungal culture collection. DNA extraction, PCR amplification and DNA sequencing were performed as previously described^20^. Sequences of the complete ITS_1_-5.8S-ITS_2_-28S region or independent ITS and partial 28S rDNA sequences were compared with sequences at GenBank^®^, the NITE Biological Resource Center (http://www.nbrc.nite.go.jp) and CBS strain database (http://www.westerdijkinstitute.nl) by using the BLAST^®^ application.

### Phylogenetic analysis

Species and genus affinities of *Pleosporales* were inferred from a Bayesian analysis using the Markov Chain Monte Carlo (MCMC) approach with MrBayes 3.01^63^. To improve mixing of the chains, four incrementally heated simultaneous Monte Carlo Markov chains were run over 2 × 10^6^ generations. Hierarchical likelihood ratio tests with the MrModeltest® 2.2 software^64^ were used to calculate the Akaike Information Criterion (AIC) of the nucleotide substitution models. The model selected by AIC for the alignment was GTR + I + G that is based on six classes of substitution types, a portion of invariant alignment positions and mean substitution rates, varied across the remaining positions according to a gamma distribution. The MCMC processes were followed by a Dirichlet process prior (DPP) to obtain the substitution rates and nucleotide frequencies, and a unification of the rate parameter for the gamma distribution. The MCMC analysis was performed using a sampling frequency parameter of 100 and the first 1.000 trees were discarded before the majority rule consensus tree was calculated. In addition, Maximum Likelihood method (ML) and ultrafast bootstrap support values for phylogenetic trees were assessed calculating 1000 replicates with IQ-TREE software^65^. All parameters were estimated with this software (TPM2u + F + I + G4 nucleotide substitution model was selected), assuming a shape parameter of the Invar + Gamma distributed substitution rates (gamma shape alpha = 0.5355) to accommodate rate variations among sites and an estimation of nucleotide frequencies as A = 0.25, C = 0.23, G = 0.26 and T = 0.26.

### Generation of fungal extracts

The 349 fungal isolates were grown in four culture media with different carbon and nitrogen sources (LSFM, MMK2, XPMK and YES media) with and without the two adsorptive polymeric resins that presented best results per media in previous studies^11^. XAD-16N (Amberlite^®^ from Sigma-Aldrich^TM^) was added in LSFM and MMK2 media, whereas Diaion^®^ HP-20 (styrene-divinylbenzene Supelco^TM^) was added in XPMK and YES media. After 14 days of incubation fermentation broths were extracted according to the procedure previously described^11^.

### Extract bioactivity characterization

The evaluation of antifungal activities against phytopathogenic fungi was performed using two important plant pathogens (*Colletotrichum acutatum* CF-137177 and *Magnaporthe grisea* CF-105765). The microorganisms were incubated with the extracts in an agar-based assay for 24 h at 25 °C and the activities were scored by using an image analyzer to measure the diameter of inhibition halos^66^. Fungal extracts were also evaluated against two human pathogens: *Candida albicans* MY1055 and *Aspergillus fumigatus* ATCC 46645. Target microorganisms were incubated with the extracts in a liquid-based assay for 18–30 h at 37 °C and activities were scored using resazurin, an oxidation-reduction indicator of cell viability^67^. The cytotoxicity of the different extracts against the HepG2 cell line (hepatocellular carcinoma, ATCC HB 8065) was evaluated by the MTT reduction colorimetric assay, with the same incubation times and assay concentrations as those used for their antifungal evaluation^68,69^. Furthermore, for the characterization of the antitumoral activity profiles of purified compounds **1**–**3**, MTT assays were also performed against the MCF-7 (breast adenocarcinoma ATCC HTB-22), MIA PaCa-2 (pancreas carcinoma ATCC CRL 1420), A2058 (melanoma ATCC CLR-11147), A549 (lung carcinoma ATCC CCL-185) and HT-29 (colon adenocarcinoma ATCC HTB-38) cell lines and ED50 values were determined for each cell line^69^.

### Dereplication of bioactive extracts

Chemical profiling of extracts was performed by LC/MS and compared with our internal proprietary databases for the identification of known secondary metabolites by low resolution LC-LRMS (UV signal, retention time, and fragmentation patterns) against 970 standards, and high resolution LC-HRMS (retention time and accurate mass) against 1073 standards^70^. In addition, compounds that were not identified in the databases of standards were enriched by semi-preparative HPLC and, once detected by LC-HRMS, their predicted molecular formulas were searched against the Chapman & Hall Dictionary of Natural Products (DNP; v25.1) and confirmed by LC-ESI-HRMS/MS fragmentation in order to determine if they matched other compounds previously described in literature.

### Isolation of induced bioactive compounds

Bioactive fungal extracts were selected for 2 L scale-up fermentation in flasks containing 100 mL of LSFM medium with Amberlite XAD-16 resin (3% v/v) and extraction with acetone (2 L) under continuous shaking at 220 rpm for 1 h. The mycelium was then pelleted by centrifugation and the supernatant (4 L) was concentrated to 1.8 L under a stream of nitrogen. This solution was loaded (with continuous 1:1 water dilution, discarding the flow-through) on a column packed with SP-207SS reversed phase resin (brominated styren polymer, 65 g, 35 × 120 mm) previously equilibrated with water. The loaded column was further washed with water (2 L) and afterwards eluted at 8 mL min^−1^ on an automated flash-chromatography system (CombiFlash Rf^®^, Teledyne VERTEX^TM^) using a linear gradient from 10% to 100% acetone in water for 30 min and a final 100% acetone step for 15 min. 20 mL fractions were collected. DMSO (700 µL) was added to each fraction to avoid precipitation of molecules while samples were concentrated to evaporate acetone and water in a centrifugal vacuum evaporator. Fractions containing the bioactive compounds were characterized by LC-UV-MS for dereplication and identification of the compounds of interest. Preparative reverse phase HPLC fractionation (Agilent^TM^ Zorbax^®^ SB-C8, 22 × 250 mm, 7 μm; 20 mL min^−1^, UV detection at 210 nm) was performed with a linear gradient of acetonitrile in water from 5% to 100% over 37 min, and enriched fractions with compounds **1–3** were obtained at retention times of 27.7, 28.8 and 26.6 min, respectively. Subsequent preparative fractionation with a linear gradient from 6% to 10% of acetonitrile in water with 0.1% of TFA yielded 1.4 mg of **3** with 90% purity after 26 minutes; semipreparative reversed phase HPLC fractionation (Waters^TM^ XBridge^®^ C18, 10 × 150 mm, 5 μm; 3.6 mL min^−1^, UV detection at 210 nm) with 25.5% of acetonitrile in water was required to obtain pure compound **1** (27 mg) after 27 minutes of retention time. Finally, semipreparative reverse-phase HPLC fractionation (Agilent^TM^ Zorbax^®^ SB-C8, 9.4 × 250 mm, 5 μm; 3.6 mL min^−1^, UV detection at 210 nm) was required to obtain compound **2** (2.4 mg), at a retention time of 27 minutes in a 37.5% isocratic run. HRMS, NMR spectroscopy and Mosher analysis were employed in the structure elucidation of all three of the compounds (detailed in Supplementary Information).

## Electronic supplementary material

Supplementary information
