# Supplementary material for: Fungal endophytes from arid areas of Andalusia: high potential sources for antifungal and antitumoral agents
Source: Sci Rep. 2018 Jun 27;8:9729. doi: 10.1038/s41598-018-28192-5 (PMC6021435; doi:10.1038/s41598-018-28192-5)
Supplement: Supplementary file 1 — Supplementary information [file 41598_2018_28192_MOESM1_ESM.pdf]

Fungal endophytes from arid areas of Andalusia: high potential sources for antifungal and antitumoral agents

Victor González-Menéndez[1]*, Gloria Crespo[1], Nuria de Pedro[1], Caridad Diaz[1], Jesús Martín[1], Rachel Serrano[1], Thomas A. Mackenzie[1], Carlos Justicia[1], M. Reyes González-Tejero[2], M. Casares[2], Francisca Vicente[1], Fernando Reyes[1], José R. Tormo[1] and Olga Genilloud[1].

[1] Fundación MEDINA, Avda. del conocimiento 34, 18016 Granada, SPAIN
[2] Departamento de Botánica, Facultad de Farmacia, Universidad de Granada, C/ Prof. Clavera, s/n, 18011, Granada, SPAIN
*victor.gonzalez@medinaandalucia.es

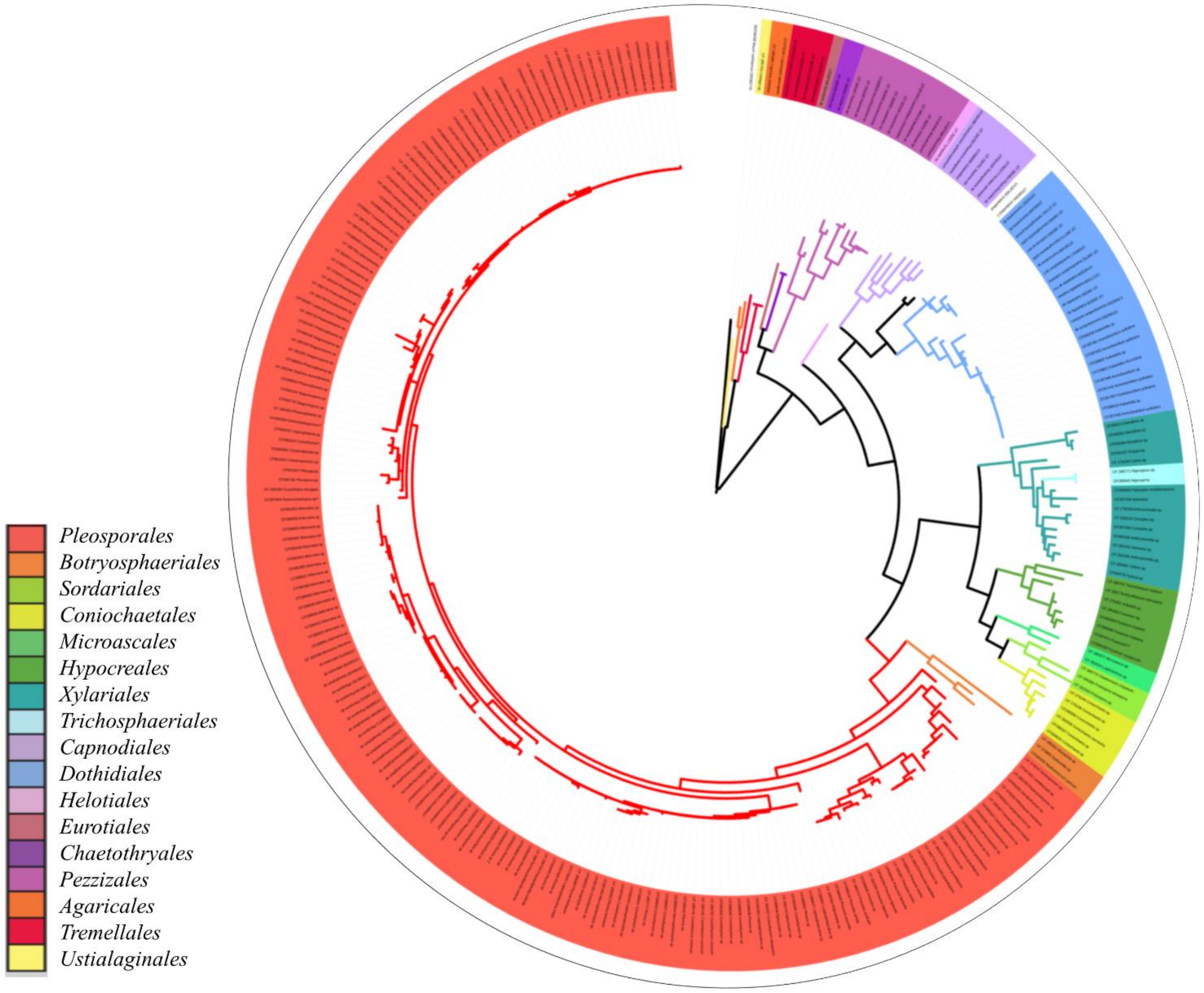

Supplementary Figure 1. Neighbor-joining tree based on nucleotide sequences of 28S rDNA gene of the 349 fungal isolates. Major taxonomic orders are labelled using differential color coding.

Supplementary Table 1. Total list of fungal isolates sort by genus.

Isolate	Genus	Species	Order	Substrate
CF-288903	*Alternaria*	*alternata*	*Pleosporales*	*Arthrocnemum macrostachyum*
CF-090318	*Alternaria*	*alternata*	*Pleosporales*	*Artemisia barrelieri*
CF-090322	*Alternaria*	*alternata*	*Pleosporales*	*Artemisia barrelieri*
CF-090366	*Alternaria*	*cumini*	*Pleosporales*	*Sedum sediforme*
CF-090246	*Alternaria*	*infectoria*	*Pleosporales*	*Genista umbellata*
CF-090261	*Alternaria*	*infectoria*	*Pleosporales*	*Asparagus horridus*
CF-282008	*Alternaria*	sp.	*Pleosporales*	*Retama sphaerocarpa*
CF-288921	*Alternaria*	sp.	*Pleosporales*	*Ononis tridentata*
CF-288922	*Alternaria*	sp.	*Pleosporales*	*Ononis tridentata*
CF-288909	*Alternaria*	sp.	*Pleosporales*	*Frankenia pulvirulenta*
CF-288960	*Alternaria*	sp.	*Pleosporales*	*Frankenia pulvirulenta*
CF-288961	*Alternaria*	sp.	*Pleosporales*	*Sonchus crassifolius*
CF-288939	*Alternaria*	sp.	*Pleosporales*	*Suaeda vera*
CF-288904	*Alternaria*	sp.	*Pleosporales*	*Arthrocnemum macrostachyum*
CF-090752	*Alternaria*	sp.	*Pleosporales*	*Thymelaea hirsuta*
CF-090365	*Alternaria*	sp.	*Pleosporales*	*Sedum sediforme*
CF-090395	*Alternaria*	sp.	*Pleosporales*	*Arthrocnemum macrostachyum*
CF-090254	*Alternaria*	sp.	*Pleosporales*	*Asparagus horridus*
CF-282338	*Anthostomella*	sp.	*Xylariales*	*Dittrichia viscosa*
CF-090350	*Anthostomella*	sp.	*Xylariales*	*Pinus halepenssis*
CF-279248	*Anthostomella*	sp.	*Xylariales*	*Retama sphaerocarpa*
CF-287454	*Ascochyta*	sp	*Pleosporales*	*Rosmarinus eriocalyx*
CF-287439	*Ascochyta*	sp.	*Pleosporales*	*Ononis fruticosa*
CF-282337	*Ascochyta*	sp.	*Pleosporales*	*Dittrichia viscosa*
CF-090376	*Ascochyta*	sp.	*Pleosporales*	*Ballota hirsuta*
CF-090372	*Ascochyta*	sp.	*Pleosporales*	*Sedum sediforme*
CF-285758	*Ascochyta*	sp.	*Pleosporales*	*Frankenia corymbosa*
CF-285355	*Ascochyta*	sp.	*Pleosporales*	*Asparagus horridus*
CF-285366	*Ascochyta*	sp.	*Pleosporales*	*Rosmarinus eriocalyx*
CF-287450	*Ascochyta*	sp.	*Pleosporales*	*Rosmarinus eriocalyx*
CF-287451	*Ascochyta*	sp.	*Pleosporales*	*Rosmarinus eriocalyx*
CF-090321	*Ascochyta*	sp.	*Pleosporales*	*Artemisia barrelieri*
CF-090240	*Ascochyta*	sp.	*Pleosporales*	*Genista umbellata*
CF-090840	*Ascochyta*	sp.	*Pleosporales*	*Launaea arborescens*
CF-091945	*Ascochyta*	sp.	*Pleosporales*	*Launaea arborescens*
CF-091946	*Ascochyta*	sp.	*Pleosporales*	*Lycium intricatum*
CF-090305	*Ascochyta*	sp.	*Pleosporales*	*Launaea arborescens*
CF-090252	*Ascochyta*	sp.	*Pleosporales*	*Genista umbellata*
CF-282009	*Ascorhizoctonia*	sp.	*Pezizales*	*Dittrichia viscosa*
CF-287465	*Aureobasidium*	*pullulans*	*Dothideales*	*Genista umbellata*
CF-287442	*Aureobasidium*	*pullulans*	*Dothideales*	*Ononis fruticosa*
CF-288910	*Aureobasidium*	*pullulans*	*Dothideales*	*Gypsophila tomentosa*
CF-090316	*Aureobasidium*	*pullulans*	*Dothideales*	*Artemisia barrelieri*
CF-285762	*Aureobasidium*	*pullulans subglaciale*	*Dothideales*	*Limbarda crithmoides*
CF-287440	*Aureobasidium*	sp.	*Dothideales*	*Ononis fruticosa*
CF-090333	*Aureobasidium*	sp.	*Dothideales*	*Anthyllis cystisoides*
CF-090336	*Bactrodesmium*	sp.	*Incertae sedis*	*Anthyllis cystisoides*
CF-090812	*Bartalinia*	sp.	*Xylariales*	*Limonium insigne*
CF-285376	*Basifimbria*	sp.	*Xylariales*	*Launaea arborescens*
CF-288963	*Biscogniauxia*	*mediterranea*	*Xylariales*	*Tamarix canariensis*
CF-285367	*Boubovia*	sp.	*Pezizales*	*Anthyllis terniflora*
CF-279249	*Cainia*	sp.	*Xylariales*	*Retama sphaerocarpa*
CF-090379	*Camarosporium*	sp.	*Pleosporales*	*Ballota hirsuta*
CF-090387	*Camarosporium*	sp.	*Pleosporales*	*Arthrocnemum macrostachyum*
CF-285350	*Camarosporium*	sp.	*Pleosporales*	*Euzomodendron bourgeanum*
CF-090314	*Camarosporium*	sp.	*Pleosporales*	*Artemisia barrelieri*
CF-090242	*Camarosporium*	sp.	*Pleosporales*	*Genista umbellata*
CF-285770	*Chaetomium*	*crispatum*	*Sordariales*	*Macrosyringion longiflorum*

CF-287476	*Chaetomium*	sp.	*Sordariales*	*Centaurea dracunculifolia*
CF-286679	*Chaetomium*	sp.	*Sordariales*	*Zygophylum fabago*
CF-286682	*Chaetothyriales*	sp.	*Chaetothryriales*	*Thymelaea hirsuta*
CF-091931	*Chaetothyriales*	sp.	*Chaetothryriales*	*Tamarix canariensis*
CF-090214	*Coleophoma*	*cylindrospora*	*Pleosporales*	*Ziziphus lotus*
CF-282003	*Comoclathris*	sp.	*Pleosporales*	*Retama sphaerocarpa*
CF-091922	*Comoclathris*	sp.	*Pleosporales*	*Ziziphus lotus*
CF-090361	*Comoclathris*	sp.	*Pleosporales*	*Sedum sediforme*
CF-285379	*Comoclathris*	sp.	*Pleosporales*	*Fagonia cretica*
CF-287447	*Comoclathris*	sp.	*Pleosporales*	*Rosmarinus eriocalyx*
CF-090301	*Comoclathris*	sp.	*Pleosporales*	*Launaea arborescens*
CF-091944	*Comoclathris*	sp.	*Pleosporales*	*Launaea arborescens*
CF-090266	*Comoclathris*	sp.	*Pleosporales*	*Lygeum spartum*
CF-090267	*Comoclathris*	sp.	*Pleosporales*	*Lygeum spartum*
CF-090792	*Comoclathris*	sp.	*Pleosporales*	*Lygeum spartum*
CF-091934	*Comoclathris*	sp.	*Pleosporales*	*Lygeum spartum*
CF-090763	*Comoclathris*	sp.	*Pleosporales*	*Nerium oleander*
CF-090766	*Comoclathris*	sp.	*Pleosporales*	*Nerium oleander*
CF-282006	*Coniochaeta*	*saccardoi*	*Coniochaetales*	*Retama sphaerocarpa*
CF-288927	*Coniochaeta*	sp.	*Coniochaetales*	*Ononis tridentata*
CF-288956	*Coniochaeta*	sp.	*Coniochaetales*	*Thymus zygis subs gracilis*
CF-279239	*Coniochaeta*	sp.	*Coniochaetales*	*Retama sphaerocarpa*
CF-091953	*Coniothyrium*	sp.	*Pleosporales*	*Anthyllis cystisoides*
CF-090330	*Coniothyrium*	sp.	*Pleosporales*	*Cistus albidus*
CF-090362	*Coniothyrium*	sp.	*Pleosporales*	*Sedum sediforme*
CF-285356	*Coniothyrium*	sp.	*Pleosporales*	*Asparagus horridus*
CF-090243	*Coniothyrium*	sp.	*Pleosporales*	*Genista umbellata*
CF-090236	*Constantinomyces*	sp.	*Capnodiales*	*Suaeda vera*
CF-279244	*Coprinopsis*	*episcopalis*	*Agaricales*	*Retama sphaerocarpa*
CF-285465	*Coprinus*	*cinereus*	*Agaricales*	*Salsola genistoides*
CF-285748	*Filobasidium*	*chernovii*	*Tremellales*	*Beta macrocarpa*
CF-285760	*Cryptococcus*	sp.	*Tremellales*	*Genista umbellata*
CF-285750	*Cryptococcus*	sp.	*Tremellales*	*Centaurea dracunculifolia*
CF-285752	*Cryptococcus*	sp.	*Tremellales*	*Centaurea dracunculifolia*
CF-285749	*Paradendryphiella*	*salina*	*Pleosporales*	*Centaurea dracunculifolia*
CF-091936	*Alternaria*	*penicillata*	*Pleosporales*	*Limonium insigne*
CF-285362	*Dendryphion*	sp.	*Pleosporales*	*Asparagus horridus*
CF-288958	*Devriesia*	sp.	*Capnodiales*	*Whitania frutescens*
CF-091918	*Devriesia*	sp.	*Capnodiales*	*Thymelaea hirsuta*
CF-091923	*Devriesia*	sp.	*Capnodiales*	*Nerium oleander*
CF-090344	*Diaporthe*	sp	*Diaporthales*	*Onoris ramosissina*
CF-279247	*Dictyosporium*	sp.	*Pleosporales*	*Retama sphaerocarpa*
CF-288938	*Didymella*	sp.	*Pleosporales*	*Sonchus crassifolius*
CF-090355	*Didymella*	sp.	*Pleosporales*	*Sedum sediforme*
CF-288950	*Didymocytris*	sp.	*Pleosporales*	*Teucrium capitatum sbsp. gracillium*
CF-090768	*Dimorphosporicola*	sp.	*Pleosporales*	*Suaeda vera*
CF-091943	*Dimorphosporicola*	sp.	*Pleosporales*	*Suaeda vera*
CF-090383	*Dimorphosporicola*	*traganii*	*Pleosporales*	*Arthrocnemum macrostachyum*
CF-090749	*Diplodia*	sp.	*Botryosphaeriales*	*Thymelaea hirsuta*
CF-285354	*Dothiora*	sp.	*Dothideales*	*Moricandia foetida*
CF-285353	*Dothiora*	sp.	*Dothideales*	*Launaea arborescens*
CF-285777	*Dothiorella*	sp.	*Botryosphaeriales*	*Teucrium capitatum sbsp. gracillium*
CF-278800	*Dothiorella*	sp.	*Botryosphaeriales*	*Dittrichia viscosa*
CF-285759	*Entodesmium*	sp.	*Pleosporales*	*Genista umbellata*
CF-288946	*Epicoccum*	*nigrum*	*Pleosporales*	*Teucrium capitatum sbsp. gracillium*
CF-285743	*Epicoccum*	sp.	*Pleosporales*	*Atriplex glauca*
CF-287460	*Eurotium*	sp.	*Eurotiales*	*Launaea arborescens*
CF-090213	*Eutypa*	*consobrina*	*Xylariales*	*Ziziphus lotus*
CF-090257	*Eutypa*	sp.	*Xylariales*	*Asparagus horridus*
CF-090782	*Foliophoma*	sp.	*Pleosporales*	*Asparagus horridus*
CF-090384	*Fusarium*	*equiseti*	*Hypocreales*	*Arthrocnemum macrostachyum*

CF-285462	*Fusarium*	*equiseti*	*Hypocreales*	*Salsola genistoides*
CF-288908	*Fusarium*	*redolens*	*Hypocreales*	*Frankenia pulvirulenta*
CF-285764	*Fusarium*	sp.	*Hypocreales*	*Limonium insigne*
CF-287474	*Fusarium*	sp.	*Hypocreales*	*Asparagus horridus*
CF-090256	*Fusarium*	sp.	*Hypocreales*	*Asparagus horridus*
CF-090320	*Hazslinszkyomyces*	sp.	*Pleosporales*	*Artemisia barrelieri*
CF-282335	*Homortomyces*	*tamaricis*	*Incertae sedis*	*Dittrichia viscosa*
CF-090351	*Hormonema*	*carpetanum*	*Dothideales*	*Sedum sediforme*
CF-091927	*Hormonema*	sp.	*Dothideales*	*Genista umbellata*
CF-091949	*Hormonema*	sp.	*Dothideales*	*Rhamnus lycioides*
CF-092177	*Hortaea*	*werneckii*	*Capnodiales*	*Arthrocnemum macrostachyum*
CF-090396	*Hortaea*	*werneckii*	*Capnodiales*	*Chamaerops humilis*
CF-288947	*Kabatiella*	sp.	*Dothideales*	*Teucrium capitatum sbsp. gracillium*
CF-285359	*Kabatiella*	sp.	*Dothideales*	*Asparagus horridus*
CF-091935	*Kabatiella*	sp.	*Dothideales*	*Limonium insigne*
CF-090299	*Kabatiella*	sp.	*Dothideales*	*Launaea arborescens*
CF-090814	*Kabatiella*	sp.	*Dothideales*	*Limonium insigne*
CF-091181	*Kalmusia*	sp.	*Pleosporales*	*Asparagus horridus*
CF-287452	*Keissieriella*	sp.	*Pleosporales*	*Rosmarinus eriocalyx*
CF-285360	*Knufia*	sp.	*Incertae sedis*	*Asparagus horridus*
CF-285361	*Knufia*	sp.	*Incertae sedis*	*Asparagus horridus*
CF-285464	*Lapidomyces*	sp.	*Capnodiales*	*Fagonia cretica*
CF-090797	*Lapidomyces*	sp.	*Capnodiales*	*Lygeum spartum*
CF-287444	*Lasiobolidium*	*orbiculoides*	*Pezizales*	*Otanthus maritimus*
CF-279246	*Lecythophora*	sp.	*Coniochaetales*	*Retama sphaerocarpa*
CF-090357	*Leptosphaeria*	*hispanica*	*Pleosporales*	*Sedum sediforme*
CF-279243	*Leptospora*	sp.	*Incertae sedis*	*Retama sphaerocarpa*
CF-090382	*Libertasomyces*	sp.	*Pleosporales*	*Ballota hirsuta*
CF-092175	*Libertasomyces*	sp.	*Pleosporales*	*Ballota hirsuta*
CF-092176	*Libertasomyces*	sp.	*Pleosporales*	*Ballota hirsuta*
CF-090297	*Lophiostoma*	sp	*Pleosporales*	*Thymus hyemalis*
CF-090828	*Lophiostoma*	sp.	*Pleosporales*	*Thymus hyemalis*
CF-282004	*Lophotrichus*	sp.	*Microascales*	*Retama sphaerocarpa*
CF-285351	*Masarina*	sp.	*Botryosphaeriales*	*Ziziphus lotus*
CF-285371	*Microascus*	*trigonosporus*	*Microascales*	*Anabasis articulata*
CF-279241	*Coniothyrium*	*olivaceum*	*Pleosporales*	*Retama sphaerocarpa*
CF-090371	*Morinia*	*pestalozzioides*	*Amphisphaeriales*	*Sedum sediforme*
CF-090313	*Morinia*	sp.	*Xylariales*	*Artemisia barrelieri*
CF-285778	*Albifimbria*	*verrucaria*	*Hypocreales*	*Thymus hyemalis*
CF-282342	*Nemania*	sp.	*Xylariales*	*Dittrichia viscosa*
CF-285348	*Neocamarosporium*	*chicastinanum*	*Pleosporales*	*Salsola papollosa*
CF-285352	*Neocamarosporium*	sp.	*Pleosporales*	*Atriplex glauca*
CF-285744	*Neocamarosporium*	sp.	*Pleosporales*	*Atriplex glauca*
CF-285745	*Neocamarosporium*	sp.	*Pleosporales*	*Atriplex glauca*
CF-285746	*Neocamarosporium*	sp.	*Pleosporales*	*Atriplex glauca*
CF-285747	*Neocamarosporium*	sp.	*Pleosporales*	*Beta macrocarpa*
CF-288928	*Neocamarosporium*	sp.	*Pleosporales*	*Salsola vermiculata*
CF-288930	*Neocamarosporium*	sp.	*Pleosporales*	*Salsola vermiculata*
CF-288932	*Neocamarosporium*	sp.	*Pleosporales*	*Salsola vermiculata*
CF-288934	*Neocamarosporium*	sp.	*Pleosporales*	*Salsola vermiculata*
CF-285775	*Neocamarosporium*	sp.	*Pleosporales*	*Suaeda vera*
CF-288962	*Neocamarosporium*	sp.	*Pleosporales*	*Arthrocnemum macrostachyum*
CF-090335	*Neocamarosporium*	sp.	*Pleosporales*	*Anthyllis cystisoides*
CF-090390	*Neocamarosporium*	sp.	*Pleosporales*	*Arthrocnemum macrostachyum*
CF-090392	*Neocamarosporium*	sp.	*Pleosporales*	*Arthrocnemum macrostachyum*
CF-090393	*Neocamarosporium*	sp.	*Pleosporales*	*Arthrocnemum macrostachyum*
CF-090394	*Neocamarosporium*	sp.	*Pleosporales*	*Arthrocnemum macrostachyum*
CF-090399	*Neocamarosporium*	sp.	*Pleosporales*	*Chamaerops humilis*
CF-285349	*Neocamarosporium*	sp.	*Pleosporales*	*Anabasis articulata*
CF-285368	*Neocamarosporium*	sp.	*Pleosporales*	*Anabasis articulata*
CF-285372	*Neocamarosporium*	sp.	*Pleosporales*	*Anabasis articulata*

CF-285373	*Neocamarosporium*	sp.	*Pleosporales*	*Salsola papillosa*
CF-285374	*Neocamarosporium*	sp.	*Pleosporales*	*Salsola papillosa*
CF-285377	*Neocamarosporium*	sp.	*Pleosporales*	*Suaeda vera*
CF-090284	*Neocamarosporium*	sp.	*Pleosporales*	*Salicornia ramossissima*
CF-090228	*Neocamarosporium*	sp.	*Pleosporales*	*Suaeda vera*
CF-090759	*Neocamarosporium*	sp.	*Pleosporales*	*Nerium oleander*
CF-090841	*Neocamarosporium*	sp.	*Pleosporales*	*Salsola genistoides*
CF-090842	*Neocamarosporium*	sp.	*Pleosporales*	*Salsola genistoides*
CF-090843	*Neocamarosporium*	sp.	*Pleosporales*	*Salsola genistoides*
CF-090227	*Neocamarosporium*	sp.	*Pleosporales*	*Suaeda vera*
CF-090309	*Neocamarosporium*	sp.	*Pleosporales*	*Suaeda vera*
CF-090312	*Neocamarosporium*	sp.	*Pleosporales*	*Suaeda vera*
CF-090773	*Neocamarosporium*	sp.	*Pleosporales*	*Genista umbellata*
CF-090824	*Neocamarosporium*	sp.	*Pleosporales*	*Thymus hyemalis*
CF-285768	*Neocamarosporium*	sp.	*Pleosporales*	*Lycium intricatum*
CF-287455	*Neocamarosporium*	sp.	*Pleosporales*	*Lycium intricatum*
CF-286681	*Neodidymelliopsis*	sp.	*Pleosporales*	*Cynanchum acutum*
CF-090206	*Neofusicoccum*	*parvum*	*Botryosphaeriales*	*Ziziphus lotus*
CF-090380	*Neofusicoccum*	sp.	*Botryosphaeriales*	*Ballota hirsuta*
CF-285742	*Neomicrosphaeropsis*	sp.	*Pleosporales*	*Arthrocnemum macrostachyum*
CF-288941	*Neomicrosphaeropsis*	sp.	*Pleosporales*	*Tamarix canariensis*
CF-288942	*Neomicrosphaeropsis*	sp.	*Pleosporales*	*Tamarix canariensis*
CF-288943	*Neomicrosphaeropsis*	sp.	*Pleosporales*	*Tamarix canariensis*
CF-288907	*Neomicrosphaeropsis*	sp.	*Pleosporales*	*Frankenia pulvirulenta*
CF-285741	*Neomicrosphaeropsis*	sp.	*Pleosporales*	*Arthrocnemum macrostachyum*
CF-288902	*Neomicrosphaeropsis*	sp.	*Pleosporales*	*Arthrocnemum macrostachyum*
CF-090262	*Neomicrosphaeropsis*	sp.	*Pleosporales*	*Tamarix canariensis*
CF-090785	*Neomicrosphaeropsis*	sp.	*Pleosporales*	*Tamarix canariensis*
CF-285767	*Neomicrosphaeropsis*	sp.	*Pleosporales*	*Lycium intricatum*
CF-285780	*Neosetophoma*	sp.	*Pleosporales*	*Thymelaea hirsuta*
CF-288948	*Neosetophoma*	sp.	*Pleosporales*	*Teucrium capitatum sbsp. gracillium*
CF-282001	*Neosetophoma*	sp.	*Pleosporales*	*Retama sphaerocarpa*
CF-285771	*Nigrospora*	sp.	*Incertae sedis*	*Phragmites australis*
CF-288945	*Nigrospora*	sp.	*Incertae sedis*	*Tamarix canariensis*
CF-287449	*Orbicula*	sp.	*Pezizales*	*Rosmarinus eriocalyx*
CF-090248	*Paracamarosporium*	sp.	*Pleosporales*	*Genista umbellata*
CF-285765	*Paraconiothyrium*	sp.	*Pleosporales*	*Lygeum spartum*
CF-282005	*Pezzizales*	sp.	*Pezizales*	*Retama sphaerocarpa*
CF-282011	*Pezzizales*	sp.	*Pezizales*	*Dittrichia viscosa*
CF-282336	*Pezzizales*	sp.	*Pezizales*	*Dittrichia viscosa*
CF-285776	*Phaeosphaeria*	sp.	*Pleosporales*	*Teucrium capitatum sbsp. gracillium*
CF-285779	*Phaeosphaeria*	sp.	*Pleosporales*	*Thymus zygis subs gracilis*
CF-285363	*Phaeosphaeria*	sp.	*Pleosporales*	*Moricandia foetida*
CF-288952	*Phaeosphaeria*	sp.	*Pleosporales*	*Teucrium capitatum sbsp. gracillium*
CF-282010	*Phaeosphaeria*	sp.	*Pleosporales*	*Dittrichia viscosa*
CF-285358	*Phaeotheca*	*triangularis*	*Capnodiales*	*Asparagus horridus*
CF-278520	*Phoma*	sp.	*Pleosporales*	*Opuntia ficus-indica*
CF-288912	*Phoma*	sp.	*Pleosporales*	*Gypsophila tomentosa*
CF-288915	*Phoma*	sp.	*Pleosporales*	*Lepidium subulatum*
CF-288914	*Phoma*	sp.	*Pleosporales*	*Lepidium subulatum*
CF-288919	*Phoma*	sp.	*Pleosporales*	*Ononis tridentata*
CF-288917	*Phoma*	sp.	*Pleosporales*	*Lygeum spartum*
CF-288953	*Phoma*	sp.	*Pleosporales*	*Thymus zygis subs gracilis*
CF-092178	*Phoma*	sp.	*Pleosporales*	*Chamaerops humilis*
CF-285365	*Phoma*	sp.	*Pleosporales*	*Rosmarinus eriocalyx*
CF-091947	*Phoma*	sp.	*Pleosporales*	*Suaeda vera*
CF-090323	*Phoma*	sp.	*Pleosporales*	*Artemisia barrelieri*
CF-092164	*Phoma*	sp.	*Pleosporales*	*Lygeum spartum*
CF-090291	*Phoma*	sp.	*Pleosporales*	*Thymus hyemalis*
CF-090829	*Phoma*	sp.	*Pleosporales*	*Thymus hyemalis*
CF-090223	*Phomopsis*	sp.	*Diaporthales*	*Ziziphus lotus*

CF-285364	*Pleiochaeta*	sp.	*Incertae sedis*	*Moricandia foetida*
CF-288936	*Pleospora*	*herbarum*	*Pleosporales*	*Sonchus crassifolius*
CF-285761	*Pleospora*	*herbarum*	*Pleosporales*	*Limbarda crithmoides*
CF-090249	*Pleospora*	*herbarum*	*Pleosporales*	*Genista umbellata*
CF-090771	*Pleospora*	*herbarum*	*Pleosporales*	*Genista umbellata*
CF-090753	*Pleospora*	sp.	*Pleosporales*	*Ziziphus lotus*
CF-090826	*Pleospora*	sp.	*Pleosporales*	*Thymus hyemalis*
CF-091933	*Pleospora*	sp.	*Pleosporales*	*Lygeum spartum*
CF-282343	*Podospora*	*pleiospora*	*Sordariales*	*Dittrichia viscosa*
CF-285369	*Schizothecium*	*tetrasporum*	*Sordariales*	*Anabasis articulata*
CF-288933	*Preussia*	*australis*	*Pleosporales*	*Salsola vermiculata*
CF-285375	*Preussia*	*australis*	*Pleosporales*	*Launaea arborescens*
CF-091940	*Preussia*	*australis*	*Pleosporales*	*Thymus hyemalis*
CF-285772	*Preussia*	*grandispora*	*Pleosporales*	*Phragmites australis*
CF-285773	*Preussia*	*grandispora*	*Pleosporales*	*Phragmites australis*
CF-090838	*Preussia*	*grandispora*	*Pleosporales*	*Launaea arborescens*
CF-091941	*Preussia*	*grandispora*	*Pleosporales*	*Thymus hyemalis*
CF-282339	*Preussia*	*lignicola*	*Pleosporales*	*Dittrichia viscosa*
CF-090241	*Preussia*	*lignicola*	*Pleosporales*	*Genista umbellata*
CF-090293	*Preussia*	*lignicola*	*Pleosporales*	*Thymus hyemalis*
CF-285357	*Preussia*	*similis*	*Pleosporales*	*Asparagus horridus*
CF-288918	*Preussia*	sp.	*Pleosporales*	*Lygeum spartum*
CF-288931	*Preussia*	sp.	*Pleosporales*	*Salsola vermiculata*
CF-288906	*Preussia*	sp.	*Pleosporales*	*Arthrocnemum macrostachyum*
CF-279766	*Preussia*	sp.	*Pleosporales*	*Retama sphaerocarpa*
CF-287459	*Preussia*	sp.	*Pleosporales*	*Launaea arborescens*
CF-285370	*Preussia*	sp.	*Pleosporales*	*Anabasis articulata*
CF-285378	*Preussia*	sp.	*Pleosporales*	*Salsola oppositifolia*
CF-090835	*Preussia*	sp.	*Pleosporales*	*Thymus hyemalis*
CF-090338	*Pseudocamarosporium*	sp.	*Pleosporales*	*Anthyllis cystisoides*
CF-285347	*Pseudocamarosporium*	sp.	*Pleosporales*	*Helianthemum almeriense*
CF-090324	*Pseudocamarosporium*	sp.	*Pleosporales*	*Artemisia barrelieri*
CF-285753	*Pseudodiplodia*	*ruticola*	*Botryosphaeriales*	*Cynanchum acutum*
CF-285754	*Pseudodiplodia*	*ruticola*	*Botryosphaeriales*	*Cynanchum acutum*
CF-285755	*Pseudodiplodia*	*ruticola*	*Botryosphaeriales*	*Cynanchum acutum*
CF-285756	*Pseudodiplodia*	sp.	*Botryosphaeriales*	*Cynanchum acutum*
CF-279245	*Pseudopithomyces*	*chartarum*	*Pleosporales*	*Retama sphaerocarpa*
CF-287448	*Pseudoseptoria*	*obscura*	*Dothideales*	*Rosmarinus eriocalyx*
CF-281030	*Pulvinula*	sp.	*Pezizales*	*Retama sphaerocarpa*
CF-287464	*Pyrenochaeta*	sp.	*Pleosporales*	*Genista umbellata*
CF-090337	*Rhizoctonia*	sp.	*Cantharellales*	*Anthyllis cystisoides*
CF-287466	*Rosellinia*	sp.	*Xylariales*	*Artemisia barrelieri*
CF-285766	*Scclerostagonospora*	sp	*Pleosporales*	*Lygeum spartum*
CF-090364	*Seiridium*	sp.	*Amphisphaeriales*	*Sedum sediforme*
CF-285463	*Selenophoma*	*juncea*	*Dothideales*	*Salsola oppositifolia*
CF-285774	*Selenophoma*	*juncea*	*Dothideales*	*Salsola oppositifolia*
CF-277101	*Selenophoma*	sp.	*Dothideales*	*Spartium junceum*
CF-090317	*Selenophoma*	sp.	*Dothideales*	*Artemisia barrelieri*
CF-285346	*Septoria*	*arundinacea*	*Capnodiales*	*Phragmites australis*
CF-285751	*Septoria*	sp.	*Capnodiales*	*Centaurea dracunculifolia*
CF-090353	*Lepteutypa*	*cupressi*	*Xylariales*	*Sedum sediforme*
CF-287457	*Setomelanomma*	sp.	*Pleosporales*	*Lycium intricatum*
CF-288954	*Sordaria*	sp.	*Sordariales*	*Thymus zygis subs gracilis*
CF-090778	*Stagnospora*	sp	*Pleosporales*	*Asparagus horridus*
CF-281556	*Stagnospora*	sp.	*Pleosporales*	*Stipa tenacissima*
CF-090378	*Stagnospora*	sp.	*Pleosporales*	*Ballota hirsuta*
CF-090359	*Stagnospora*	sp.	*Pleosporales*	*Sedum sediforme*
CF-090235	*Stagnospora*	sp.	*Pleosporales*	*Suaeda vera*
CF-090258	*Stagnospora*	sp.	*Pleosporales*	*Asparagus horridus*
CF-090776	*Stemphylium*	sp	*Pleosporales*	*Genista umbellata*
CF-090346	*Stemphylium*	sp.	*Pleosporales*	*Onoris ramosissina*

CF-090850	*Stemphylium*	sp.	*Pleosporales*	*Onoris ramosissina*
CF-090781	*Stemphylium*	sp.	*Pleosporales*	*Asparagus horridus*
CF-090250	*Stemphylium*	sp.	*Pleosporales*	*Genista umbellata*
CF-090279	*Tamaricicola*	sp.	*Pleosporales*	*Limonium insigne*
CF-288916	*Tamaricicola*	sp.	*Pleosporales*	*Limonium majus*
CF-288959	*Tamaricicola*	sp.	*Pleosporales*	*Limonium majus*
CF-091926	*Teratosphaeria*	sp.	*Capnodiales*	*Genista umbellata*
CF-090304	*Teratosphaeria*	sp.	*Capnodiales*	*Launaea arborescens*
CF-287456	*Teratosphaeria*	sp.	*Capnodiales*	*Lycium intricatum*
CF-091928	*Toxicocladosporium*	sp.	*Capnodiales*	*Asparagus horridus*
CF-277739	*Trichothecium*	*roseum*	*Hypocreales*	*Retama sphaerocarpa*
CF-285757	*Trichothecium*	*roseum*	*Hypocreales*	*Dorycnium pentaphyllum*
CF-282017	*Tympanis*	sp.	*Helotiales*	*Juniperus oxycedrus*
CF-290943	*Unidentified*	Fungus	*not determined*	*Genista umbellata*
CF-290928	*Unidentified*	Fungus	*not determined*	*Limodorum abortivum*
CF-290929	*Unidentified*	Fungus	*not determined*	*Limodorum abortivum*
CF-290937	*Unidentified*	Fungus	*not determined*	*Retama sphaerocarpa*
CF-287441	*Unidentified*	Fungus	*not determined*	*Ononis fruticosa*
CF-288924	*Unidentified*	Fungus	*not determined*	*Ononis tridentata*
CF-288925	*Unidentified*	Fungus	*not determined*	*Ononis tridentata*
CF-287475	*Unidentified*	Fungus	*not determined*	*Centaurea dracunculifolia*
CF-090748	*Unidentified*	Fungus	*not determined*	*Thymelaea hirsuta*
CF-090210	*Unidentified*	Fungus	*not determined*	*Ziziphus lotus*
CF-090222	*Unidentified*	Fungus	*not determined*	*Ziziphus lotus*
CF-090758	*Unidentified*	Fungus	*not determined*	*Ziziphus lotus*
CF-282344	*Unidentified*	Fungus	*not determined*	*Dittrichia viscosa*
CF-092179	*Unidentified*	Fungus	*not determined*	*Nicotiana glauca*
CF-091954	*Unidentified*	Fungus	*not determined*	*Onoris ramosissina*
CF-091948	*Unidentified*	Fungus	*not determined*	*Cistus albidus*
CF-287472	*Unidentified*	Fungus	*not determined*	*Asparagus horridus*
CF-090273	*Unidentified*	Fungus	*not determined*	*Limonium insigne*
CF-091924	*Unidentified*	Fungus	*not determined*	*Nerium oleander*
CF-091929	*Unidentified*	Fungus	*not determined*	*Tamarix canariensis*
CF-285763	*Ustilago*	sp.	*Ustilaginales*	*Launaea arborescens*
CF-090221	*Verrucocladosporium*	sp.	*Capnodiales*	*Ziziphus lotus*
CF-279251	*Volutella*	sp.	*Hypocreales*	*Retama sphaerocarpa*
CF-288937	*Xenodidymella*	sp.	*Pleosporales*	*Sonchus crassifolius*
CF-288955	*Xenodidymella*	sp.	*Pleosporales*	*Thymus zygis subs gracilis*
CF-288957	*Xenodidymella*	sp.	*Pleosporales*	*Thymus zygis subs gracilis*
CF-090345	*Xenodidymella*	sp.	*Pleosporales*	*Onoris ramosissina*
CF-092173	*Xenodidymella*	sp.	*Pleosporales*	*Ballota hirsuta*
CF-290936	*Xylaria*	sp.	*Xylariales*	*Retama sphaerocarpa*
CF-286680	*Xylaria*	sp.	*Xylariales*	*Lygeum spartum*
CF-287438	*Xylaria*	sp.	*Xylariales*	*Ononis fruticosa*
CF-091951	*Xylaria*	sp.	*Xylariales*	*Rhamnus lycioides*
CF-285461	*Xylaria*	sp.	*Xylariales*	*Thymelaea hirsuta*
CF-090770	*Xylaria*	sp.	*Xylariales*	*Genista umbellata*

Supplementary Table 2. Fungal strains included in the phylogenetic analysis (**newly isolated strains** from plants collected in arid zones of Andalucía are **in bold**).

Species	Strain code [a]	Substrate	Origin	Genbank accesion numbers[b]	
				ITS	28S
***Cryptococcus* sp.**	**CF-285748**	***Beta macrocarpa***	**El Margen salt marsh, Granada, Spain**	**MG065727**	
***Coniothyrium* sp.**	**CF-091953**	***Anthyllis cihsoides***	**Sierra Alhamilla, Almeria, Spain**	**MG065728**	
***Coniothyrium* sp.**	**CF-090243**	***Genista umbellata***	**Tabernas desert, Almeria, Spain**	**MG065729**	
Coniothyrium palmarum	CBS 400.71	*Chamaerops humilis*	Italy	AY720708	JX681084
Coniothyrium carteri	CBS 105.91	*Quercus robur*	Germany	JF740181	KF251712
***Coniothyrium* sp.**	**CF-090330**	***Cistus albidus***	**Sierra Alhamilla, Almeria, Spain**	**MG065730**	
***Coniothyrium* sp.**	**CF-090362**	***Sedum sediforme***	**Sierra Alhamilla, Almeria, Spain**	**MG065731**	
Coniothyrium multiporum	CBS 353.65	saline soil	India	JF740187	JF740268
Coniothyrium telephii	CBS 188.71	air	Filand	JF740188	GQ387599
Fungal endophyte	SNP 431	*Parkinsonia microphylla*	Saguaro National Park, Arizona, USA	KP335607	
***Coniothyrium* sp.**	**CF-285356**	***Asparagus horridus***	**Tabernas desert, Almeria, Spain**	**MG065732**	
Hazslinszkyomyces aloes	CBS 136437	*Aloe dichotoma*	Western Cape Province, South Africa	KF777142	
***Hazslinszkyomyces* sp.**	**CF-090320**	***Artemisia sp.***	**Tabernas desert, Almeria, Spain**	**MG065733**	
Coniothyrina agaves	CBS 470.69	*Agave americana*	El Arenal, Mallorca, Spain	JX681075	
Neophaeosphaeria filamentosa	CBS 102202	*Yucca rostrata*	Mexico	JF740259	GQ387577
Camarosporium arezzoensis	MFLUCC 14-0238	*Cytisus* sp.	Italy	KP120926	KP120927
Camarosporium aureum	MFLUCC 14-0620	*Cotinus coggygria*	Russia	KP744436	KP744478
Camarosporium robiniicola	MFLUCC 13-0527	*Robinia pseudoacacia*	Italy	KJ562214	KJ589412
Camarosporium uniseriatum	MFLUCC 15-0444	*Celtis occidentalis*	Russia	KU697613	KU697614
***Camarosporium* sp.**	**CF-285350**	***Euzomodendron bourgeanum***	**Tabernas desert, Almeria, Spain**	**MG065734**	
Camarosporium spartii	MFLUCC 13-0548	Cytisus sp.	Italy	KJ562215	KJ589413
Camarosporium clematidis	MFLUCC 13-0336	*Clematis vitalba*	Italy	KJ562213	
Fungal endophyte	SNP 009	*Larrea tridentata*	Saguaro National Park, Arizona, USA	KP335215	
***Pseudoamarosporium* sp.**	**CF-285347**	***Helianthemum almeriense***	**Tabernas desert, Almeria, Spain**	**MG065735**	
Pseudocamarosporium propinquum	MFLUCC 13-0544	*Salix* sp.	Italy	KJ747049	KJ813280
Pseudocamarosporium lonicerae	MFLUCC 13-0532	*Lonicera* sp.	Italy	KJ747047	KJ813278
Pseudocamarosporium piceae	MFLUCC 14-0192	*Picea excels*	Italy	KJ747046	KJ803030
Pseudocamarosporium tilicola	MFLUCC 13-0550	*Pinnus nigra*	Italy	KJ747050	KJ813281
Pseudocamarosporium corni	MFLUCC 13-0541	*Cornus sanguinea*	Italy	KJ747048	KJ813279
***Pseudocamarosporium* sp.**	**CF-090324**	***Artemisia sp.***	**Tabernas desert, Almeria, Spain**	**MG065736**	
***Pseudocamarosporium* sp.**	**CF-090338**	***Anthyllis cihsoides***	**Sierra Alhamilla, Almeria, Spain**	**MG065737**	
Camarosporium brabeji	CBS 123026	*Protea* sp.	South Africa	EU552105	
Paracamarosporium hawaiiensis	CBS 1200225	*Sophora chrysophylla*	Hawaii, USA	DQ885897	
Paracamarosporium psoraleae	CBS 136628	*Psoralea pinnata*	Western Cape Province, South Africa	KF777143	KF777199
***Paracamarosporium* sp.**	**CF-090248**	***Genista umbellata***	**Tabernas desert, Almeria, Spain**	**MG065738**	

Paramarosporium leucadendri	CBS 123027	*Brabejum stellatifolium*	South Africa	EU552106	
Paracamarosporium tamaricis	MFLUCC 15-0495	*Tamarix gallica*	Italy	KU900327	KU900299
Paracamarosporium fagi	CPC 24892	*Fagus sylvatica*	Germany	KR611887	KR611905
Fungal endophyte	SNP 291	*Parkinsonia microphylla*	Saguaro National Park, Arizona, USA	KP335478	
Fungal sp.	ARIZ AZ0149	*Juniperus deppeana*	Coronado National Forest, Arizona, USA	HM122902	
***Camarosporium* sp.**	**CF-090314**	***Artemisia barrelieri***	**Tabernas desert, Almeria, Spain**	**MG065739**	
Kalmusia sarothamni	CBS 113833	*Cytisus sarothamni*	Sweden	KF796675	KF796671
***Kalmusia* sp.**	**CF-091181**	***Asparagus horridus***	**Tabernas desert, Almeria, Spain**	**MG065740**	
Kalmusia variispora	95SA2	*Quercus brantii*	Iran	KY783414	KY825094
Kalmusia ebuli	CBS 123120	*Populus tremula*	France	KF796674	
Kalmusia italica	MFLUCC 13-0066	*Spartium junceum*	Italy	KP325440	KP325441
Fungal sp.	ARIZ AZ0553	*Juniperus deppeana*	Coronado National Forest, Arizona, USA	HM123272	
Fungal sp.	ARIZ AZ0644	*Quercus rugosa*	Coronado National Forest, Arizona, USA	HM123362	
Xenodidymella applanata	CBS 102634	*Rubus idaeus*	Netherlands	GU237726	GU237997
***Xenodidymella* sp.**	**CF-092173**	***Bellota hirsuta***	**Sierra Alhamilla, Almeria, Spain**	**MG065741**	
***Xenodidymella* sp.**	**CF-090345**	***Onoris ramosissina***	**Sierra Alhamilla, Almeria, Spain**	**MG065742**	
***Xenodidymella* sp.**	**CF-288955**	***Thymus zygis subs gracillis***	**El Margen salt marsh, Granada**	**MG065743**	
***Xenodidymella* sp.**	**CF-288937**	***Sonchus crassifolius***	**El Margen salt marsh, Granada**	**MG065744**	
Neodidymelliopsis cannabis	CBS 121.75	*Urtica dioica*	Netherlands	GU237761	GU237972
Neodidymelliopsis xanthina	CBS 383.68	*Delphinium* sp.	Netherlands	NR_135994	GU238157
***Neodidymelliopsis* sp.**	**CF-286681**	***Cynanchum acutum***	**El Margen salt marsh, Granada**	**MG065745**	
Microsphaeropsis olivacea	CBS 303.68	*Ligustrum vulgare*	Netherlands	JX681101	
Boeremia exigua var exigua	CBS 431.74	*Solanum tuberosum*	Netherlands	FJ427001	JX681074
***Neomicrosphaeropsis* sp.**	**CF-090262**	***Tamarix canariensis***	**Tabernas desert, Almeria, Spain**	**MG065746**	
***Neomicrosphaeropsis* sp.**	**CF-288942**	***Tamarix canariensis***	**El Margen salt marsh, Granada**	**MG065747**	
***Neomicrosphaeropsis* sp.**	**CF-090785**	***Tamarix canariensis***	**Tabernas desert, Almeria, Spain**	**MG065748**	
Neomicrosphaeropsis tamaricicola	MFLUCC 14-0443	*Tamarix gallica*	Italy	KU900322	KU729851
***Neomicrosphaeropsis* sp.**	**CF-288902**	***Arthrocneum macrostachyum***	**El Margen salt marsh, Granada**	**MG065749**	
***Neomicrosphaeropsis* sp.**	**CF-285742**	***Arthrocneum macrostachyum***	**El Margen salt marsh, Granada**	**MG065750**	
***Neomicrosphaeropsis* sp.**	**CF-288907**	***Frankenia pulvirulenta***	**El Margen salt marsh, Granada**	**MG065751**	
***Neomicrosphaeropsis* sp.**	**CF-285767**	***Lycnium intricatum***	**Torre Garcia, Almeria, Spain**	**MG065752**	
Didymella exigua	CBS 183.55	*Rumex arifolius*	France	NR_135936	EU754155
***Didymella* sp.**	**CF-288938**	***Sonchus crassifolius***	**El Margen salt marsh, Granada**	**MG065753**	
Ascochyta medicaginicola	CBS 533.66	*Medicago sativa*	Netherlands	EU167575	
Leptosphaerulina australis	CBS 317.83	*Eugenia aromatica*	Indonesia	GU237829	EU754166
Leptosphaerulina australis	NBRC 33240	house dust	Japan	NBRC33240	
Leptosphaerulina trifolii	NBRC 7250	-	-	NBRC7250	
Leptosphaerulina argentinensis	CBS 569.94	*Lonicera periclymenum*	Netherlands	AY849947	
Ascochyta phacae	CBS 184.55	*Phaca alpina*	Switzerland	EU167570	

**_Ascochyta_ sp.**	**CF-090372**	**_Sedum sediforme_**	**Sierra Alhamilla, Almeria, Spain**	**MG065754**	
Ascochyta rabiei	CBS 237.37	Cicer arietinum	Bulgaria	EU167600	
**_Ascochyta_ sp.**	**CF-090376**	**_Bellota hirsuta_**	**Sierra Alhamilla, Almeria, Spain**	**MG065755**	
**_Ascochyta_ sp.**	**CF-090240**	**_Genista umbellata_**	**Tabernas desert, Almeria, Spain**	**MG065756**	
**_Ascochyta_ sp.**	**CF-091946**	**_Lycium intricatun_**	**Tabernas desert, Almeria, Spain**	**MG065757**	
**_Ascochyta_ sp.**	**CF-091945**	**_Launaea arboresceus_**	**Tabernas desert, Almeria, Spain**	**MG065758**	
**_Ascochyta_ sp.**	**CF-287439**	**_Ononis fruticosa_**	**El Margen salt marsh, Granada**	**MG065759**	
**_Ascochyta_ sp.**	**CF-090252**	**_Genista umbellata_**	**Tabernas desert, Almeria, Spain**	**MG065760**	
**_Ascochyta_ sp.**	**CF-090321**	**_Artemisia barrelieri_**	**Tabernas desert, Almeria, Spain**	**MG065761**	
**_Ascochyta_ sp.**	**CF-090305**	**_Launaea arborescens_**	**Tabernas desert, Almeria, Spain**	**MG065762**	
**_Ascochyta_ sp.**	**CF-090840**	**_Launaea arborescens_**	**Tabernas desert, Almeria, Spain**	**MG065763**	
**_Ascochyta_ sp.**	**CF-285366**	**_Rosmarinus eriocalyx_**	**Tabernas desert, Almeria, Spain**	**MG065764**	
**_Ascochyta_ sp.**	**CF-285355**	**_Asparagus horridus_**	**Tabernas desert, Almeria, Spain**	**MG065765**	
**_Ascochyta_ sp.**	**CF-287454**	**_Rosmarinus eriocalyx_**	**Tabernas desert, Almeria, Spain**	**MG065766**	
**_Ascochyta_ sp.**	**CF-287450**	**_Rosmarinus eriocalyx_**	**Tabernas desert, Almeria, Spain**	**MG065767**	
**_Ascochyta_ sp.**	**CF-287451**	**_Rosmarinus eriocalyx_**	**Tabernas desert, Almeria, Spain**	**MG065768**	
Ascochyta viciae-pannonicae	CBS 254.92	*Vicia pannonica*	Czechoslovakia	EU167559	KT389702
Ascochyta pisi var pisi	CBS 108.26	-	-	EU167557	
**_Phoma_ sp.**	**CF-092164**	**_Lygeum spartum_**	**Tabernas desert, Almeria, Spain**	**MG065769**	
Neosetophoma samarorum	CBS 139.96	grass	Netherlands	FJ427062	KF251665
Ascomycota sp.	ARIZ PAAsh314	*Fraxinus velutina*	Arizona, USA	JN120365	
Phaeosphaeria nigrans	NBRC 33095	*Phragmites karka*	Japan	NBRC33095	
Neosetophoma italica	MFLUCC 13-0388	*Iris germanica*	Italy	KP711356	KP711361
**_Neosetophoma_ sp.**	**CF-282001**	**_Retama sphaerocarpa_**	**Albuñuelas, Granada, Spain**	**KU295580**	
**_Neosetophoma_ sp.**	**CF-288948**	**_Teucrium pollium sbs gracillium_**	**El Margen salt marsh, Granada**	**MG065770**	
**_Phoma_ sp.**	**CF-090323**	**_Artemisia barrelieri_**	**Tabernas desert, Almeria, Spain**	**MG065771**	
**_Phoma_ sp.**	**CF-091947**	**_Suaeda vera_**	**Tabernas desert, Almeria, Spain**	**MG065772**	
Pyrenochaeta lycopersici	CBS 267.59	*Lycopersicon esculentum*	Netherlands	JF740261	GQ387612
Foliophoma fallens	CBS 167.78	*Olea europaea*	New Zealand	KY940772	GU238074
**_Foliophoma fallens_**	**CF-090782**	**_Asparagus horridus_**	**Tabernas desert, Almeria, Spain**	**MG065773**	
**_Libertasomyces_ sp.**	**CF-090382**	**_Bellota hirsuta_**	**Sierra Alhamilla, Almeria, Spain**	**MG065774**	
**_Libertasomyces_ sp.**	**CF-092175**	**_Bellota hirsuta_**	**Sierra Alhamilla, Almeria, Spain**	**MG065775**	
Libertasomyces platani	CPC 29609	*Platanus* sp.	New Zealand	KY173416	KY173507
Libertasomyces myopori	CPC 27354	*Myoporum serratum*	South Africa	KX228281	KX228332
Libertasomyces sp.	CF-092176	*Bellota hirsuta*	Sierra Alhamilla, Almeria, Spain	**MG065776**	
Neoplatysporoides aloicola	CBS 139901	Aloe sp.	Tanzania	KR476719	KR476754
**_Alternaria alternata_**	**CF-090395**	**_Arthrocneum macrostachyum_**	**Cabo de gata salt marsh, Almeria, Spain**	**MG065777**	
**_Alternaria alternata_**	**CF-090318**	**_Artemisia barrelieri_**	**Tabernas desert, Almeria, Spain**	**MG065778**	
Alternaria alternata	CBS 115200	*Citrus x tangelo*	South Africa	KP124352	KP124504

Alternaria alternata	CBS 118814	*Solanum lycopersicum*	USA	KP124357	KP124509
Alternaria alternata	**CF-288903**	***Arthrocneum macrostachyum***	**El Margen salt marsh, Granada**	**MG065779**	
Alternaria alternata	**CF-090322**	***Artemisia barrelieri***	**Tabernas desert, Almeria, Spain**	**MG065780**	
Alternaria alternata	CBS 102600	*Citrus reticulata*	USA	KP124331	KP124483
Alternaria alternata	**CF-288939**	***Suaeda vera***	**El Margen salt marsh, Granada**	**MG065781**	
Alternaria **sp.**	**CF-090254**	***Asparagus horridus***	**Tabernas desert, Almeria, Spain**	**MG065782**	
Alternaria gaisen	CBS 118488	*Pyrus pyrifolia*	Japan	KP124427	KP124581
Alternaria arborescens	CBS 102605	*Lycopersicon esculentum*	California	NR_135927	KC584253
Alternaria eichhorniae	CBS 119778	*Eichhornia crassipes*	Indonesia	KP124426	KP124580
Alternaria **sp.**	**CF-090752**	***Thymelaea hirsuta***	**Los Almorades, Almeria, Spain**	**MG065783**	
Alternaria **sp.**	**CF-090365**	***Sedum sediforme***	**Sierra Alhamilla, Almeria, Spain**	**MG065784**	
Alternaria terricola	CBS 202.67	wheat field soil	Northern Utah	NR_103600	KC584365
Alternaria	**CF-288921**	***Ononis tridentata***	**El Margen salt marsh, Granada**	**MG065785**	
Alternaria nobilis	CBS 116490	*Dianthus caryophyllus*	New Zealand	KC584208	KC584291
Alternaria infectoria	CBS 210.86	*Triticum aestivum*	England	NR_131263	KC584280
Alternaria ethzedia	CBS 197.86	*Brassica napus*	Switzerland	NR_135928	KC584274
Alternaria **sp.**	**CF-288904**	***Arthrocneum macrostachyum***	**El Margen salt marsh, Granada**	**MG065786**	
Alternaria **sp.**	**CF-282008**	***Retama sphaerocarpa***	**Albuñuelas, Granada, Spain**	**MG065787**	
Alternaria **sp.**	**CF-288922**	***Ononis tridentata***	**El Margen salt marsh, Granada**	**MG065788**	
Alternaria **sp.**	**CF-288961**	***Sonchus crassifolius***	**El Margen salt marsh, Granada**	**MG065789**	
Alternaria **sp.**	**CF-090246**	***Genista umbellata***	**Tabernas desert, Almeria, Spain**	**MG065790**	
Alternaria **sp.**	**CF-090261**	***Asparagus horridus***	**Tabernas desert, Almeria, Spain**	**MG065791**	
Alternaria **sp.**	**CF-288960**	***Frankenia pulvirulenta***	**El Margen salt marsh, Granada**	**MG065792**	
Alternaria penicillata	CBS 116607	*Papaver rhoeas*	Austria	KC584229	KC584322
Dendryphion papaveris	NBRC 9282	*Papaver somniferum*	-	NBRC9282	
Dendryphion papaveris	NBRC 9801	*Papaver somniferum*	Nagano, Japan	NBRC9801	
Alternaria solani	CBS 116651	*Solanum tuberosum*	USA	KC584217	KC584306
Alternaria cumini	**CF-090366**	***Sedum sediforme***	**Sierra Alhamilla, Almeria, Spain**	**MG065793**	
Alternaria cumini	CBS 121329	*Cuminum cyminum*	India	KC584191	KC584267
Stemphylium **sp.**	**CF-090250**	***Genista umbellata***	**Tabernas desert, Almeria, Spain**	**MG065794**	
Stemphylium **sp.**	**CF-090346**	***Onoris ramosissina***	**Sierra Alhamilla, Almeria, Spain**	**MG065795**	
Stemphylium **sp.**	**CF-090776**	***Genista umbellata***	**Tabernas desert, Almeria, Spain**	**MG065796**	
Stemphylium **sp.**	**CF-090781**	***Asparagus horridus***	**Tabernas desert, Almeria, Spain**	**MG065797**	
Paradendryphiella salina	CBS 142.60	*Spartina* sp.	Southampton, England	DQ411540	KF156158
Paradendryphiella salina	**CF-285749**	***Centaurea dracunculifolia***	**El Margen salt marsh, Granada**	**MG065798**	
Pleospora herbarum var herbarum	CBS 191.86	*Medicago sativa*	India	KC584239	JX681120
Pleospora herbarum	**CF-090771**	***Genista umbellata***	**Tabernas desert, Almeria, Spain**	**MG065799**	
Pleospora herbarum	NBRC 7404	-	-	NBRC7404	
Pleospora herbarum	**CF-285761**	***Inula crithmoides***	**Punta Entinas, Almeria, Spain**	**MG065800**	

Pleospora herbarum	**CF-288936**	***Sonchus crassifolius***	**El Margen salt marsh, Granada**	**MG065801**	
Pleospora herbarum	**CF-090249**	***Genista umbellata***	**Tabernas desert, Almeria, Spain**	**MG065802**	
Comoclathris sedi	MFLUCC 13-0763	*Rosa* sp.	Italy	KP334717	KP334707
Comoclathris **sp.**	**CF-090266**	***Lygeum spartum***	**Tabernas desert, Almeria, Spain**	**MG065803**	
Comoclathris **sp.**	**CF-287447**	***Rosmarinus eriocalyx***	**Tabernas desert, Almeria, Spain**	**MG065804**	
Comoclathris **sp.**	**CF-090792**	***Lygeum spartum***	**Tabernas desert, Almeria, Spain**	**MG065805**	
Comoclathris **sp.**	**CF-091934**	***Lygeum spartum***	**Tabernas desert, Almeria, Spain**	**MG065806**	
Comoclathris **sp.**	**CF-091944**	***Launaea arborescens***	**Tabernas desert, Almeria, Spain**	**MG065807**	
Comoclathris **sp.**	**CF-285379**	***Fagonia cretica***	**Tabernas desert, Almeria, Spain**	**MG065808**	
Comoclathris **sp**	**CF-282003**	***Retama sphaerocarpa***	**Albuñuelas, Granada, Spain**	**MG065809**	
Comoclathris **sp**	**CF-090361**	***Sedum sediforme***	**Sierra Alhamilla, Almeria, Spain**	**MG065810**	
Comoclathris **sp**	**CF-090763**	***Nerium oleander***	**Tabernas desert, Almeria, Spain**	**MG065811**	
Comoclathris spartii	MFLUCC 13-0214	*Spartium junceum*	Italy	KM577159	KM577160
Comoclathris **sp.**	**CF-091922**	***Ziziphus lotus***	**Los Almorades, Almeria, Spain**	**MG065812**	
Comoclathris **sp.**	**CF-090267**	***Lygeum spartum***	**Tabernas desert, Almeria, Spain**	**MG065813**	
Tamaricicola **sp.**	**CF-288959**	***Limonium majus***	**El Margen salt marsh, Granada**	**MG065814**	
Tamaricicola **sp.**	**CF-288916**	***Limonium majus***	**El Margen salt marsh, Granada**	**MG065815**	
Tamaricicola **sp.**	**CF-090279**	***Limonium insigne***	**Tabernas desert, Almeria, Spain**	**MG065816**	
Tamaricicola muriformis	MFLUCC 15-0488	*Tamarix gallica*	Italy	KU752187	KU561879
Tamaricicola muriformis	MFLUCC 15-0490	*Tamarix gallica*	Italy	KU752189	KU729856
Decorospora gaudefroyi	NBRC 32144	*Salicornia herbacea*	Lake Notoro, Hokkaido, Japan	NBRC32144	
Decorospora sp.	ATCC MYA-3203	*Sarcocornia perennis*	Red Bank, Northampton Co., VA, USA.	FJ914870	FJ914897
Phoma **sp.**	**CF-288917**	***Lygeum spartum***	**El Margen salt marsh, Granada**	**MG065817**	
Phoma **sp.**	**CF-090826**	***Thymus hyemalis***	**Tabernas desert, Almeria, Spain**	**MG065818**	
Phoma **sp.**	**CF-090829**	***Thymus hyemalis***	**Tabernas desert, Almeria, Spain**	**MG065819**	
Phoma **sp.**	**CF-090291**	***Thymus hyemalis***	**Tabernas desert, Almeria, Spain**	**MG065820**	
Neocamarosporium sp.	**CF-090392**	***Arthrocneum macrostachyum***	**Cabo de gata salt marsh, Almeria, Spain**	**MG065821**	
Neocamarosporium **sp.**	**CF-288932**	***Salsola vermiculata***	**El Margen salt marsh, Granada**	**MG065822**	
Neocamaroporium **sp.**	**CF-288928**	***Salsola vermiculata***	**El Margen salt marsh, Granada**	**MG065823**	
Neocamarosporium **sp.**	**CF-288962**	***Arthrocneum macrostachyum***	**El Margen salt marsh, Granada**	**MG065824**	
Neocamarosporium **sp.**	**CF-090841**	***Salsola genistoides***	**Tabernas desert, Almeria, Spain**	**MG065825**	
Neocamarosporium **sp.**	**CF-285744**	***Atriplex glauca***	**El Margen salt marsh, Granada**	**MG065826**	
Neocamarosporium **sp.**	**CF-285775**	***Suaeda vera***	**El Margen salt marsh, Granada**	**MG065827**	
Neocamarosporium **sp.**	**CF-285377**	***Suaeda vera***	**Tabernas desert, Almeria, Spain**	**MG065828**	
Neocamarosporium **sp.**	**CF-288934**	***Salsola vermiculata***	**El Margen salt marsh, Granada**	**MG065829**	
Neocamarosporium **sp.**	**CF-090309**	***Suaeda vera***	**Tabernas desert, Almeria, Spain**	**MG065830**	
Neocamarosporium chichastianum	**CF-285348**	***Salsola papIllosa***	**Tabernas desert, Almeria, Spain**	**MG065831**	
Neocamarosporium chichastianum	CBS 137502	saline soil	Iran	KP004455	KP004483
Neocamarosporium chersinae	CPC 27298	Dead angulate tortoise shell	South Africa	KY929153	KY929182

***Neocamarosporium* sp.**	**CF-090312**	***Suaeda vera***	**Tabernas desert, Almeria, Spain**	**MG065832**	
***Neocamarosporium* sp.**	**CF-285373**	***Salsola papollosa***	**Tabernas desert, Almeria, Spain**	**MG065833**	
***Neocamarosporium* sp.**	**CF-285768**	***Lycnium intricatum***	**Torre Garcia, Almeria, Spain**	**MG065834**	
***Neocamarosporium* sp.**	**CF-090390**	***Arthrocneum macrostachyum***	**Cabo de gata salt marsh, Almeria, Spain**	**MG065835**	
***Neocamarosporium* sp.**	**CF-287455**	***Lycium intricatum***	**Torre Garcia, Almeria, Spain**	**MG065836**	
***Dimorphosporicola* sp.**	**CF-090768**	***Suaeda vera***	**Tabernas desert, Almeria, Spain**	**MG065837**	
Dimorphosporicola tragani	CBS 570.85	*Traganum nudatum var. microphyll*	Mauritania	KU728497	KU728536
Dimorphosporicola tragani	**CF-090383**	***Arthrocneum macrostachyum***	**Cabo de gata salt marsh, Almeria, Spain**	**MG065838**	
Chaetosphaeronema hispidulum	CBS 826.88	soil	Israel	EU754145	
***Neocamarosporium* sp.**	**CF-090393**	***Arthrocneum macrostachyum***	**Cabo de gata salt marsh, Almeria, Spain**	**MG065839**	
***Neocamarosporium* sp.**	**CF-288930**	***Salsola vermiculata***	**El Margen salt marsh, Granada**	**MG065840**	
***Neocamarosporium* sp.**	**CF-285746**	***Atriplex glauca***	**El Margen salt marsh, Granada**	**MG065841**	
***Neocamarosporium* sp.**	**CF-285745**	***Atriplex glauca***	**El Margen salt marsh, Granada**	**MG065842**	
***Neocamarosporium* sp.**	**CF-285747**	***Beta macrocarpa***	**El Margen salt marsh, Granada**	**MG065843**	
Chaetodiplodia sp.	CBS 453.68	*Halimione portulacoides*	Netherlands	DQ678054	
***Neocamarosporium* sp.**	**CF-285349**	***Anabasis articulata***	**Tabernas desert, Almeria, Spain**	**MG065844**	
***Neocamarosporium* sp.**	**CF-285372**	***Anabasis articulata***	**Tabernas desert, Almeria, Spain**	**MG065845**	
***Neocamarosporium* sp.**	**CF-090335**	***Anthyllis cihsoides***	**Sierra Alhamilla, Almeria, Spain**	**MG065846**	
***Neocamarosporium* sp.**	**CF-285368**	***Anabasis articulata***	**Tabernas desert, Almeria, Spain**	**MG065847**	
***Neocamarosporium* sp.**	**CF-090759**	***Nerium oleander***	**Tabernas desert, Almeria, Spain**	**MG065848**	
Neocamarosporium goegapense	CBS 138008	*Mesembryanthemum* sp.	Northen Cape Province, South Africa	KJ869163	KJ869220
Neocamarosporium goegapense	**CF-090399**	***Chamaerops humilis***	**Cabo de gata salt marsh, Almeria, Spain**	**MG065849**	
***Neocamarosporium* sp.**	**CF-285352**	***Atriplex glauca***	**El Margen salt marsh, Granada**	**MG065850**	
***Neocamarosporium* sp.**	**CF-090227**	***Suaeda vera***	**Tabernas desert, Almeria, Spain**	**MG065851**	
***Neocamarosporium* sp.**	**CF-090284**	***Salicornia sp.***	**Tabernas desert, Almeria, Spain**	**MG065852**	
***Neocamarosporium* sp.**	**CF-090843**	***Salsola genistoides***	**Tabernas desert, Almeria, Spain**	**MG065853**	
***Neocamarosporium* sp.**	**CF-090773**	***Genista umbellata***	**Tabernas desert, Almeria, Spain**	**MG065854**	
Phaeosphaeriaceae sp.	DF-R-7	*Kochia scoparia*	QingDao, China	KU991885	KU991909
Neocamarosporium betae	CBS 523.66	*Beta vulgaris*	Netherlands	FJ426981	EU754179
Neocamarosporium betae	CBS 109410	*Beta vulgaris*	unknown	KY940790	EU754178
Neocamarosporium calvescens	CBS 344.78	*Atriplex hastata*	Netherlands	EU754132	
***Neocamarosporium* sp.**	**CF-090842**	***Salsola genistoides***	**Tabernas desert, Almeria, Spain**	**MG065855**	
Neocamarosporium calvescens	CBS 432.77	*Obione portulacoides*	Netherlands	GU230752	JF740267

[a] ATCC, American Type Culture Collection, University Boulevard Manassas VA, USA; CBS, CBS-KNAW Fungal Biodiversity Centre, Utrecht, the Netherlands; CF, Fundación MEDINA Private Fungal Collection, Granada, Spain; CPC. Culture collection of Pedro Crous, housed at CBS, Netherlands; MFLUCC, Mae Fah Luang University Culture Collection, Chiang Rai, Thailand; NBRC, Biological Resource Center, National Institute of Technology and Evolution, Tokyo, Japan

[b] Accession numbers of sequences newly generated in this study are indicated in bold. 28S, large subunit of the nrDNA; ITS, internal transcribed spacer regions of the nrDNA and intervening 5.8S nrDNA

Supplementary Table 3. Categorization of active isolates according to target strain. Chemical dereplication of fungal metabolites (otherwise specified). A (*Aspergillus fumigatus*), C (*Candida albicans*), M (*Magnaporthe grisea*), Ca (*Colletotrichum acutatum*) and H (HepG2).

		Exclusive Resin					Both					Exclusive No Resin					
**Strain**	**Taxonomy**	A	C	M	Ca	H	A	C	M	Ca	H	A	C	M	Ca	H	**LC-MS Dereplication**
CF-285778	*Albifimbria verrucaria*	0	0	0	0	0	1	1	1	1	1	0	0	0	0	0	illudin C2, roridin A &H, verrucarin A&B
CF-277739	*Trichothecium roseum*	0	0	0	0	0	1	1	1	1	1	0	0	0	0	0	trichothecene
CF-285757	*Trichothecium roseum*	0	0	0	0	0	1	1	1	1	1	0	0	0	0	0	trichothecene
CF-285465	*Coprinus cinereus*	0	0	1	0	0	1	1	0	1	1	0	0	0	0	0	skyrin, lagopodin B
CF-091944	*Comoclathris* sp.	0	0	0	0	0	0	0	1	0	0	1	1	0	1	1	phomasetin, altersetin
CF-285780	*Neosetophoma* sp.	0	0	0	0	0	1	0	1	0	1	0	0	0	1	0	globosuxanthone A
CF-285756	*Pseudodiplodia* sp.	1	0	0	0	0	0	0	1	0	1	0	0	0	1	0	globosuxanthone A, emodin
CF-282003	*Comoclathris* sp.	1	0	0	0	0	0	0	1	0	0	0	0	0	1	1	phomasetin, altersetin
CF-287447	*Comoclathris* sp.	1	0	0	0	0	0	0	0	0	0	0	0	1	1	1	phomasetin, altersetin
CF-285362	*Dendryphion* sp.	0	0	0	0	0	0	0	1	0	0	0	0	0	1	1	phomasetin
CF-287464	*Pyrenochaeta* sp.	0	0	0	0	0	0	0	0	0	0	0	0	1	1	1	$C_{29}H_{41}NO_5$ (previously described in plants)
CF-090752	*Alternaria* sp.	0	0	1	0	0	1	0	0	0	1	0	0	0	0	0	dextrusin B, B4, terpestacin
CF-279239	*Coniochaeta* sp.	0	0	0	0	0	1	0	0	0	1	0	0	1	0	0	Curvicolilde A/B, xylactam, $C_{26}H_{42}O_5$ (peviosly described in plants)
CF-091924	*unidentified*	0	0	0	0	0	1	0	1	0	0	0	0	0	0	1	posible antibiotic YW 3548, $C_{47}H_{75}N_7O_{12}$ and $C_{46}H_{73}N_7O_{12}$ (no match in DNP)
CF-091922	*Comoclathris* sp.	0	0	1	0	0	0	0	0	0	1	0	0	0	0	0	illudin C3, $C_{26}H_{43}NO_6$ (previously described in plants)
CF-091951	*Xylaria* sp.	0	0	1	0	0	0	0	0	0	1	0	0	0	0	0	nectriapyrone, cytochalasin C/D/M/Q
CF-279244	*Coprinopsis episcopalis*	0	0	1	0	0	0	0	0	0	1	0	0	0	0	0	lagopodin A & B
CF-282344	*unidentified*	0	0	0	0	1	0	0	1	0	0	0	0	0	0	0	phomasetin
CF-091933	*Pleospora* sp.	0	0	0	0	0	0	0	0	0	0	0	0	1	0	1	phomasetin, altersetin
CF-090383	*Dimorphosporicola traganii*	1	1	0	0	1	0	0	0	0	0	0	0	0	0	0	cerulenin
CF-090835	*Preussia* sp.	0	0	0	0	1	0	1	0	0	0	0	0	0	0	0	di-hydro-bi-chloro-geodin, TMC 120 B & C
CF-090227	*Neocamarosporium* sp.	0	0	0	0	0	1	0	0	0	1	0	0	0	0	0	leptosphaeridione
CF-090766	*Comoclathris* sp.	1	0	0	0	0	0	0	0	0	1	0	0	0	0	0	possible coriolide
CF-288925	*unidentified*	1	0	0	0	0	0	0	0	0	1	0	0	0	0	0	hormonemate, possible coriolide
CF-090379	*Camarosporium* sp.	0	0	0	0	0	1	0	0	0	0	0	0	0	0	1	naematolin, naematolone, roridin L2
CF-286681	*Neodidymelliopsis* sp.	0	0	0	0	0	1	0	0	0	0	0	0	0	0	1	waol A
CF-285753	*Pseudodiplodia ruticola*	0	0	0	0	0	1	0	0	0	0	0	0	0	0	1	violaceol, $C_{22}H_{24}O_{11}S$ (no match in DNP)
CF-285754	*Pseudodiplodia ruticola*	0	0	0	0	0	1	0	0	0	0	0	0	0	0	1	possible 2-(3,4-Epoxy-5-heptenoyl)-5-methylpyrrole, $C_{22}H_{24}O_{11}S$ (no match in DNP)
CF-090353	*Lepteutypa cupressi*	0	0	0	0	1	0	0	0	0	0	1	0	0	0	0	MDN-210 ($C_{17}H_{24}O_4$), pestahivin
CF-090758	*unidentified*	1	0	0	0	0	0	0	0	0	0	0	0	0	0	1	possible coriolide
CF-090357	*Leptosphaeria hispanica*	0	0	0	0	0	0	0	0	0	0	1	0	0	0	1	massarigenin A, cordyol C, dehydromassarilactone D, phomasetin
CF-090361	*Comoclathris* sp.	0	0	0	0	0	0	0	0	0	0	1	0	0	0	1	2-(2-Hydroxy-5-metgosyphenoxy) acrylic acid, $C_{18}H_{30}O_4S$ (no math in DNP), possible coriolide
CF-287440	*Aureobasidium* sp.	0	0	0	0	0	0	0	0	0	0	1	0	0	0	1	$C_{48}H_{78}O_{18}$ (previously described from plants)
CF-090213	*Eutypa consobrina*	1	0	0	0	1	0	0	0	0	0	0	0	0	0	0	MDN-209 ($C_{17}H_{26}O_4$), MDN-210 ($C_{17}H_{24}O_4$), MDN-211 ($C_{10}H_{12}O_4$)

CF-090748	*unidentified*	1	0	0	0	0	0	0	0	0	0	0	0	0	0	1	possible cerebroside B
CF-281556	*Stagnospora* sp.	0	0	0	1	0	1	1	1	0	0	0	0	0	0	0	secalonic acid C, possible alternethanoxin A
CF-090324	*Pseudocamarosporium* sp.	0	1	1	1	0	0	0	0	0	0	1	0	0	0	0	calbistrin A
CF-285462	*Fusarium equiseti*	0	0	0	0	0	0	1	0	0	0	0	0	1	1	0	equisetin
CF-090393	*Neocamarosporium* sp.	1	0	0	1	0	0	0	0	0	0	0	0	0	0	0	possible solanopyrone B
CF-285355	*Ascochyta* sp.	0	0	0	0	0	0	0	0	0	0	1	0	1	0	0	cephalochromin and cercosporamide
CF-285768	*Neocamarosporium* sp.	0	0	1	0	0	0	0	0	0	0	1	0	0	0	0	terrecyclic acid A
CF-090792	*Comoclathris* sp.	0	0	0	0	0	0	0	0	0	0	1	0	1	0	0	phomasetin, altersetin
CF-288952	*Phaeosphaeria* sp.	0	0	0	0	0	0	0	0	0	0	0	0	1	1	0	possible brefeldin A and mycophenolic acid
CF-287454	*Ascochyta* sp.	0	0	0	0	0	0	1	0	0	0	1	0	0	0	0	cercosporamide
CF-090351	*Hormonema carpetanum*	0	0	0	0	0	1	0	0	0	0	0	1	0	0	0	$C_{30}H_{54}O_{14}$ (no match in DNP)
CF-285358	*Phaeotheca triangularis*	0	0	0	0	0	1	0	0	0	0	0	1	0	0	0	possible coriolide $C_{18}H_{30}O_2$
CF-285461	*Xylaria* sp.	1	1	0	0	0	0	0	0	0	0	0	0	0	0	0	antibiotic TKR2648
CF-285752	*Cryptococcus* sp.	0	1	0	0	0	0	0	0	0	0	1	0	0	0	0	$C_{15}H_{20}O_3$
CF-090267	*Comoclathris* sp.	0	0	0	0	0	0	0	0	0	0	1	1	0	0	0	possible coriolide and nematolin
CF-287457	*Setomelanomma* sp.	0	0	0	0	0	0	0	0	0	0	1	1	0	0	0	11,12-dihydroxyeudesm-4-en-3-one, possible coriolide
CF-288933	*Preussia australis*	1	1	0	0	0	0	0	0	0	0	0	0	0	0	0	$C_{18}H_{33}NO$ (previously described in plants)
CF-277101	*Selenophoma juncea*	0	0	0	0	0	0	0	0	0	1	0	0	0	0	0	heptelidic acid (avocettin)
CF-090350	*Anthostomella* sp.	0	0	0	0	0	0	0	0	0	1	0	0	0	0	0	heptidelic and hydroheptidelic acid
CF-090359	*Stagnospora* sp.	0	0	0	0	0	0	0	0	0	1	0	0	0	0	0	11,12-dihydroxyeudesm-4-en-3-one, diorcinol F, altersetin, phomasetin
CF-090782	*Foliophoma* sp.	0	0	0	0	0	0	0	0	0	1	0	0	0	0	0	$C_{39}H_{27}NO_8S$ (no match in DNP)
CF-090785	*Neomicrosphaeropsis* sp.	0	0	0	0	0	0	0	0	0	1	0	0	0	0	0	possible ACTG Toxin C
CF-090828	*Lophiostoma* sp.	0	0	0	0	0	0	0	0	0	1	0	0	0	0	0	oxasetin, cytochalasin, $C_{22}H_{22}O_8$ (previously described in plants)
CF-092173	*Xenodidymella sp.*	0	0	0	0	0	0	0	0	0	1	0	0	0	0	0	possible coriolide, phomasetin
CF-092175	*Libertasomyces sp.*	0	0	0	0	0	0	0	0	0	1	0	0	0	0	0	possible naematolin
CF-092179	*unidentified*	0	0	0	0	0	0	0	0	0	1	0	0	0	0	0	$C_{29}H_{33}N_3O_6$ (previously described in plants)
CF-278520	*Phoma* sp.	0	0	0	0	0	0	0	0	0	1	0	0	0	0	0	curvicolide A/B, $C_{26}H_{42}O_5$ (peviosly described in palnts)
CF-279248	*Anthostomella* sp.	0	0	0	0	0	0	0	0	0	1	0	0	0	0	0	$C_{26}H_{42}O_5$ (peviosly described in palnts)
CF-285767	*Neomicrosphaeropsis* sp.	0	0	0	0	0	0	0	0	0	1	0	0	0	0	0	phomasetin
CF-286679	*Chaetomium* sp.	0	0	0	0	0	0	0	0	0	1	0	0	0	0	0	possible sphaeropsidin B, $C_{26}H_{42}O_5$ (peviosly described in palnts)
CF-090249	*Pleospora herbarum*	0	0	0	0	1	0	0	0	0	0	0	0	0	0	0	possible coriolide
CF-090313	*Morinia* sp.	0	0	0	0	1	0	0	0	0	0	0	0	0	0	0	MDN-209 ($C_{17}H_{26}O_4$)
CF-090838	*Preussia grandispora*	0	0	0	0	1	0	0	0	0	0	0	0	0	0	0	mellein
CF-285373	*Neocamarosporium* sp.	0	0	0	0	1	0	0	0	0	0	0	0	0	0	0	spirostaphylotrichin (C, D, H o G)
CF-290943	*unidentified*	0	0	0	0	1	0	0	0	0	0	0	0	0	0	0	brefeldin A, $C_{16}H_{22}O_8$ possible macrosphelide
CF-287465	*Aureobasidium pullulans*	0	0	0	0	1	0	0	0	0	0	0	0	0	0	0	possible exophilin A, $C_{40}H_{74}O_{13}$ (no match in DNP)
CF-285363	*Phaeosphaeria* sp.	0	0	0	0	0	0	0	0	0	0	0	0	0	0	1	elsinochrome D, possible phaeosphaerin C
CF-090221	*Verrucocladosporium* sp.	0	0	0	0	0	0	0	0	0	0	0	0	0	0	1	$C_{19}H_{30}O_9$ (previously described in plants), cyclo(Pro-Phe)
CF-090254	*Alternaria* sp.	0	0	0	0	0	0	0	0	0	0	0	0	0	0	1	$C_{12}H_{19}NO_3$ (previous described in plants)

CF-090330	*Coniothyrium sp.*	0	0	0	0	0	0	0	0	0	0	0	0	0	0	1	brefeldin A, $C_{13}H_{17}NO_2$ (previously descrined in plants)
CF-090371	*Morinia pestalozzioides*	0	0	0	0	0	0	0	0	0	0	0	0	0	0	1	moriniafungin
CF-090372	*Ascochyta* sp.	0	0	0	0	0	0	0	0	0	0	0	0	0	0	1	$C_{48}H_{78}O_{18}$ (previously described in plants)
CF-090382	*Libertasomyces* sp.	0	0	0	0	0	0	0	0	0	0	0	0	0	0	1	naematolin, cytosporone D, cordyol E
CF-090768	*Dimorphosporicola* sp.	0	0	0	0	0	0	0	0	0	0	0	0	0	0	1	cerulenin
CF-091931	*Chaetothyriales* sp.	0	0	0	0	0	0	0	0	0	0	0	0	0	0	1	tentative ilicicolin H and ascochlorin
CF-278800	*Dothiorella* sp.	0	0	0	0	0	0	0	0	0	0	0	0	0	0	1	4-Hydroxymellein
CF-285741	*Neomicrosphaeropsis* sp.	0	0	0	0	0	0	0	0	0	0	0	0	0	0	1	phomasetin
CF-285749	*Paradendryphiella salina*	0	0	0	0	0	0	0	0	0	0	0	0	0	0	1	waol A
CF-287476	*Fusarium* sp.	0	0	0	0	0	0	0	0	0	0	0	0	0	0	1	fusaric acid, 3-Hydroxymellein
CF-288910	*Aureobasidium pullulans*	0	0	0	0	0	0	0	0	0	0	0	0	0	0	1	$C_{26}H_{43}NO_6$ and $C_{20}H_{38}O_7$ (previously described in plants)
CF-285750	*Cryptococcus* sp.	0	0	0	0	0	1	0	0	0	0	0	0	0	0	0	lovastatin acid
CF-285759	*Entodesmium* sp.	0	0	0	0	0	1	0	0	0	0	0	0	0	0	0	pycnidione, $C_{33}H_{40}O_7$ (no match in DNP)
CF-285364	*Pleiochaeta* sp.	0	0	0	0	0	1	0	0	0	0	0	0	0	0	0	phomasetin
CF-090228	*Neoamarosporium* sp.	0	0	0	0	0	1	0	0	0	0	0	0	0	0	0	phomasetin
CF-285758	*Ascochyta* sp.	0	0	0	0	0	1	0	0	0	0	0	0	0	0	0	emodin, possible pestalamide A, $C_{22}H_{21}O_8$ (no match in DNP)
CF-288957	*Xenodidymella* sp.	0	0	0	0	0	1	0	0	0	0	0	0	0	0	0	hormonemate, $C_{30}H_{54}O_{14}$ (no match in DNP)
CF-285350	*Camarosporium* sp.	1	0	0	0	0	0	0	0	0	0	0	0	0	0	0	$C_{17}H_{31}NO_3$ and $C_{16}H_{29}NO_4$ (previously described in plants)
CF-285357	*Preussia similis*	1	0	0	0	0	0	0	0	0	0	0	0	0	0	0	pleofungin A
CF-285375	*Preussia australis*	1	0	0	0	0	0	0	0	0	0	0	0	0	0	0	$C_{18}H_{33}NO$ (previously described in plants)
CF-285376	*Basifimbria* sp.	1	0	0	0	0	0	0	0	0	0	0	0	0	0	0	cyclo (phenylalanylprolyl)
CF-285755	*Pseudodiplodia ruticola*	1	0	0	0	0	0	0	0	0	0	0	0	0	0	0	possible 6-methyl-9-heptadecenoic acid, $C_{22}H_{24}O_{11}S$ (no match in DNP)
CF-287456	*Teratosphaeria* sp.	0	0	0	0	0	0	0	0	0	0	1	0	0	0	0	3-indolylacetic acid
CF-090284	*Neoamarosporium* sp.	0	0	0	0	0	0	0	0	0	0	1	0	0	0	0	leptosphaerodione
CF-091947	*Phoma sp.*	0	0	0	0	0	0	0	0	0	0	1	0	0	0	0	massarigenin A, hormonemate
CF-285378	*Preussia* sp.	0	0	0	0	0	0	0	0	0	0	1	0	0	0	0	$C_{21}H_{33}N_3O_2$ (previously described in plants)
CF-285751	*Septoria* sp.	0	0	0	0	0	0	0	0	0	0	1	0	0	0	0	$C_{26}H_{43}NO_6$ and $C_{27}H_{45}NO_6$ (previously described in plants)
CF-285760	*Cryptococcus* sp.	0	0	0	0	0	0	0	0	0	0	1	0	0	0	0	$C_{18}H_{32}N_8O_{10}$ (no match in DNP)
CF-285765	*Paraconiothyrium* sp.	0	0	0	0	0	0	0	0	0	0	1	0	0	0	0	$C_{36}H_{22}O_6$ (no match in DNP)
CF-285774	*Selenophoma juncea*	0	0	0	0	0	0	0	0	0	0	1	0	0	0	0	possible phomotone, norteanuazonic acid
CF-285777	*Dothiorella* sp.	0	0	0	0	0	0	0	0	0	0	1	0	0	0	0	dechlorogriseofulvin, griseofulvin
CF-285772	*Preussia* sp.	0	0	0	0	0	0	1	0	0	0	0	0	0	0	0	di-hydro-bi-chloro-geodin, asterric acid
CF-285773	*Preussia* sp.	0	1	0	0	0	0	0	0	0	0	0	0	0	0	0	cerulenin analog, asterric acid, $C_{18}H_{32}N_8O_{10}$ (No match in DNP)
CF-090317	*Selenophoma* sp.	0	1	0	0	0	0	0	0	0	0	0	0	0	0	0	$C_{26}H_{43}NO_6$ (previously described in plants), cyclo(phenylalanylprolyl)
CF-288939	*Alternaria* sp.	0	0	0	0	0	0	0	0	0	0	0	1	0	0	0	nortenuazonic and tenuazonic acid
CF-288959	*Tamaricicola* sp.	0	0	0	0	0	0	0	0	0	0	0	1	0	0	0	cyclo (phenylalanylprolyl), possible phenopyrrozin
CF-092176	*Libertasomyces* sp.	0	0	0	0	0	0	0	1	0	0	0	0	0	0	0	possible naematolin, $C_{24}H_{30}O_5$
CF-282335	*Homortomyces tamaricis*	0	0	0	0	0	0	0	1	0	0	0	0	0	0	0	cephalochromin, TAN 2483 A and B
CF-090258	*Stagnospora* sp.	0	0	1	0	0	0	0	0	0	0	0	0	0	0	0	secalonic acid C, 11,12-dihydroxyeudesm-4-en-3-one, iludinic acid

CF-279249	*Cainia* sp.	0	0	1	0	0	0	0	0	0	0	0	0	0	0	0	cyclo (phenylalanylprolyl)
CF-285365	*Phoma* sp.	0	0	1	0	0	0	0	0	0	0	0	0	0	0	0	cercosporamide
CF-285743	*Epicoccum* sp.	0	0	1	0	0	0	0	0	0	0	0	0	0	0	0	tenuazonic acid, mellein, phomasetin
CF-285771	*Nigrospora* sp.	0	0	1	0	0	0	0	0	0	0	0	0	0	0	0	possible taurocholic acid
CF-287451	*Phoma* sp.	0	0	1	0	0	0	0	0	0	0	0	0	0	0	0	mycophenolic acid
CF-090778	*Stagnospora* sp.	0	0	0	0	0	0	0	0	0	0	0	0	1	0	0	11,12-dihydroxyeudesm-4-en-3-one, possible gonytolide F
CF-279243	*Leptospora* sp.	0	0	0	0	0	0	0	0	0	0	0	0	1	0	0	possible chrysoxanthone
CF-279245	*Pseudopithomyces chartarum*	0	0	0	0	0	0	0	0	0	0	0	0	1	0	0	possible antibiotic LL-N 313ε
CF-285353	*Dothiora* sp.	0	0	0	0	0	0	0	0	0	0	0	0	1	0	0	hormonemate
CF-285371	*Microascus trigonosporus*	0	0	0	0	0	0	0	0	0	0	0	0	1	0	0	petasol
CF-090323	*Phoma* sp.	0	0	0	1	0	0	0	0	0	0	0	0	0	0	0	antibiotic PF 1163A
CF-090387	*Camarosporium* sp.	0	0	0	1	0	0	0	0	0	0	0	0	0	0	0	possible coriolide
CF-288938	*Didymella* sp.	0	0	0	1	0	0	0	0	0	0	0	0	0	0	0	cyclo (phenylalanylprolyl), $C_{39}H_{27}NO_8S$ (no match in DNP)
CF-282337	*Ascochyta* sp.	0	0	0	0	0	0	0	0	0	0	0	0	0	1	0	possible sequalestatin H1, $C_{37}H_{52}O_{15}$ (previously described in plants)
**TOTAL**		17	7	13	6	11	22	9	13	4	26	22	7	13	10	30	

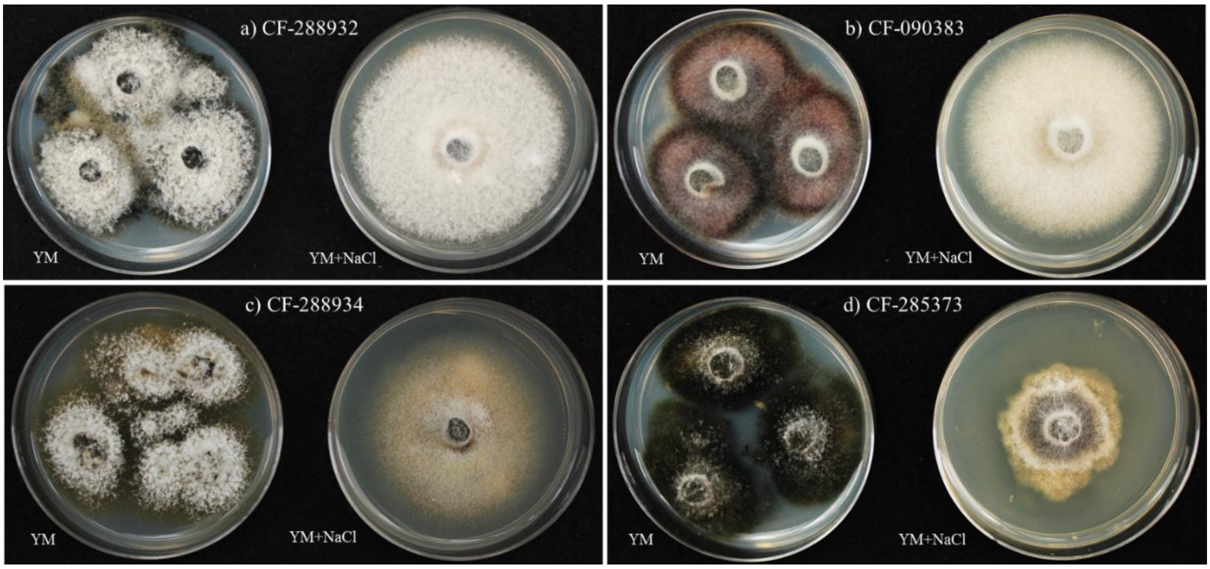

Supplementary Figure 2 a-d. Different isolates of *Neocamarosporium* spp grown on yeast malt agar (YM) and yeast malt agar supplemented with NaCl at 3% (YM+NaCl) during 14 days at 22ºC and 70% of relative humidity.

Supplementary Table 4. List of collected plant specimens.

Plant Species	Collection place	Ecology	Termophile	Xerophile	Gypsophile	Halophile	Salt Tolerant
Anabasis articulata	Tabernas desert (Almeria)	Desert	x	x		x	
Anthyllis cystisoides	Sierra de la Alhamilla (Almeria)	Semiarid	x				
Anthyllis terniflora	Tabernas desert (Almeria)	Desert	x	x			
Artemisia barrelieri	Tabernas desert (Almeria)	Desert		x			
Arthrocnemum macrostachyum	Cabo de Gata (Almeria)	Salt mars	x	x		x	
Asparagus horridus	Tabernas desert (Almeria)	Desert	x	x			
Atriplex glauca	El Margen (Granada)	Salt mars	x	x		x	
Ballota hirsuta	Sierra de la Alhamilla (Almeria)	Semiarid		x			
Beta macrocarpa	El Margen (Granada)	Salt mars	x			x	
Centaurea dracunculifolia	El Margen (Granada)	Salt mars	x			x	
Chamaerops humilis	Cabo de Gata (Almeria)	Salt mars	x				
Cistus albidus	Sierra de la Alhamilla (Almeria)	Semiarid		x			
Cynanchum acutum	El Margen (Granada)	Salt mars	x			x	
Dittrichia viscosa	Venta de Fraile (Granada)	Semiarid					
Dorycnium pentaphyllum	Punta Entinas (Almeria)	Semiarid					
Euzomodendron bourgeanum	Tabernas desert (Almeria)	Desert	x			x	
Fagonia cretica	Tabernas desert (Almeria)	Desert	x			x	
Frankenia pulverulenta	El Margen (Granada)	Salt mars	x			x	
Frankenia corymbosa	Tabernas desert (Almeria)	Desert	x			x	
Genista umbellata	Tabernas desert (Almeria)	Desert	x				
Gypsophila tomentosa	El Margen (Granada)	Salt mars	x		x	x	
Helianthemum almeriense	Tabernas desert (Almeria)	Desert	x				x
Limbarda crithmoides	Punta Entinas (Almeria)	Semiarid	x			x	
Juniperus oxycedrus	Alfacar (Granada)	Semiarid		x			
Launaea arborescens	Tabernas desert (Almeria)	Desert	x	x			
Lepidium subulatum	El Margen (Granada)	Salt mars			x		
Limodorum abortivum	Barranco de los Pinos (Granada)	Semiarid	x				
Limonium insigne	Tabernas(Almeria)	Desert	x			x	
Limonium majus	El Margen (Granada)	Salt mars				x	
Lycium intricatum	Torre Garcia (Almeria)	Semiarid	x				x
Lygeum spartum	Tabernas(Almeria)	Desert		x			x

Macrosyringion longiflorum	El Margen (Granada)	Salt mars		x			
Moricandia foetida	Tabernas (Almeria)	Desert	x				x
Nerium oleander	Tabernas(Almeria)	Desert	x	x			
Nicotiana glauca	Rodalquilar (Almeria)	Semiarid	x				
Ononis tridentata	El Margen (Granada)	Salt mars			x		x
Ononis fruticosa	El Margen (Granada)	Salt mars		x			
Onoris ramosissina	Sierra de la Alhamilla (Almeria)	Semiarid				x	
Opuntia ficus-indica	Dilar (Granada)	Semiarid		x			
Otanthus maritimus	Torre Garcia (Almeria)	Semiarid	x			x	
Phragmites australis	El Margen (Granada)	Salt mars	x				x
Pinus halepenssis	Sierra de la Alhamilla (Almeria)	Semiarid	x				
Retama sphaerocarpa	Tabernas (Almeria)	Semiarid	x				
Rhamnus lycioides	Sierra de la Alhamilla (Almeria)	Semiarid		x			
Rosmarinus eriocalyx	Tabernas (Almeria)	Desert	x	x			
Salicornia ramossissima	Tabernas(Almeria)	Desert	x			x	
Salsola genistoides	Tabernas (Almeria)	Desert				x	
Salsola oppositifolia	Tabernas (Almeria)	Desert				x	
Salsola papillosa	Tabernas (Almeria)	Desert				x	
Salsola vermiculata	El Margen (Granada)	Salt mars				x	
Sedum sediforme	Sierra de la Alhamilla (Almeria)	Semiarid		x			
Sonchus crassifolius	El Margen (Granada)	Salt mars				x	
Spartium junceum	Fuente de Hervidero (Granada)	Semiarid		x			
Stipa tenacissima	Albuñuelas (Granada)	Semiarid		x			
Suaeda vera	Tabernas desert (Almeria)	Desert				x	
Tamarix canariensis	Tabernas desert (Almeria)	Desert	x	x		x	
Teucrium capitatum sbsp. gracillimum	El Margen (Granada)	Salt mars		x			
Thymelaea hirsuta	Los Almorades (Almeria)	Semiarid		x			
Thymus hyemalis	Tabernas desert (Almeria)	Desert	x	x			x
Thymus zygis subsp. gracilis	El Margen (Granada)	Salt mars		x			
Withania frutescens	Almerimar (Almeria)	Semiarid	x	x			x
Ziziphus lotus	Torre Garcia (Almeria)	Semiarid		x			x
Zygophyllum fabago	Torre Garcia (Almeria)	Semiarid	x			x	

Structure elucidation of new natural products

MDN-0209 (**1**) was isolated as a yellow pale oil. A molecular formula of $C_{17}H_{26}O_4$ was determined from HRESIMS data (*m/z* 277.1800 ([M + H - H_2O]$^+$; calcd. for $C_{17}H_{25}O_3^+$, 277.1798), requiring five double bond equivalents. The ^{1}H, ^{13}C-NMR and (**Table S1**) HSQC data, accounted for the presence in the molecule of three double bonds (δ_C 134.0, C-3; δ_C 131.5, C-4; δ_H 5.23, δ_C 118.8, CH-8; δ_C 136.2, C-9; δ_H 6.42, δ_C 126.9, CH-12; δ_H 6.08, δ_C 134.9, C-13). Two allylic methylenes (δ_H 2.83 and 2.37, δ_C 30.9, CH_2-7; δ_H 2.15, δ_C 36.6, CH_2-14), one aliphatic triplet methyl group (δ_H 0.94, δ_C 14.0, CH_3-16), two allylic singlet methyls (δ_H 1.67, δ_C 18.1, CH_3-10; δ_H 1.74, δ_C 26.0, CH_3-11) and one aliphatic methylenes (δ_H 1.47, δ_C 23.5, CH_2-15) were also observed. Additionally, several oxygenated carbons were present in the molecule: one quaternary carbon (δ_C 61.1, C-1), three methines (δ_H 4.43, δ_C 67.6, CH-2; δ_H 4.61, δ_C 65.2, CH-5; δ_H 3.21, δ_C 59.5, CH-6) and one methylene (δ_H 4.39 and 4.15, δ_C 59.9, CH_2-17). COSY (**Figure S1**) and HMBC (**Figure S2**) spectra helped to construct the planar structure, which contained one epoxide and one six carbons ring, completing the total number of unsaturations. The presence of the epoxide was justified by the shielded chemical shift of carbons C-1 and C-6 respect to the rest of oxygenated carbons. An *E* configuration was proposed for the double bond C-12/C-13 based on the value of the H-12/H-13 coupling constant (J = 15.7 Hz). The NOESY interactions observed between protons H-7 and H-10, and those between H-8 and H-11 secured the identity of the two allylic methyl groups. The relative configuration around the six members ring was supported by key NOESY correlations between protons H-2, H_2-7 and H-6, indicating that all of them are on the same face of the molecule. Finally, the absolute configuration at C-2 and C-5 was determined applying the modified Mosher's method (1) to the R- and S-MTPA esters of both secondary hydroxyl groups. Although the ^{1}H gave an irregular distribution of $\Delta\delta^{SR}$ values, the configuration could eventually be determined by analysis of the $\Delta\delta^{SR}$ distribution for the ^{13}C signal (**Figure S10**), indicating an *S* configuration in both centers.

MDN-0210 (**2**) was isolated as a brown pale oil. A molecular formula of $C_{17}H_{24}O_4$ was determined from HRESIMS (*m/z* 293.1747 ($[M + H]^+$; calcd. for $C_{17}H_{25}O_4^+$, 293.1747), requiring six degrees of unsaturation. Two protons less in the molecular formula of **2** with respect to **1** and the analysis of the ^1H and ^{13}C-NMR spectra (**Table S1**) indicated that the hydroxyl group at C-5 in **1** was oxidized to a carbonyl group in **2**. The HMBC correlation between H-6 (δ_H 3.30) and C-5 (δ_C 198.1) supported this change (**Figure S12**). The rest of the planar structure was confirmed by COSY (**Figure S11**) and HMBC spectra, and proved to be similar to compound **1**. On the other hand, the NOESY interactions observed between protons 2, 7 and 6 supported the relative configuration proposed and the absolute configuration was assumed to be the same as in compound **1**.

MDN-0211 (**3**) was isolated as a white amorphous solid. Its HRESIMS analysis gave a molecular ion a *m/z* 235.0574 ($[M + Na]^+$; calcd. for $C_{10}H_{12}NaO_5^+$, 235.0577), which was consistent with the molecular formula $C_{10}H_{12}O_5$, requiring five double bond equivalents. The ^1H and ^{13}C-NMR spectra (**Table S2**) showed signals for two methoxy groups (δ_H 3.81, δ_C 56.6, CH_3-9; δ_H 3.88, δ_C 57.1, CH_3-10), one oxygenated methylene (δ_H 5.06 and 4.42, δ_C 71.5, CH_2-8), one double oxygenated methine (δ_H 6.12, δ_C 107.6, CH_3-1) and one aromatic ring (δ_H 6.57, δ_C 98.1, CH-4; δ_C 118.6, C-2; δ_C 149.6, C-3; δ_C 150.7, C-5; δ_C 135.1, C-6; δ_C 129.5, C-7). The analysis of HMBC spectrum (**Figure S20**) determined the connectivity of these units. The methoxy protons of OCH_3-9 and OCH_3-10 had an HMBC correlation to C-3 and C-5, respectively, on the aromatic moiety. HMBC correlations from H_2-8 to C-1 and from H-1 to C-8 and the chemical shift of these hydrogen and carbon atoms in the ^1H and ^{13}C NMR spectra secured the presence of an oxygen bridge between both carbon atoms and hence of a heteroaromatic ring in the structure of **3**. Finally, the chemical shift of C-6 (δ_C 135.1) was indicative of the presence of a hydroxyl group at this carbon, completing the initially determined chemical formula. The absence of specific rotation in the sample confirmed the existence of a mixture of enantiomers in this sample. Most probably, compound **3** is formed by nucleophilic attack of a primary hydroxyl group at C-8 to an aldehyde carbonyl group at C-1 equally favored in both faces of the carbonyl group.

MDN-0209: pale yellow oil; $[\alpha]^{26}_{D}$ -48.22(c 0.19w/v%, CH_2Cl_2); UV (DAD) λ_{max} 240nm; IR (ATR) υ cm^{-1}: 3357, 3041, 2959, 2927, 2872, 2728, 1671, 1453, 1377, 1263, 1143, 1089, 1005, 963, 841. HRMS *m/z* 277.1800 $[M+H-H_2O]^+$ (calcd. for $C_{17}H_{25}O_3^+$, 277.1798); 312.2171 $[M+NH_4]^+$ (calcd. for $C_{17}H_{30}NO_4^+$, 312.2169); 611.3555 $[2M+Na]^+$ (calcd. for $C_{34}H_{52}NaO_8^+$, 611.3554); for ^1H and ^{13}C NMR data, see Table S1.

Table S1. ^1H (500MHz) and ^{13}C (125MHz) NMR Data for Compounds 1 and 2 in CD_3OD

Position	**1**		**2**	
	δ_C, type	δ_H, mult. (*J* in Hz)	δ_C, type	δ_H, mult. (*J* in Hz)
1	61.1, C		66.2, C	
2	67.6, CH	4.43, s	66.7, CH	4.77, s
3	134.0, C		151.1, C	
4	131.5, C		131.2, C	
5	65.2, CH	4.61, s	198.1, C	
6	59.5, CH	3.21, br t (1.7)	60.2, CH	3.30, s
7a	30.9, CH_2	2.83, dd (15.2, 7.4)	29.9, CH_2	2.94, dd (15.4, 7.8)
7b		2.37, dd (15.2, 7.4)		2.45, dd (15.4, 7.2)
8	118.9, CH	5.23, tt (7.4, 1.4)	118.2, CH	5.22, tm (7.5, 1.4)
9	136.2, C		137.1, C	
10	18.1, CH_3	1.67, s	18.0, CH_3	1.67, br s
11	26.0, CH_3	1.74, s	25.9, CH_3	1.75, br s
12	126.9, CH	6.42, d (15.7)	122.0, CH	6.06, m
13	134.9, CH	6.08, dt (15.7, 7.0)	140.1, CH	6.05, m
14	36.6, CH_2	2.15, c (7.0)	36.8, CH_2	2.14, m
15	23.5, CH_2	1.47, m (7.4)	23.3, CH_2	1.46, m (7.3)
16	14.0, CH_3	0.94, t (7.4)	14.0, CH_3	0.94, t (7.5)
17a	59.9, CH_2	4.39, d (12.3)	60.4, CH_2	4.47, d (13.4)
17b		4.15, d (12.3)		4.32, d (13.4)

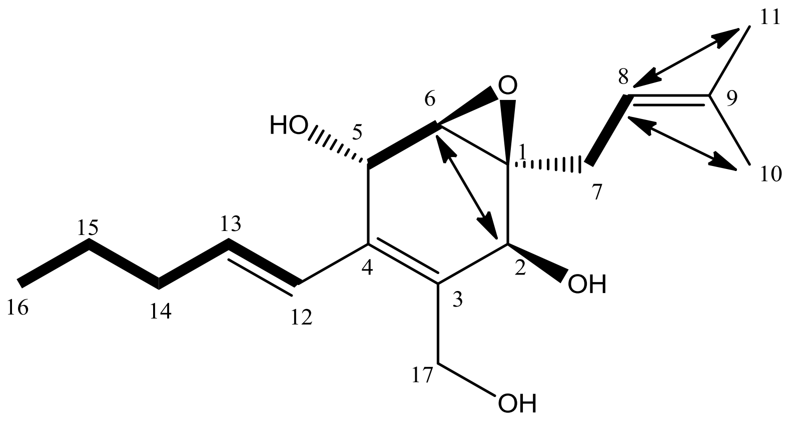

Figure S1. Key ^{1}H-^{1}H COSY (bold lines, along range H↔H) correlations for compound **1**

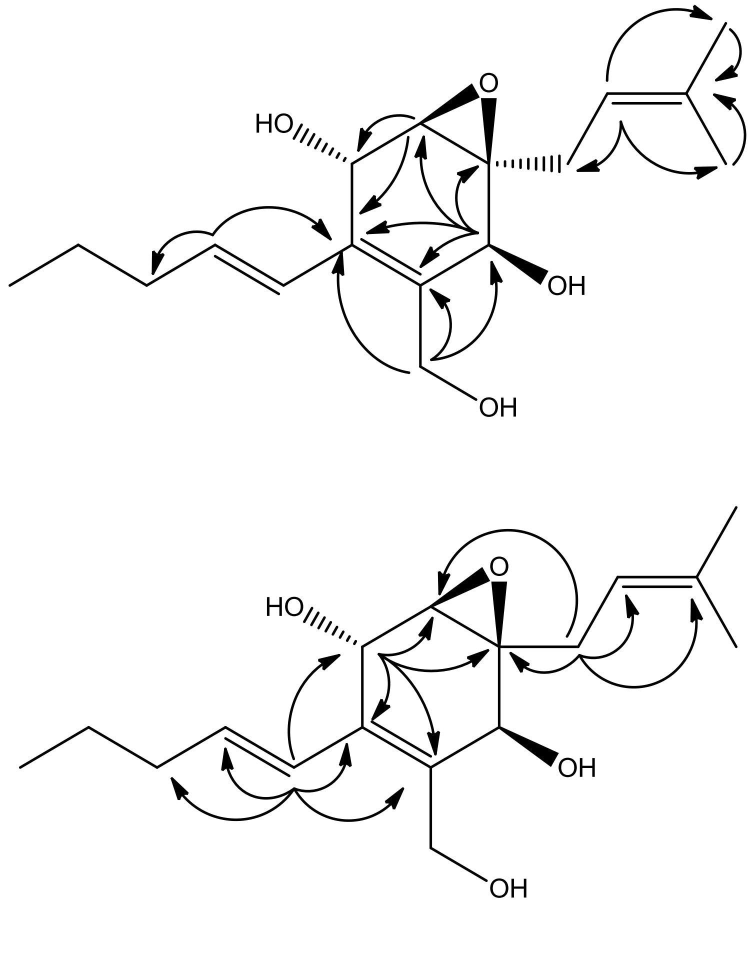

Figure S2. Key HMBC (H→C) correlations for compound **1**

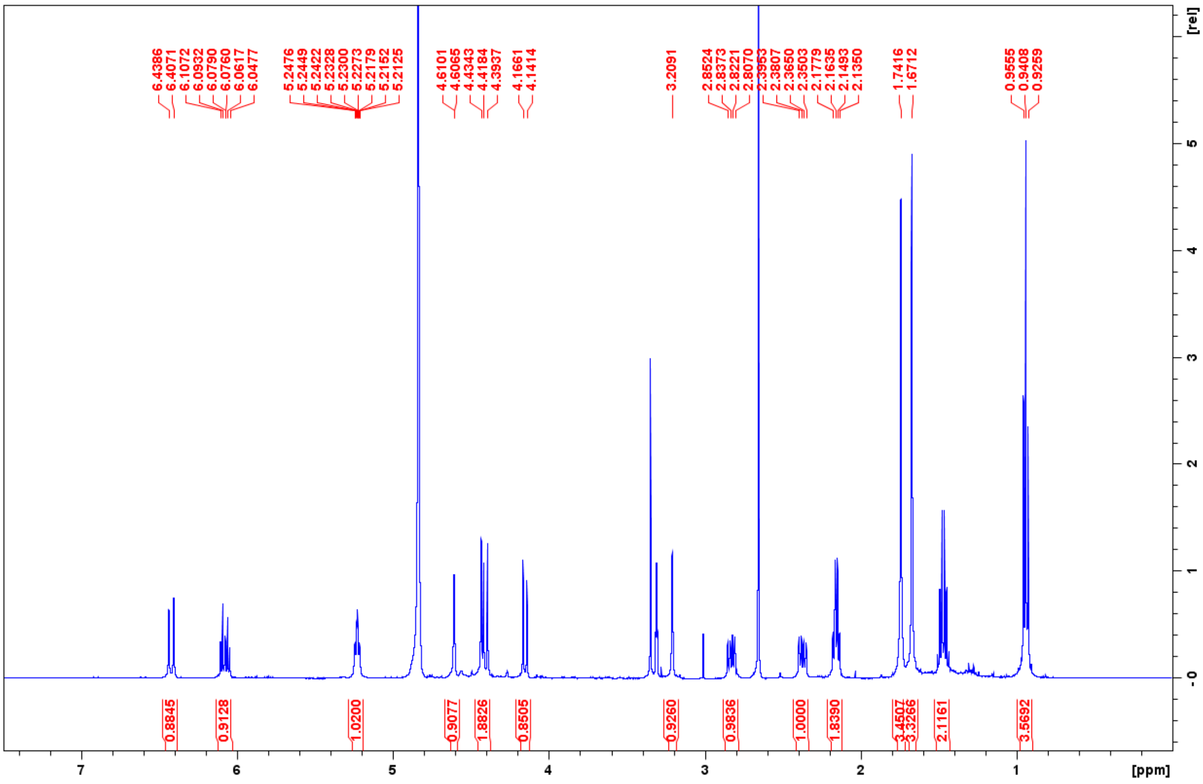

Figure S3. ^{1}H NMR spectrum (500MHz) of **1** in CD_3OD.

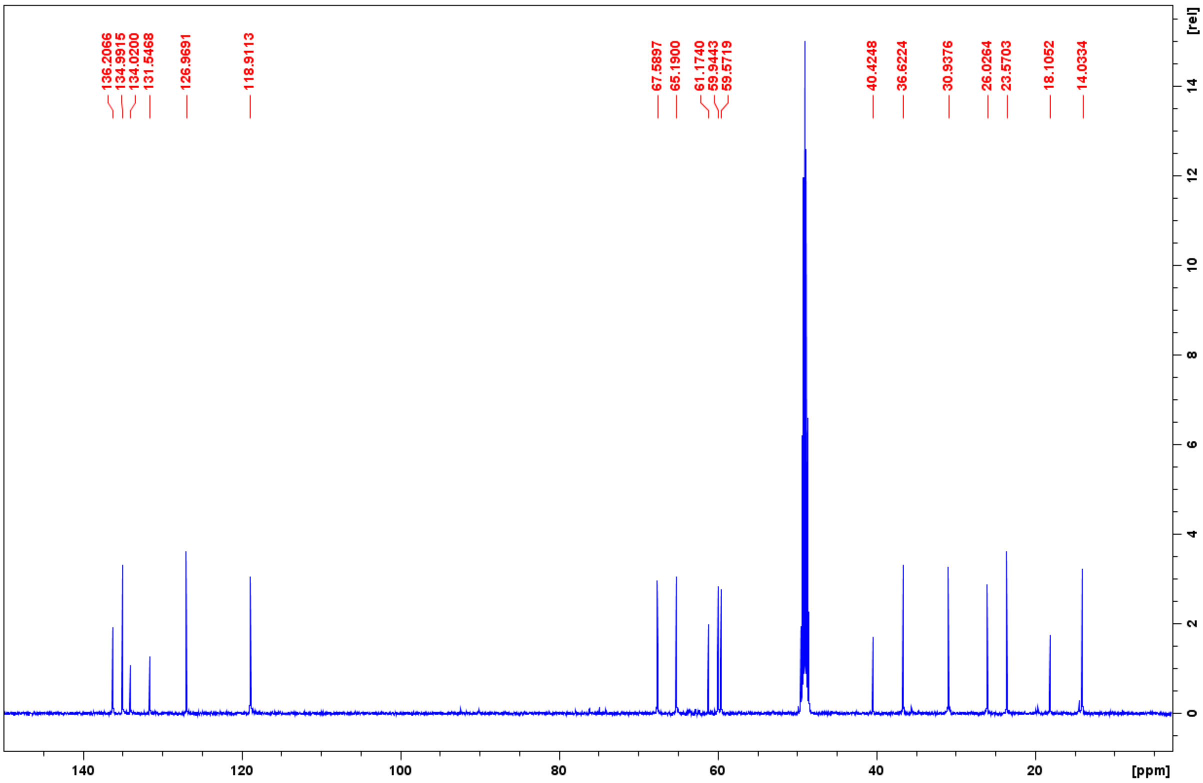

Figure S4. ^{13}C NMR spectrum (125MHz) of **1** in CD_3OD.

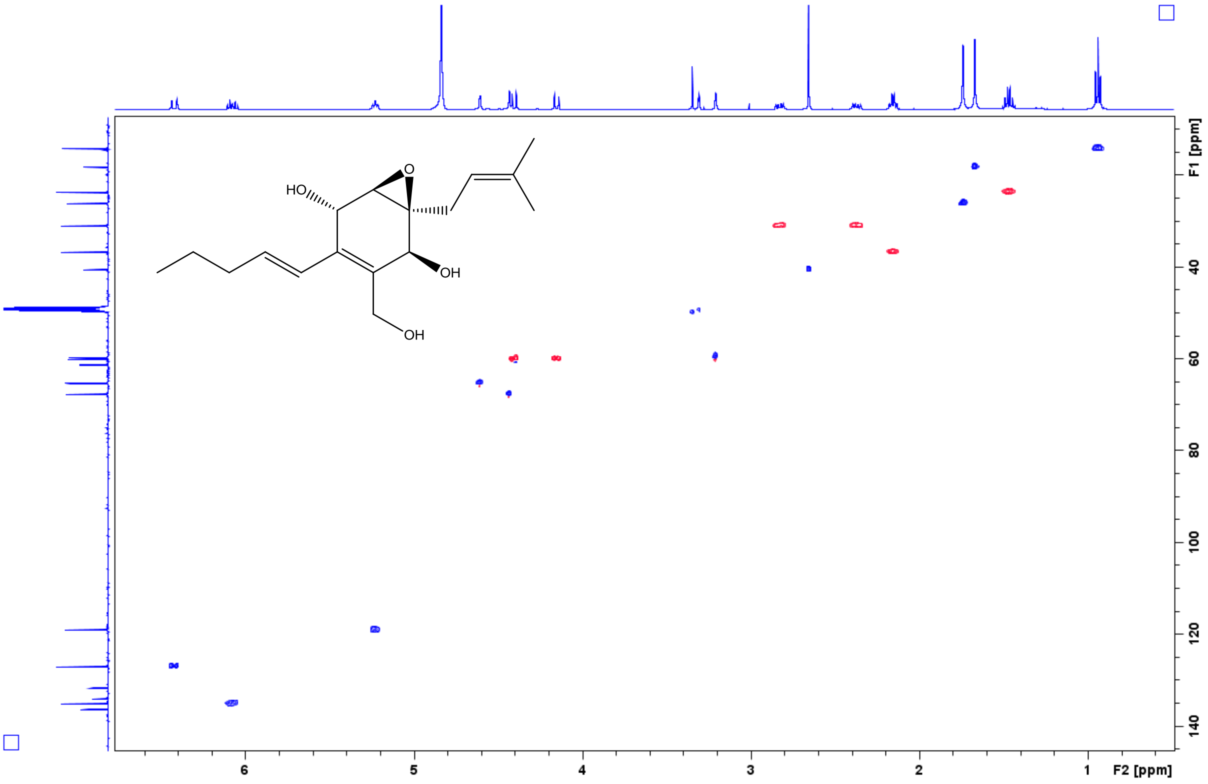

Figure S5. HSQC spectrum (500MHz) of **1** in CD_3OD.

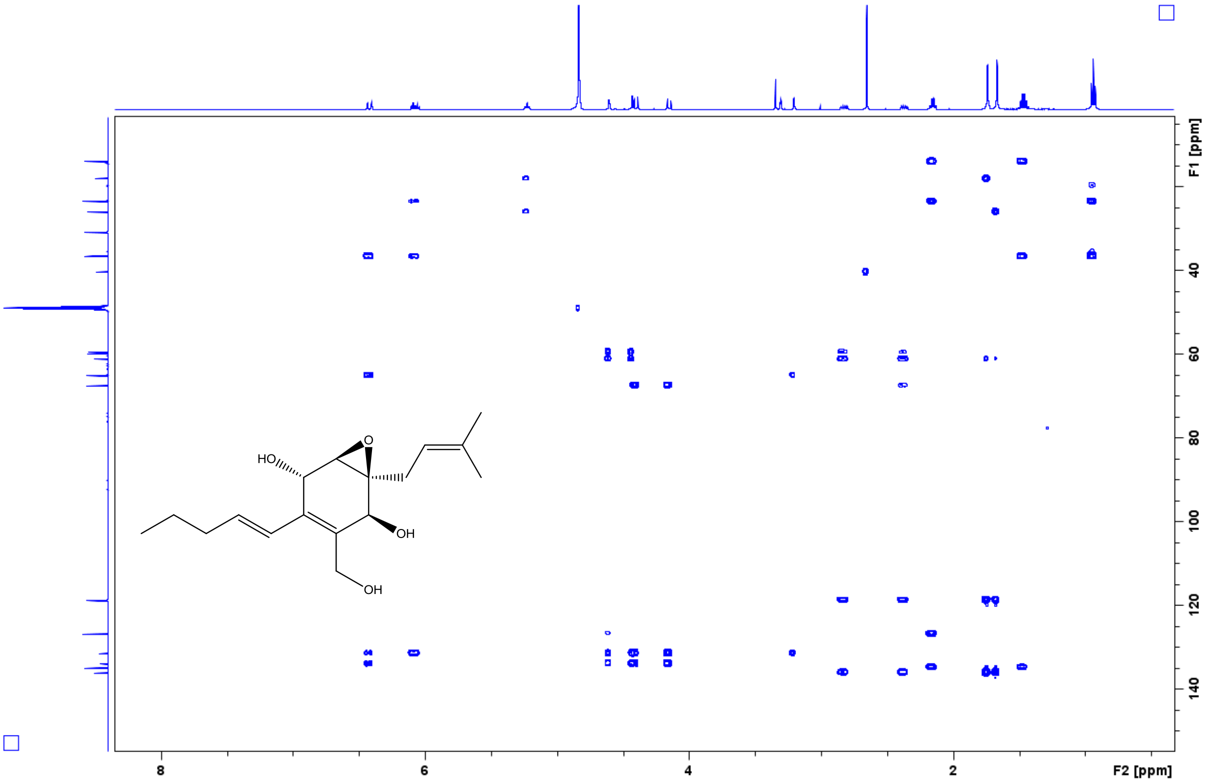

Figure S6. HMBC spectrum (500MHz) of **1** in CD_3OD.

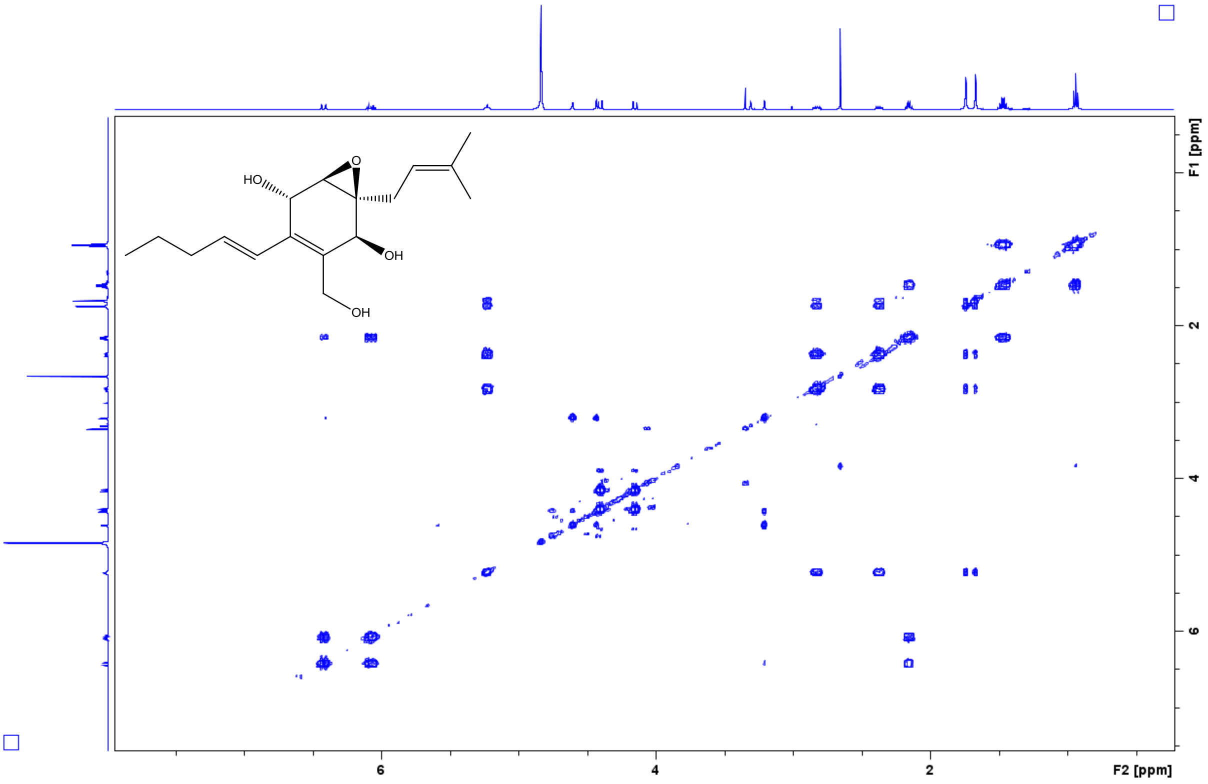

Figure S7. COSY spectrum (500MHz) of **1** in CD_3OD.

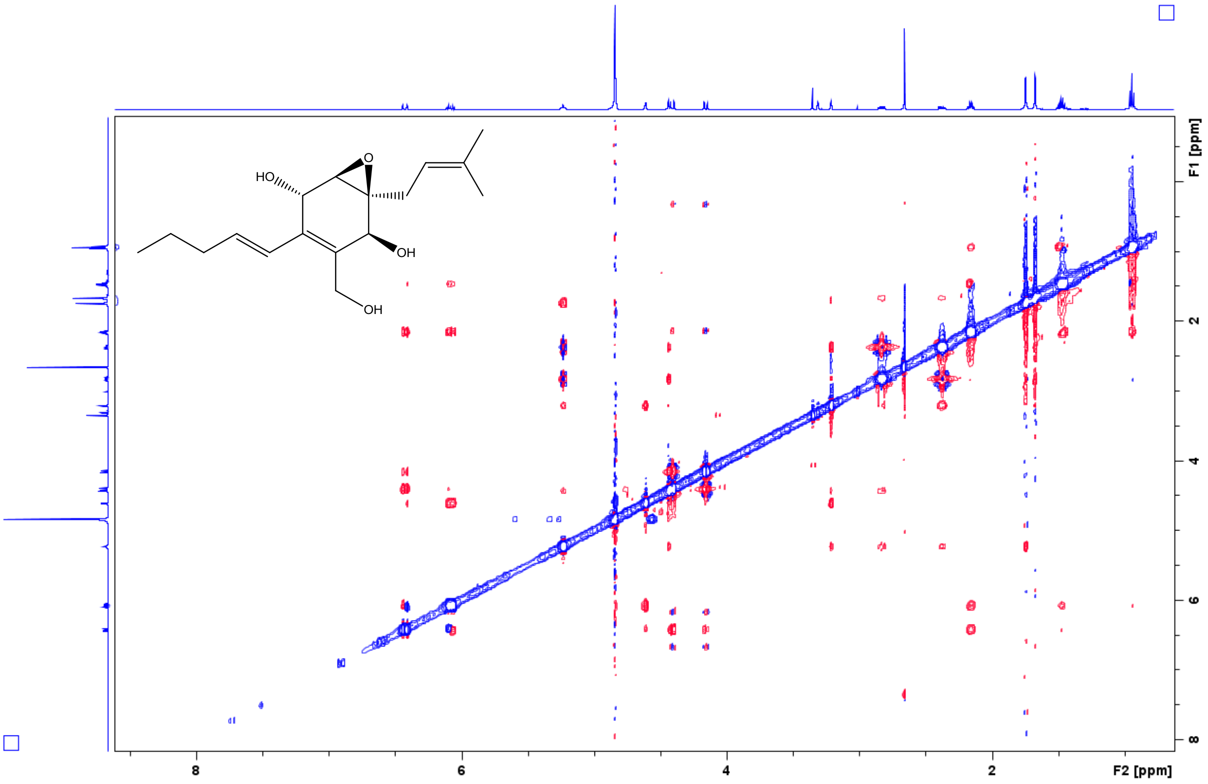

Figure S8. NOESY spectrum (500MHz) of **1** in CD_3OD.

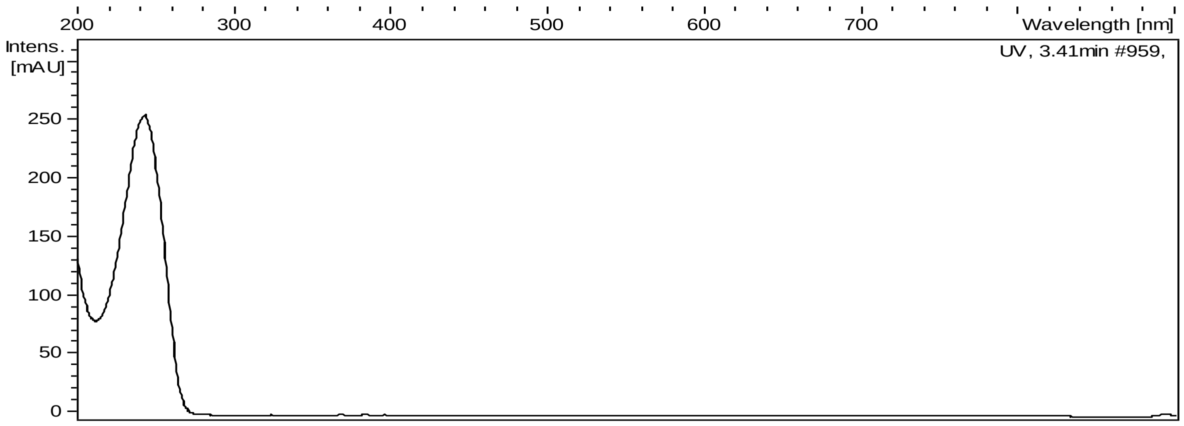

Figure S9. UV spectrum of **1**

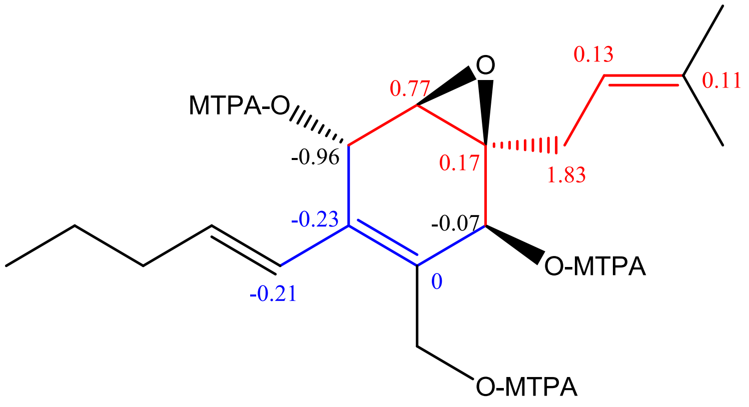

Figure S10. $\Delta\delta^{SR}$ values of MTPA-esters of compound **1**

MDN-0210 (2): White and amorphous solid; $[\alpha]^{26}_{D}$ 95.62 (c 0.04w/v%, CH_2Cl_2); UV (DAD) λ_{max} 210nm, 280nm; IR (ATR) υ cm^{-1}: 3420, 2959, 2928, 2872, 1672, 1453, 1377, 1025, 969, 841. HRMS *m/z* 275.1641 $[M+H-H_2O]^+$ (calcd. for $C_{17}H_{23}O_3^+$, 275.1642); 293.1747 $[M+H]^+$ (calcd. for $C_{17}H_{25}O_4^+$, 293.1747); 310.2007 $[M+NH_4]^+$ (calcd. for $C_{17}H_{28}NO_4^+$, 310.2013); 607.3234 $[2M+Na]^+$ (calcd. for $C_{34}H_{48}NaO_8^+$, 607.3241); for ^{1}H and ^{13}C NMR data, see Table S1.

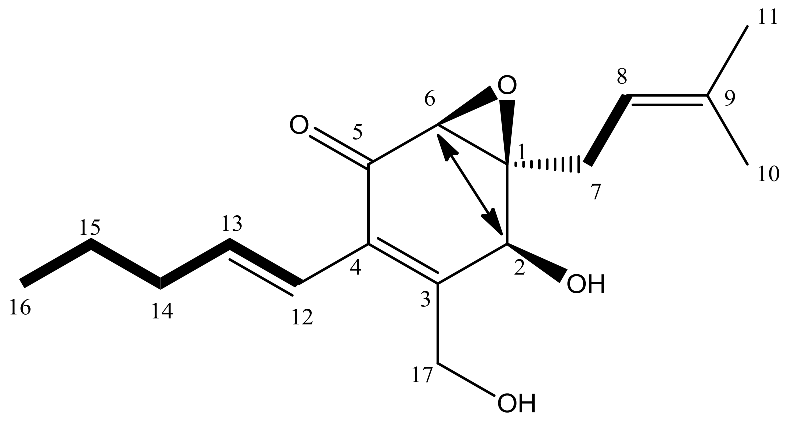

Figure S11. Key ^{1}H-^{1}H COSY (bold lines, and long range H↔H) correlations for compound **2**.

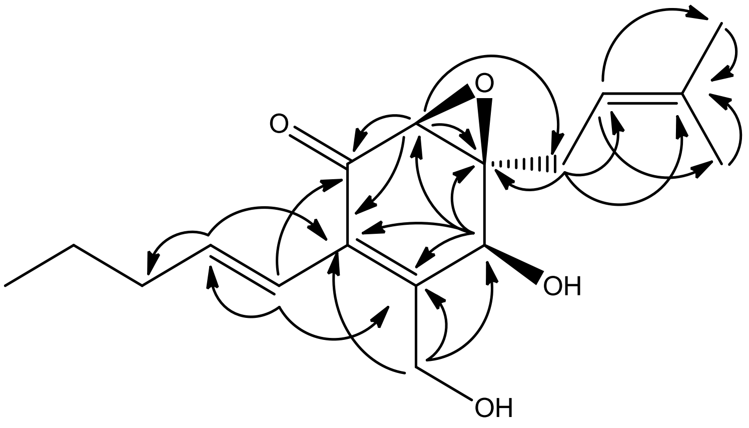

Figure S12. Key HMBC (H→C) correlations for compound **2**.

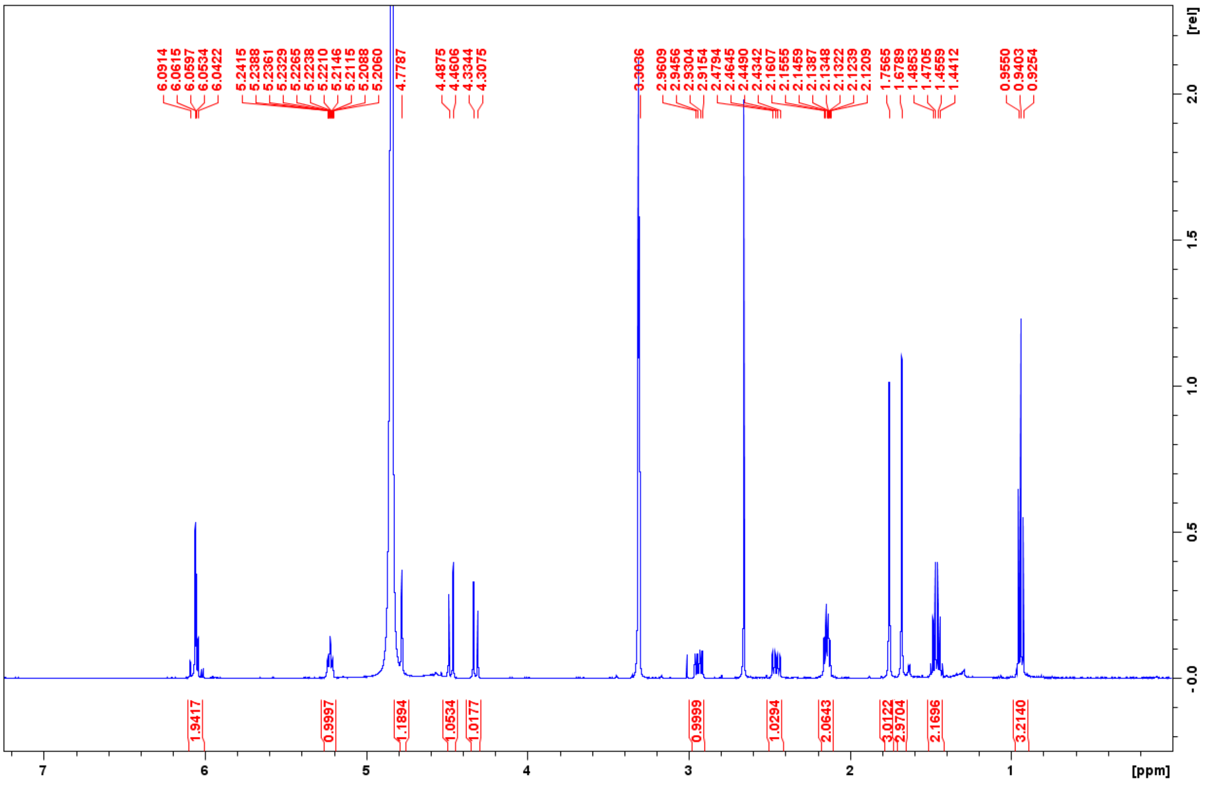

Figure S13. ^{1}H NMR spectrum (500MHz) of **2** in CD_3OD.

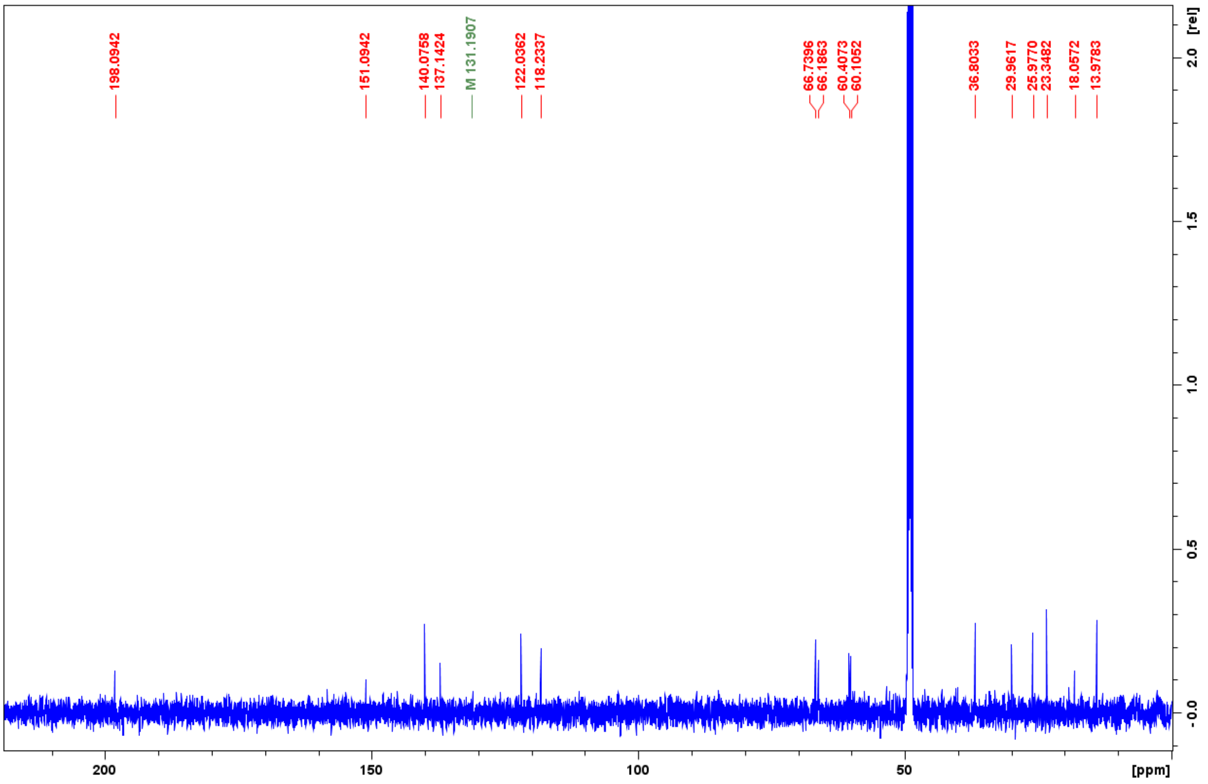

Figure S14. ^{13}C NMR spectrum (125MHz) of **2** in CD_3OD.

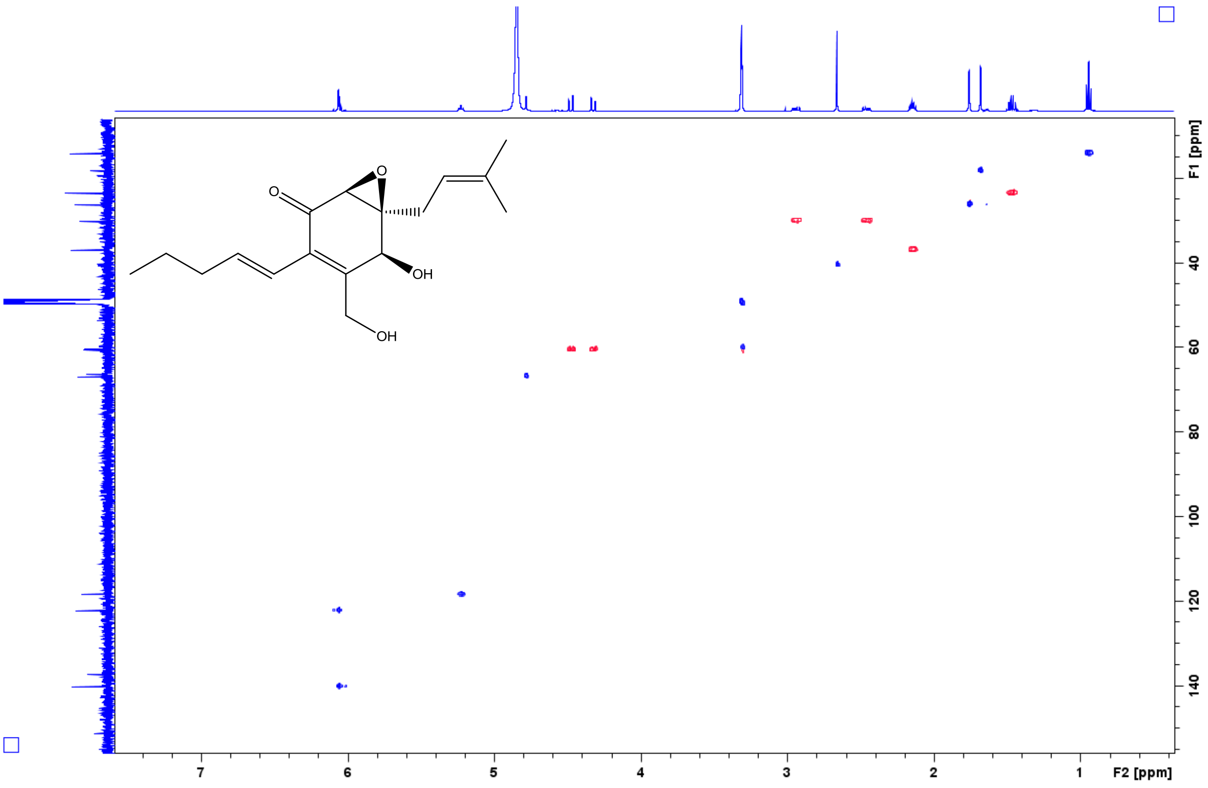

Figure S15. HSQC spectrum (500MHz) of **2** in CD_3OD.

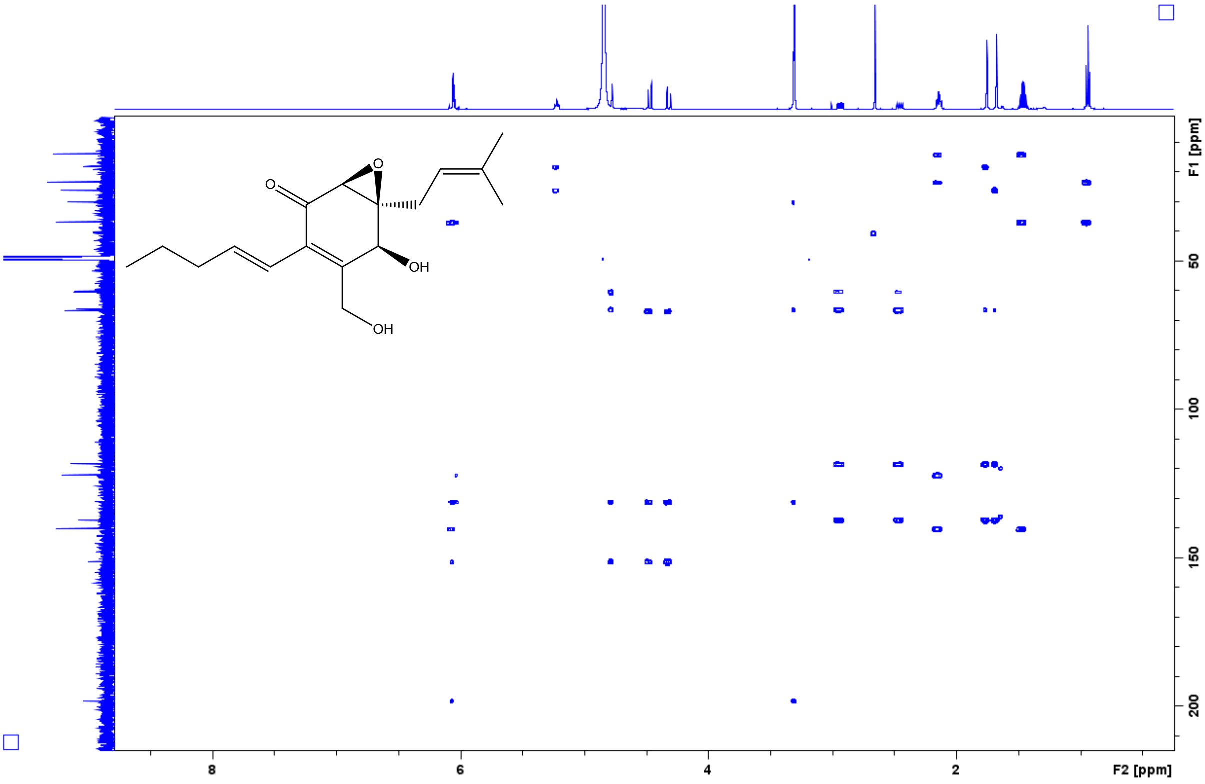

Figure S16. HMBC spectrum (500MHz) of **2** in CD_3OD.

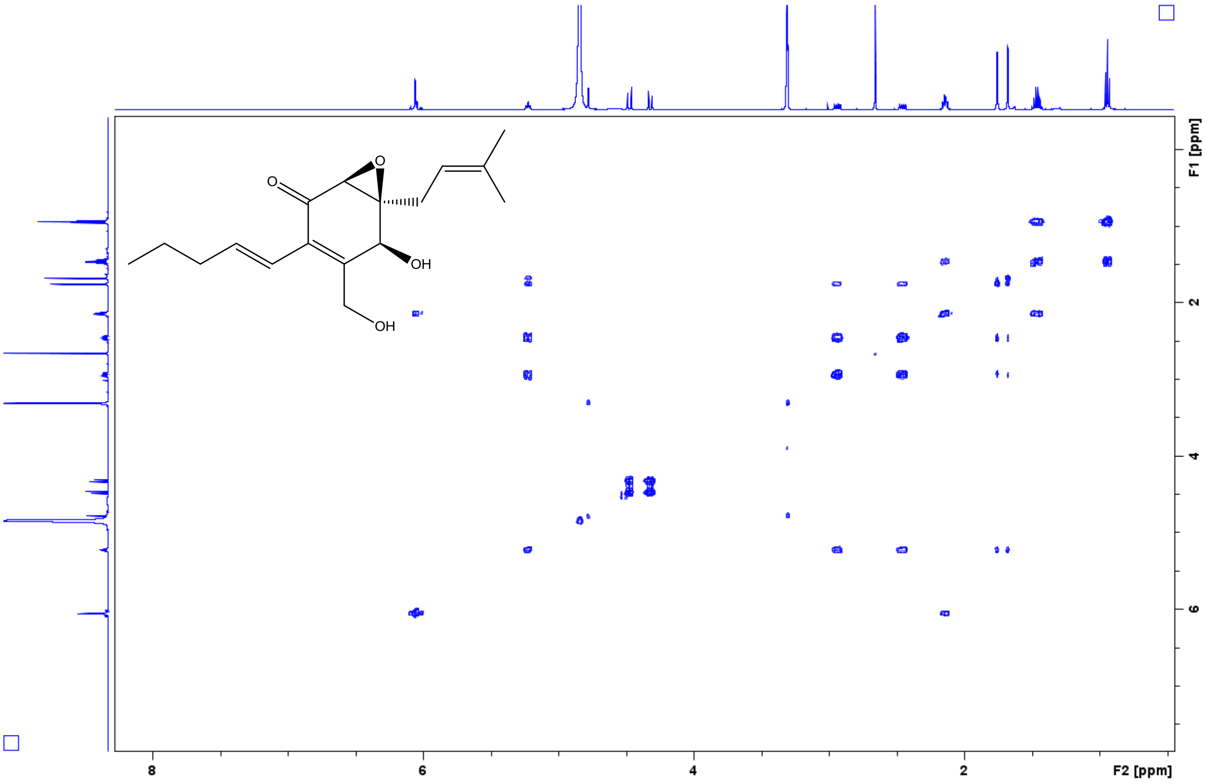

Figure S17. COSY spectrum (500MHz) of **2** in CD_3OD.

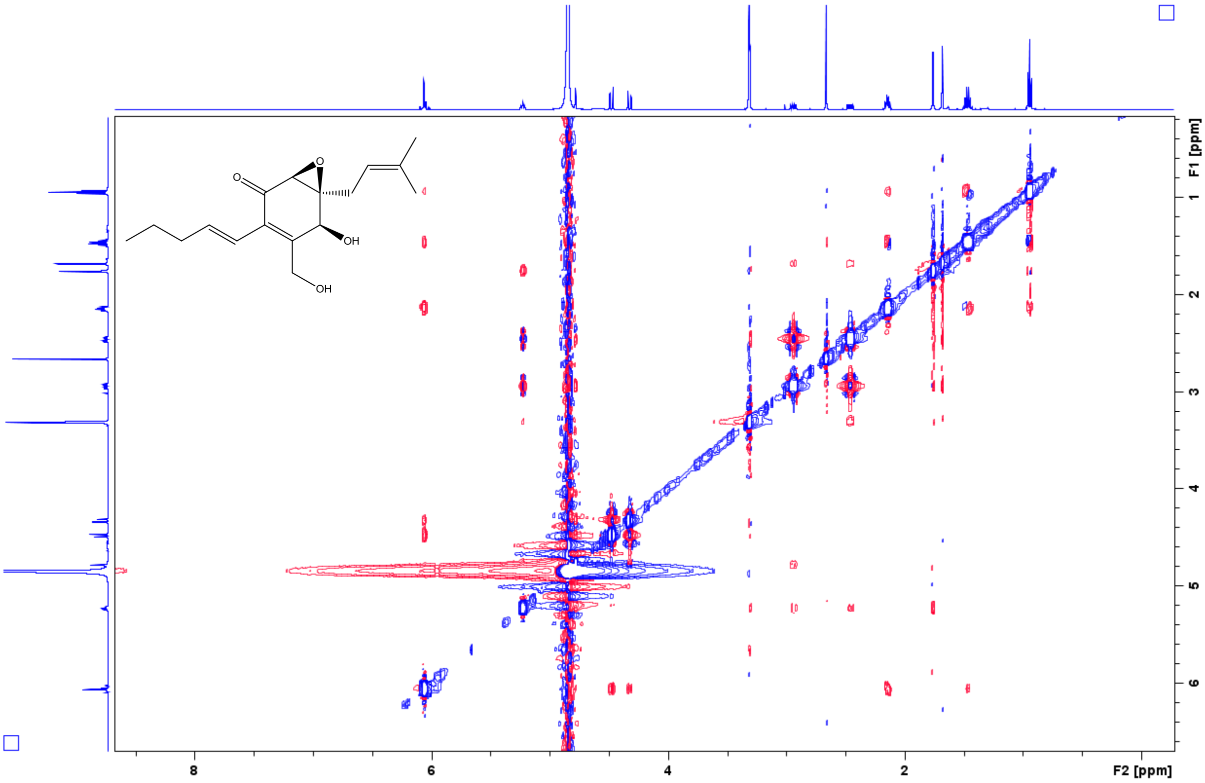

Figure S18. NOESY spectrum (500MHz) of **2** in CD_3OD.

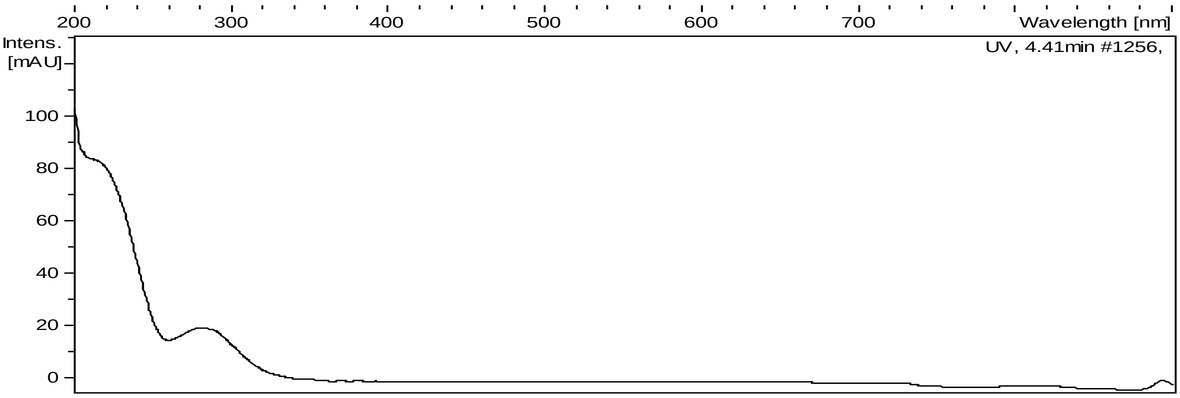

Figure S19. UV spectrum of **2**

MDN-0211 (3): White and amorphous solid; $[\alpha]^{25}_{D}$ 0.00 (c 0.16w/v%, CH_3OH); UV (DAD) λ_{max} 230nm, 280nm; IR (ATR) υ cm^{-1}: 3363, 2943, 2839, 1643, 1505, 1454, 1438, 1245, 1210, 1133, 1086, 1024. HRESIMS *m/z* 195.0655 $[M+H-H_2O]^+$ (calcd. for $C_{10}H_{11}O_4^+$, 195.0652); 235.0574 $[M+Na]^+$ (calcd. for $C_{10}H_{12}NaO_5^+$, 235.0577); 447.1257 $[2M+Na]^+$ (calcd. for $C_{20}H_{24}NaO_{10}^+$, 447.1262); for ^1H and ^{13}C NMR data, see Table S3:

Table S3. ^{1}H (500 MHz) and ^{13}C (125 MHz) NMR Data for Compound 1 in MeOD

Position	δ_C, type	δ_H, mult. (J in Hz)
1	107.6, CH	6.12, d (1.96)
2	118.6, C	
3	149.6, C	
4	98.1, CH	6.57, s
5	150.7, C	
6	135.1, C	
7	129.5, C	
8a	71.5, CH2	5.06, dd (12.8, 1.48)
8b		4.42, d (12.8)
9	56.6, CH3	3.81, s
10	57.1, CH3	3.88, s

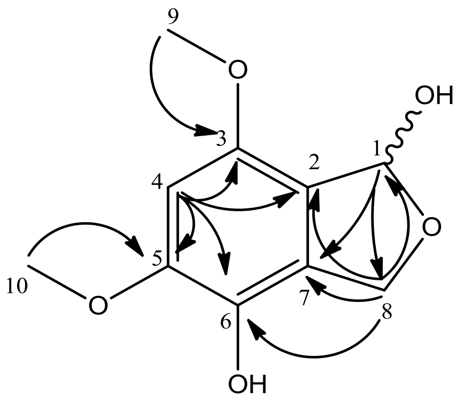

Figure S20. Key HMBC (H→C) correlations for compound **3**

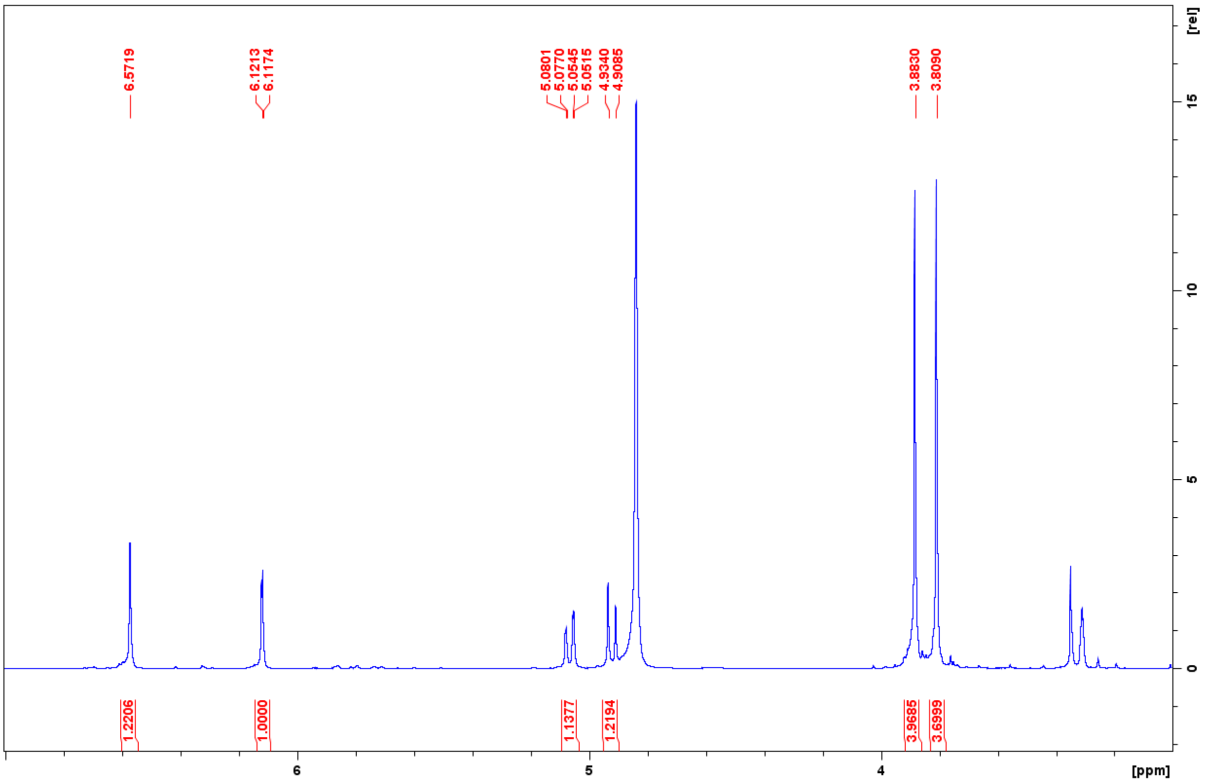

Figure S21. ^{1}H NMR spectrum (500MHz) of **3** in CD_3OD.

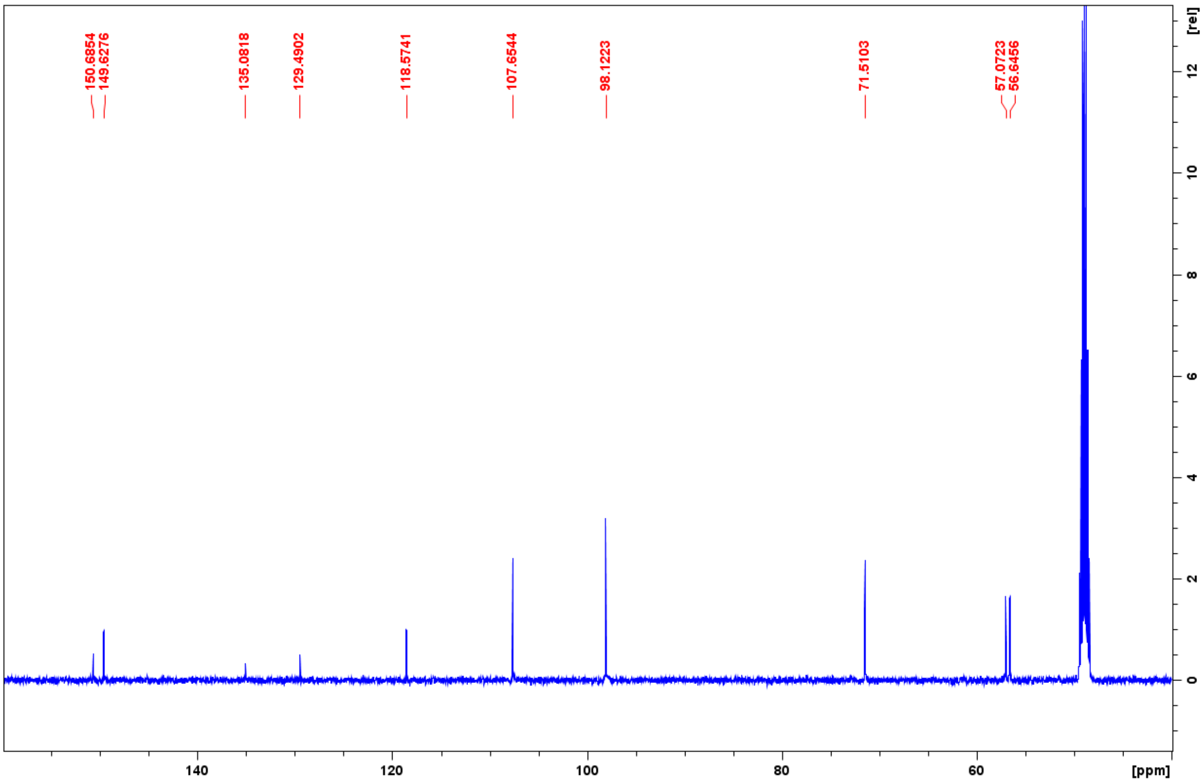

Figure S22. ^{13}C NMR spectrum (125MHz) of **3** in CD_3OD.

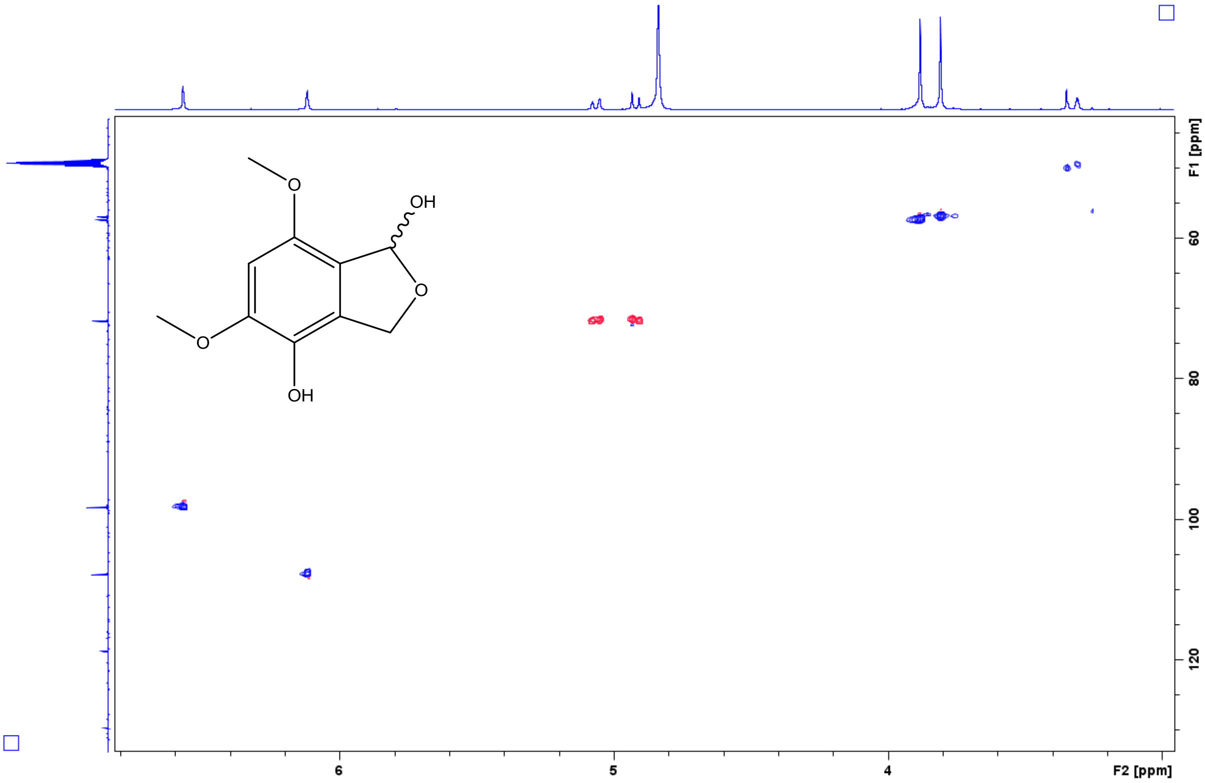

Figure S23. HSQC spectrum (500MHz) of **3** in CD_3OD.

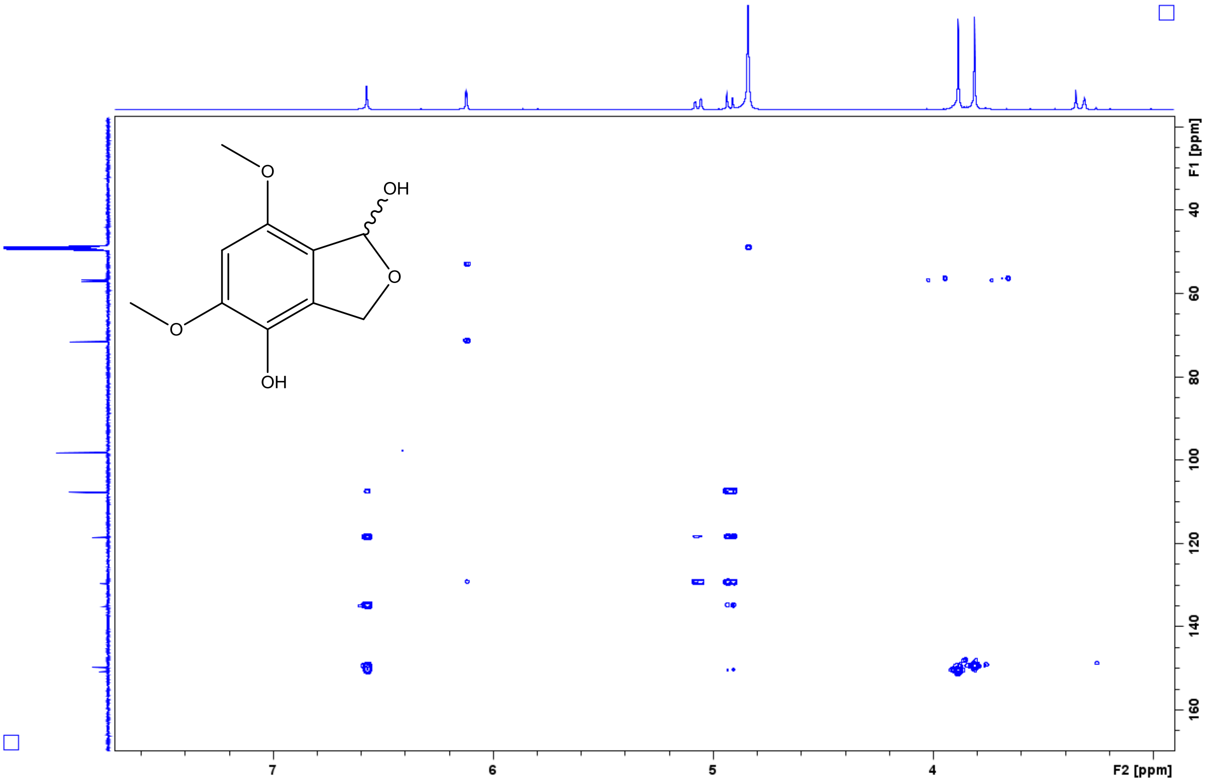

Figure S24. HMBC spectrum (500MHz) of **3** in CD_3OD.

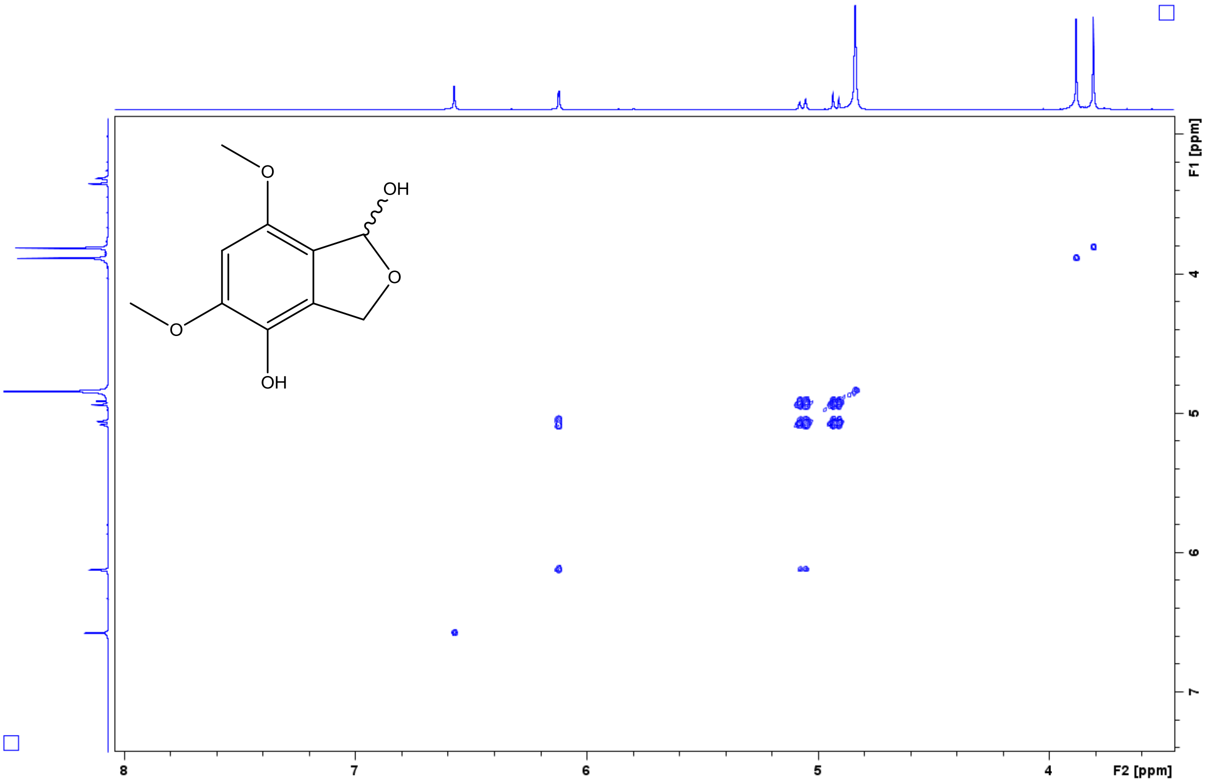

Figure S25. COSY spectrum (500MHz) of **3** in CD_3OD.

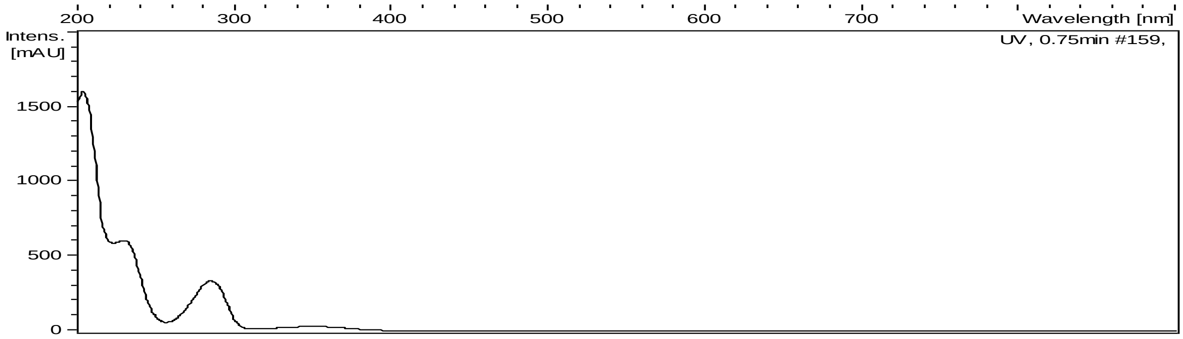

Figure S26. UV spectrum of **3**.

Reference for Supporting Information

1. Seco J.M., Quiñoá E. and Riguera R. The Assignment of Absolute Configuration by NMR. *Chemical Reviews* **104**, 17-117 (2004).
